# Fusion transcripts and their genomic breakpoints in polyadenylated and ribosomal RNA–minus RNA sequencing data

**DOI:** 10.1093/gigascience/giab080

**Published:** 2021-12-09

**Authors:** Youri Hoogstrate, Malgorzata A Komor, René Böttcher, Job van Riet, Harmen J G van de Werken, Stef van Lieshout, Ralf Hoffmann, Evert van den Broek, Anne S Bolijn, Natasja Dits, Daoud Sie, David van der Meer, Floor Pepers, Chris H Bangma, Geert J L H van Leenders, Marcel Smid, Pim J French, John W M Martens, Wilbert van Workum, Peter J van der Spek, Bart Janssen, Eric Caldenhoven, Christian Rausch, Mark de Jong, Andrew P Stubbs, Gerrit A Meijer, Remond J A Fijneman, Guido W Jenster

**Affiliations:** Department of Urology, Erasmus Medical Center Cancer Institute, Wytemaweg 80, Rotterdam 3015GD, The Netherlands; Department of Neurology, Erasmus Medical Center Cancer Institute, Wytemaweg 80, Rotterdam 3015GD, The Netherlands; Department of Pathology, Netherlands Cancer Institute, Amsterdam 3015GD, The Netherlands; Department of Urology, Erasmus Medical Center Cancer Institute, Wytemaweg 80, Rotterdam 3015GD, The Netherlands; Department of Life Sciences, Barcelona Supercomputing Center, Barcelona 08034, Spain; Department of Medical Oncology, Erasmus Medical Center, Rotterdam 3015GD, The Netherlands; Department of Urology, Erasmus Medical Center Cancer Institute, Wytemaweg 80, Rotterdam 3015GD, The Netherlands; Cancer Computational Biology Center, Erasmus Medical Center, Rotterdam 3015GD, The Netherlands; Hartwig Medical Foundation, Amsterdam 1098XH, The Netherlands; Philips Research, Eindhoven 5656AE, The Netherlands; Department of Pathology, Netherlands Cancer Institute, Amsterdam 3015GD, The Netherlands; Department of Pathology and Medical Biology, University Medical Center Groningen, Groningen 9713GZ, The Netherlands; Department of Pathology, Netherlands Cancer Institute, Amsterdam 3015GD, The Netherlands; Department of Urology, Erasmus Medical Center Cancer Institute, Wytemaweg 80, Rotterdam 3015GD, The Netherlands; Department of Pathology, Netherlands Cancer Institute, Amsterdam 3015GD, The Netherlands; GenomeScan, Leiden 2333BZ, The Netherlands; GenomeScan, Leiden 2333BZ, The Netherlands; Department of Urology, Erasmus Medical Center Cancer Institute, Wytemaweg 80, Rotterdam 3015GD, The Netherlands; Department of Pathology, Erasmus Medical Center, Rotterdam 3015GD, The Netherlands; Department of Medical Oncology, Erasmus Medical Center, Rotterdam 3015GD, The Netherlands; Department of Neurology, Erasmus Medical Center Cancer Institute, Wytemaweg 80, Rotterdam 3015GD, The Netherlands; Department of Medical Oncology, Erasmus Medical Center, Rotterdam 3015GD, The Netherlands; Limes Innovations, Ruigekade 1, Leiderdorp 2351SX, The Netherlands; Department of Pathology, Erasmus Medical Center, Rotterdam 3015GD, The Netherlands; GenomeScan, Leiden 2333BZ, The Netherlands; Lygature, Utrecht 3521AL, The Netherlands; BioLizard N.V., Ghent 9000, Belgium; VHLGenetics, Wageningen 6708PW, The Netherlands; Department of Pathology, Erasmus Medical Center, Rotterdam 3015GD, The Netherlands; Department of Pathology, Netherlands Cancer Institute, Amsterdam 3015GD, The Netherlands; Department of Pathology, Netherlands Cancer Institute, Amsterdam 3015GD, The Netherlands; Department of Urology, Erasmus Medical Center Cancer Institute, Wytemaweg 80, Rotterdam 3015GD, The Netherlands

**Keywords:** gene fusion, RNA precursors, RNA-seq, chromosome breakage, genomic structural variation, cryptic exons, TMPRSS2-ERG

## Abstract

**Background:**

Fusion genes are typically identified by RNA sequencing (RNA-seq) without elucidating the causal genomic breakpoints. However, non–poly(A)-enriched RNA-seq contains large proportions of intronic reads that also span genomic breakpoints.

**Results:**

We have developed an algorithm, Dr. Disco, that searches for fusion transcripts by taking an entire reference genome into account as search space. This includes exons but also introns, intergenic regions, and sequences that do not meet splice junction motifs. Using 1,275 RNA-seq samples, we investigated to what extent genomic breakpoints can be extracted from RNA-seq data and their implications regarding poly(A)-enriched and ribosomal RNA–minus RNA-seq data. Comparison with whole-genome sequencing data revealed that most genomic breakpoints are not, or minimally, transcribed while, in contrast, the genomic breakpoints of all 32 *TMPRSS2*-*ERG*–positive tumours were present at RNA level. We also revealed tumours in which the *ERG* breakpoint was located before *ERG*, which co-existed with additional deletions and messenger RNA that incorporated intergenic cryptic exons. In breast cancer we identified rearrangement hot spots near *CCND1* and in glioma near *CDK4* and *MDM2* and could directly associate this with increased expression. Furthermore, in all datasets we find fusions to intergenic regions, often spanning multiple cryptic exons that potentially encode neo-antigens. Thus, fusion transcripts other than classical gene-to-gene fusions are prominently present and can be identified using RNA-seq.

**Conclusion:**

By using the full potential of non–poly(A)-enriched RNA-seq data, sophisticated analysis can reliably identify expressed genomic breakpoints and their transcriptional effects.

## Background

Genomic rearrangements are frequently observed in cancer and can drive disease initiation and progression through disruption of tumour suppressor genes and activation of oncogenes [1–3]. Marked examples include *TMPRSS2-ERG* fusions in prostate adenocarcinoma (PCa) [4] and *BCR-ABL* in chronic myelogenous leukaemia [5]. DNA rearrangements and their aberrant ligations are identified as genomic breakpoints by whole-genome sequencing (WGS), but their potential role as driver mutation is mostly unresolved as of yet. The majority of genomic breakpoints involve intergenic regions and are thus typically not located in messenger RNA (mRNA) and protein-coding sequences [6]. Moreover, genomic breakpoints of fusion genes are mostly located intronically [7]. To reveal their downstream effects, RNA sequencing (RNA-seq) is crucial to investigate changes at the transcriptional level and identify actual (in-frame) fusion transcripts. Conversely, for fusion transcripts, identification of the exact genomic breakpoint(s) can be essential to explain changes in gene expression and to define the origins of alternative promoter use and altered splicing or polyadenylation events. Combined genomic and expression data enables further study of the functional consequences of genomic rearrangements and signifies whether an event is merely a passenger or a putative driver mutation [7, 8]. However, for many transcriptome studies, the exact genomic breakpoints of expressed rearrangements have not been resolved because matched WGS, Sanger sequencing, or similar analyses were not performed. Therefore, we set out to determine whether genomic breakpoints could be identified from RNA-seq data.

Next to targeted gene approaches, there are 2 main approaches in preparing RNA-seq libraries [9]. First, the more traditional method includes the positive selection of polyadenylated (poly(A)^+^) mRNA to specifically target mRNA and eliminate abundant ribosomal RNA (rRNA). Because splicing takes place mostly co-transcriptionally, pre-mRNA is typically not polyadenylated and thus not included in this approach. Alternatively, one can extract total RNA and use random hexamer primers to initiate complementary DNA synthesis while removing abundant unwanted RNAs by various additional methods. This approach is referred to as rRNA-minus and is commonly applied when (partially) degraded RNA from formalin-fixed paraffin-embedded (FFPE) samples is sequenced.

rRNA-minus RNA-seq is thus capable of identifying non-poly(A) transcripts such as circular RNAs (circRNAs), specific types of small and long non-coding RNAs, and, importantly, actively transcribed precursor mRNAs (pre-mRNAs) [10]. Although the exact numbers depend on the protocol being used, tissue type, lariats [11], and intron lengths, typically 30–40% of rRNA-minus RNA-seq reads map to intronic features, compared with 5–10% in poly(A)^+^ RNA-seq [12]. Therefore, rRNA-minus RNA-seq datasets require ≥50% higher sequencing depth to achieve an exon coverage comparable to poly(A)^+^ RNA-seq, while being capable of identifying additional RNA classes [9].

Fusion genes such as *TMPRSS2-ERG* and *BCR-ABL* are frequently observed as drivers within their respective malignant tissue [13]. Yet, many observed fusion genes are still of unknown consequence and seen in small frequencies in various cancer types. RNA-seq is highly suitable for fusion gene detection [14–16].

Methods to integrate RNA fusions with genomic breakpoints allow further assessment of functional consequences [7, 8, 17]. They are even capable of integrating complex higher order rearrangements but remain dependent on the availability of matching DNA data. State-of-the-art fusion-detection tools such as FusionMap, FusionCatcher, and JAFFA focus on exon regions or splice junctions specifically [18–20], which are the main target of poly(A)^+^ RNA-seq. Indeed, these tools also work well on rRNA-minus RNA-seq because these also include exonic reads. Their efficient search space reduction in turn reduces the overall complexity and processing time. However, using rRNA-minus RNA-seq, typically 30–40% of the aligned reads are intronic and a further 20–25% of all reads are found to be intergenic [12], which are often a priori neglected. This large proportion of intronic and intergenic reads provides an opportunity to identify additional cancer-specific transcripts and exact genomic breakpoints of fusion genes. We have shown in a proof-of-concept that rRNA-minus RNA-seq can indeed identify genomic breakpoints [10].

Here, we leverage a novel algorithm named Dr. Disco to report on the presence of genomic breakpoints in RNA-seq data, its implications regarding poly(A)^+^ and rRNA-minus RNA-seq, and on fused cryptic exons. The algorithm computationally identifies such genomic breakpoints and exon-to-exon junctions in a genome-wide fashion, taking into account the potential of rRNA-minus RNA-seq. It was applied on 6 RNA-seq datasets spanning multiple malignant tissue types (n = 1,275) (Table 1). Indeed, we reveal exact causal genomic breakpoints as derived from RNA-seq alone but limited to regions sufficiently expressed such as fusion gene *TMPRSS2-ERG*. Furthermore, rRNA-minus RNA-seq data can reveal more transcriptionally active rearrangements than poly(A)^+^ RNA-seq and results can be useful to supplement WGS. While only large datasets were analysed in this study, the method is developed for single-sample analysis. In summary, rRNA-minus RNA-seq in combination with a suited analysis pipeline gives a more complete view on both the origin and effects of genomic rearrangements and their direct influence on the expression of associated genes.

**Table 1:** Dataset overview

Dataset	Tissue	Sequencing	n	Depth	Reference	Source	Comments
NGS-ProToCol	Prostate cancer	rRNA− RNA S	41	∼70M	EGAS00001002816	[42]	
NGS-ProToCol	Normal prostate	rRNA− RNA S	51	∼70M	EGAS00001002816	[42]	
NGS-ProToCol	Colon cancer	rRNA− RNA S	30	∼70M	EGAS00001002854	[21, 22, 42]	
NGS-ProToCol	Colon adenoma	rRNA− RNA S	30	∼70M	EGAS00001002854	[21, 22, 42]	
NGS-ProToCol	Normal colon	rRNA− RNA S	18	∼70M	EGAS00001002854	[21, 22, 42]	
BASIS	Breast cancer	rRNA− RNA S	289	∼150M	EGAS00001001178	[26, 27]	
PCa-LINES	Prostate cancer	rRNA− RNA S	6 (+1)*	∼37M	EGAS00001001476		*NGS-ProToCol 7046-004-052 matches PCa-LINES G-110
PCa-LINES	Prostate cancer	poly(A)^+^ U	7	∼50M	EGAS00001001476	[55]	
PCMM-FFPE	Prostate cancer	rRNA− RNA S	529	∼40M			Shallow FFPE
CGGA	Glioma	rRNA− RNA U	274	∼30M	GSE48865	[28]	
ENCODE MCF-7	Breast cancer	poly(A)^+^ S	1	∼138M	SRR534293	[20, 29]	
BASIS	Breast cancer	WGS DNA	560	∼40×	EGAS00001001178	[25]	
PCa-LINES	Prostate cancer	WGS DNA	7	∼100×	EGAS00001001476	[24]	
Weier	Prostate cancer	TMPRSS2-ERG targeted DNA	29			[39]	No raw data were used for determining WGS coverage
Pleasance	Melanoma	WGS DNA	9	∼40×	EGAS00000000052	[56]	No raw data were used for determining WGS coverage

RNA sequencing datasets are given a suffix indicating whether they were performed stranded (S) or unstranded (U); rRNA− indicates rRNA-minus. *The PCa-LINES dataset consists of 7 samples of which sample G-110 is rRNA-minus RNA-sequenced as part of the NGS-ProToCol study. Therefore the PCa-LINES dataset contributes 7 samples, from 6 unique patients, to the study.

## Data Description

RNA-seq data from several types of malignant tissue were used. For the NGS-ProToCol datasets (normal adjacent prostate, n = 41; prostate cancer, n = 51; normal adjacent colon, n = 18; colorectal adenoma, n = 30; and colorectal carcinoma, n = 30), carcinoma, adenoma (only colon), and adjacent normal tissue were rRNA-minus sequenced to study condition-specific molecular differences and further stratify tumour types [21, 22]. The PCa-LINES dataset consists of PCa cell lines PC346C and VCaP and additional PCa patient samples G-089, G-110, G-295, G-316, and G-346, which were WGS, rRNA-minus RNA, and poly(A)^+^ RNA sequenced. The included VCaP cell line is commonly used as a model system for prostate cancer and is known to contain the *TMPRSS2-ERG* fusion [23, 24]. The BASIS dataset consists of 560 WGS sequenced breast cancer (BrCa) samples [25] and 289 rRNA-minus RNA samples [26, 27], of which 207 are matching. The Chinese Glioma Atlas (CGGA) is composed of 274 rRNA-minus RNA-seq samples of various types of gliomas [28]. MCF-7 cell line data from ENCODE [29] were used for validation because it is a commonly used gold standard dataset [20]. We made the NGS-ProToCol and PCa-LINES publicly available; other data were taken from the public domain (Table 1).

**Figure 1:**
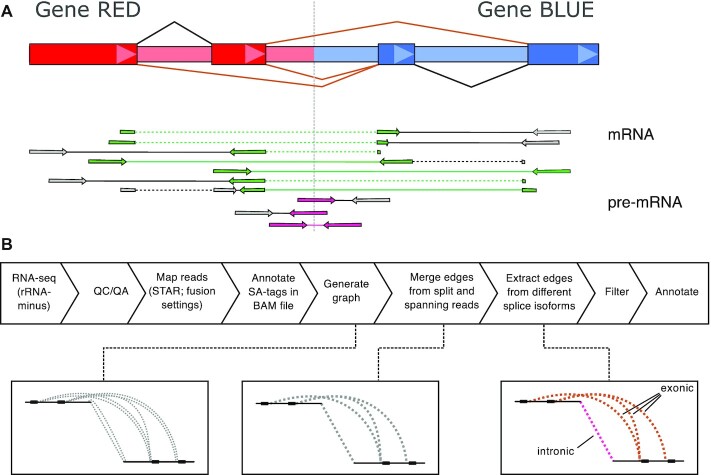
Overview Dr. Disco pipeline and principle. (**A**) Schematic representation of fusion-gene RED-BLUE. Owing to relatively large intron sizes, in-gene genomic breakpoints occur most often intronically. The fusion results in different fusion splice isoforms (brown splice junction annotation in fusion gene structure). Fusion splice junction spanning reads form the classical evidence for mature mRNA fusion events (reads marked in green). In rRNA-minus data, intronic pre-mRNA reads (reads marked in pink) may cover causal genomic breakpoints. (**B**) Pipeline flow chart: RNA-seq data is aligned. Discordant reads are transformed into edges and inserted into a breakpoint graph. In the graph, intronic or exonic derived edges are kept separate. Detection of junctions is performed by analysing the graph for clusters. An additional splice variant correction is applied. Identified junctions are filtered, annotated, and marked intronic or exonic. QC/QA: quality control/quality assurance.

To identify exact genomic breakpoints from rRNA-minus RNA-seq, we developed and implemented a novel algorithm, termed Dr. Disco. Briefly, it uses reads with a split alignment or read pairs with an inverted orientation or with a large insert size: discordant reads [30]. It uses reads not only from exons but also intronic and intergenic regions (Fig. 1 and Supplementary Dr. Disco Technical Specification). Discordant reads are transformed and inserted into a breakpoint graph [7]. The breakpoint graph, which contains junctions derived from RNA data only, is then extensively analysed to find clusters, resolve splicing, and keep junctions from distinct events separated.

For terminology, we define exon-to-exon splice fusion junctions (exonic junctions) as junctions that result from splicing and of which it can be expected that they could be detected by classical fusion detection algorithms. These also include fusions to not annotated (cryptic) exons.

Fusion transcripts that are not a result of (cryptic) exon-to-exon splicing are typically intron-to-intron junctions, spanning genomic breakpoints. Note that it is possible that genomic breakpoints are located within exons and do not result in fused spliced junctions (Supplementary Fig. S1). Because intron-to-intron junctions are not the product of splicing and are not the primary target of classical fusion gene detection, we consider these intronic. After the graph is analysed, corresponding detected junctions are marked "exonic" or "intronic" accordingly. The detailed computational methodology is described in Supplementary Methods and Supplementary Dr. Disco Technical Specification. The method was used to perform analyses in particular to study junctions in RNA data beyond classical fusion genes.

## Findings

### Evaluation of poly(A)+ detectors

**Figure 2:**
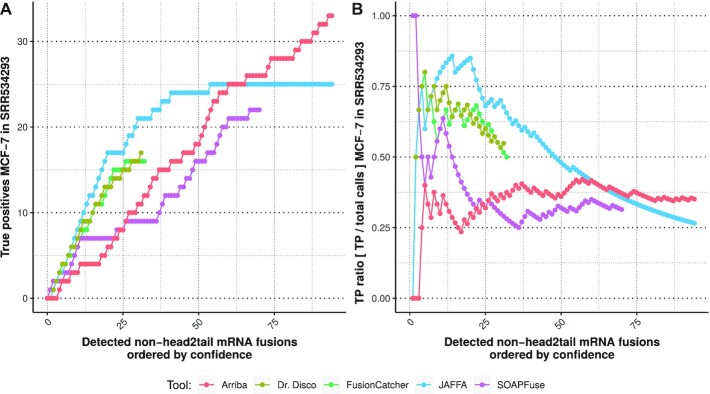
Evaluation on ENCODE MCF-7 dataset [20, 29]. For our algorithm, only non–head-to-tail junctions located on both sides at annotated exons were included. TP: true positive.

The performance of the algorithm identifying mRNA fusions was assessed by analysing the ENCODE MCF-7 dataset, which was subsequently compared to poly(A)^+^ detector results published earlier [20] (Fig. 2) and with Arriba [31], which also makes use of STAR as aligner. Our method was not superior to JAFFA, and performed rather similarly to FusionCatcher. It was mostly limited in the total number of true-positive results identified, indicating that it is more conservative than JAFFA, SOAPFuse, and Arriba. Although the true-positives ratio for Arriba was considerably lower, the total amount of true-positive results was the highest.

Interestingly, unreported fusions spanning cryptic exons and intergenic regions were observed, including *ATXN7*-chr1:106,216,304, resulting in a fusion to cryptic intergenic exons supported by a matching genomic breakpoint (chr3:6,394,8014-chr1:106,192,959), and *PRPF18-BEND7*, resulting in anti-sense transcription of *BEND7* spanning cryptic in-gene exons (Supplementary Fig. S2).

Dr. Disco’s performance of detecting only mRNA exon junctions was comparable but not superior to existing tools, while it revealed 27 additional high-confidence junctions using cryptic exons (Supplementary Table S1). The time it took Dr. Disco to complete analysis after the STAR alignment was 949 seconds, 2.07 times slower compared with Arriba (458 seconds). This is in concordance with the expectation that analysing a larger search space requires more conservative filtering and takes more resources to complete.

### Comparison of poly(A)+ and rRNA-minus RNA-seq

**Figure 3:**
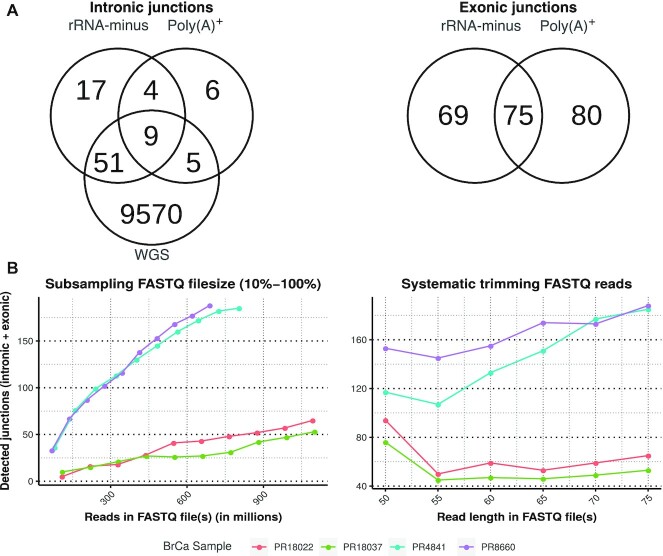
Overlap across sequencing types and library size influence. (**A**) Overlap of cumulative interchromosomal junctions of 7 WGS PCa samples rRNA-minus and poly(A)^+^ RNA-seq (PCa-LINES dataset). Overlap in only intronic junctions representing genomic breakpoints (left) and only exonic splice junctions (right). Of the 69 exonic junctions only found in rRNA-minus RNA-seq, 40 were detected in the matching poly(A)^+^ but did not pass filtering. Of the 80 poly(A)^+^-only exonic junctions, 58 were found in rRNA-minus but did not pass filtering. Not passing filtering mostly occurred because of insufficient discordant reads. (**B**) The number of predicted junctions as function of sequencing depth (left) and read-length (right) reduction. BrCa samples were selected for high sequencing depth (PR18022 and PR18037) or a high number of junctions (PR4841 and PR8660). *Left:* The number of predicted junctions per sequencing depth (10–100%) with the full read-length (2 × 75 bp). Reducing the sequencing depth, also for samples with a high sequencing depth, reduces the number of detected junctions. Sample PR4841 reaches a plateau. *Right:* Each data point represents the number of predicted junctions per given read length, at full sequencing depth. Truncating sequencing-reads results in a lower number of predicted junctions. However, below 55 nucleotides the number of detected junctions increases.

Results from 7 PCa samples with matching rRNA-minus and poly(A)^+^ RNA-seq (PCa-LINES dataset) were compared (Fig. 3A). Contrary to our initial hypothesis, the poly(A)^+^ results also revealed intronic junctions, representing genomic breakpoints. Still, rRNA-minus data identified (3.4×) more intronic junctions as compared to poly(A)^+^ RNA-seq. The intronic junctions identified in poly(A)^+^ often had lower read counts or were located in untranslated region terminal exons as in-exon located genomic breakpoints (Supplementary Fig. S3). Terminal exons are known for their relative large size as they are ∼6–7 times larger than internal exons [32]. The number of exonic junctions, thus predicted mRNA fusions, was nearly identical for rRNA-minus and poly(A)^+^ RNA-seq (144 vs 155).

### Comparison of RNA-seq with DNA-seq data

Within the PCa-LINES dataset, the number of WGS-identified genomic breakpoints vastly outnumbered those extracted from the rRNA-minus RNA-seq (6.8%), indicating that only a fraction of the genomic rearrangements is expressed at a level to be detected by rRNA-minus RNA-seq. Both intronic and exonic junctions from both rRNA-minus and poly(A)^+^ data co-located near WGS-detected genomic breakpoints (Supplementary Fig. S4), confirming their validity.

Four BrCa RNA-seq samples from the BASIS dataset [25, 26] were used to assess the influence of sequencing coverage and read length. Systematically truncating the reads showed that the number of detected junctions decreased as sequencing reads became shorter (Fig. 3B). From a read length <55 nt, the number of detected junctions increased. This was due to an overall increase in misalignments that do not resemble actual evidence of genomic rearrangements, indicating that a minimum length of 55 bp is needed for accurate detection. Irrespective of the number of genomic breakpoints present within a sample as determined by WGS, an increase in overall sequencing depth is positively correlated with an increase in detected junctions (Fig. 3B).

**Figure 4:**
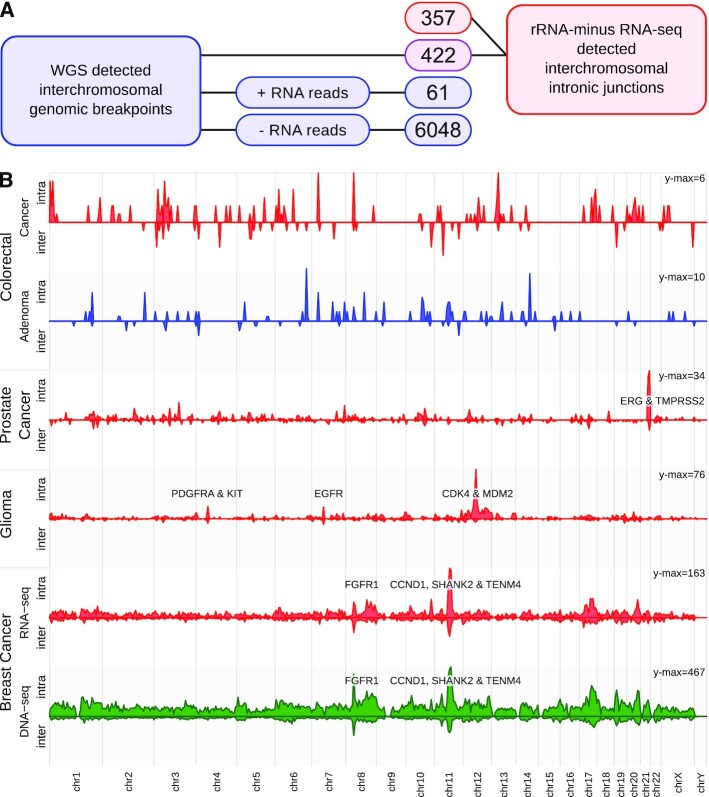
Integration RNA-seq analysis and WGS in BrCa. (**A**) Number of detected genomic breakpoints per subgroup in WGS and rRNA-minus RNA-seq data of 207 matching BrCa samples. Outlines in blue indicate presence only in WGS data, in red only in RNA-seq data, and in purple in both. To avoid artefacts from RNA post-processing such as circRNAs and read-throughs, only interchromosomal entries were interrogated. Of the interchromosomal WGS breakpoints, 6,048 did not have sufficient discordant reads in the RNA-seq data. Of 61 genomic breakpoints, the threshold of sufficient discordant RNA-seq reads was exceeded, but it was not detected by Dr. Disco or did not pass filtering. A total of 422 breakpoints were detected in both the assays and 357 RNA-seq–detected breakpoints did not match a WGS entry. (**B**) Chromosome plot representing the binned density of inter- and intrachromosomal intronic junctions. For the BrCa samples, Dr. Disco RNA-seq analysis (red) and WGS breakpoints (green) are depicted. The number of RNA-seq genomic breakpoints in the colorectal cancer and adenoma datasets is low and no recurrent breakpoints have been identified yet. The number of genomic breakpoints in colorectal adenomas was lower than in colorectal cancer. The observed peaks in colorectal cancer originated from multiple, sample-specific junctions (Supplementary Fig. S10).

All 207 WGS and rRNA-minus RNA-seq matching samples from the BASIS cohort [25, 26] were used to compare interchromosomal junctions. WGS identified a total of 6,531 interchromosomal genomic breakpoints, of which 422 (6.5%) were found in both assays (Fig. 4A), a similar percentage as in PCa-LINES. Dr. Disco detected 357 unique genomic breakpoints that were only present within the RNA-seq data, of which 100 were identified within only 8 BrCa samples that also had an overall high number of WGS-detected genomic breakpoints (Supplementary Fig. S5). The density of WGS- and rRNA-minus–detected junctions within chromosomal bins was highly similar (Pearson correlation: *r* = 0.72, Fig. 4B, Supplementary Figs S6–S8), with prominent focal peaks near the genomic loci of *CCND1*, *SHANK2*, and *FGFR1*.

### Pan-cancer analysis

**Figure 5:**
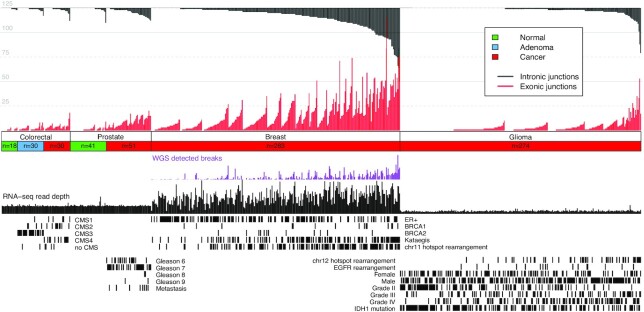
Pan-cancer results summary. Intronic and exonic junctions are given per sample for the NGS-ProToCol, BASIS, and CGGA datasets with their associated clinical parameters. For the colon samples, the predicted CMS classes are provided; for the prostate cancer samples, the Gleason grade and metastatic progression are provided; for the breast cancer samples, the ER, *BRCA1*, *BRCA2*, kataegis, and Dr. Disco–detected chr11-hot spot status are provided; and for the glioma samples, the grading, recurrence, *IDH1* mutation status, sex, and the Dr. Disco–detected *EGFR* and chr12 hot spot status are provided.

We analysed the results of the algorithm on rRNA-minus RNA-seq data (n = 651) from different malignant tissue types (Figs 4B and 5): the BASIS, NGS-ProToCol colon and prostate, and CGGA datasets (Table 1).

Intronic and exonic junctions were identified in each dataset. The different malignant tissue types showed distinct regions enriched with intronic and exonic junctions (Fig. 4B). Known prominent events include *TMPRSS2-ERG* in PCa, *EGFR*, *CDK4*, and *MDM2* in glioma, and *CCND1* in BrCa. The breakpoints per sample and associated clinical parameters are provided in Fig. 5. The lowest mean number of genomic breakpoints per tissue type was found in normal adjacent samples (colon = 0.5; prostate = 0.9) followed by colorectal adenoma (1.1) (Supplementary Figs S9 and S10). In 2 adjacent normal-looking prostate samples, intronic and exonic junctions were found that were exactly identical to junctions in their matching malignant sample. These adjacent normal-looking tissue samples were most likely contaminated with cancer cells (Supplementary Fig. S9B). Of the different malignant tissue types, colorectal cancer samples were characterized by the lowest mean number of junctions (1.1) followed by combined low- and high-grade glioma (2.1) (Supplementary Fig. S11). Conversely, PCa (4.3) and BrCa (9.3) were characterized by relatively high numbers of genomic breakpoints per sample. These mean numbers were not normalized for sequence depth because results are also influenced by dataset-specific differences in read length, stranding, RNA quality, and library preparation. Therefore, comparison of these mean numbers of junctions is confounded by these factors.

Associations between the number of detected intronic junctions per sample and clinical parameters were investigated within datasets (Fig. 5). In BrCa, presence of kataegis (*P* = 1.9 × 10^−9^) was positively associated with the number of observed intronic junctions whereas estrogen receptor–positive (ER^+^) tumours were negatively associated (*P* = 0.9 × 10^−3^) with the number of intronic junctions. In glioma, tumour grade IV is positively associated with the number of intronic junctions per sample (*P* = 1.1 × 10^−5^), whereas tumour grade II (*P* = 2.9 × 10^−8^) and presence of *IDH1* mutation (*P* = 0.8 × 10^−3^) were negatively associated. Within PCa no association was observed between the number of intronic junctions and the incidence of high Gleason grade (≥8; *P* = 0.08; n = 4/50) and metastasis (*P* = 0.16; n = 8/51). Within BrCa, the number of intronic junctions correlated positively with the number of WGS-detected genomic breakpoints (Spearman correlation: ρ = 0.71, *P* = 2.2 × 10^−16^, Supplementary Fig. S12). Because of the relative low number of junctions per sample combined with low number of colorectal cancer samples, further in-depth analysis on its recurrent events was not performed.

**Figure 6:**
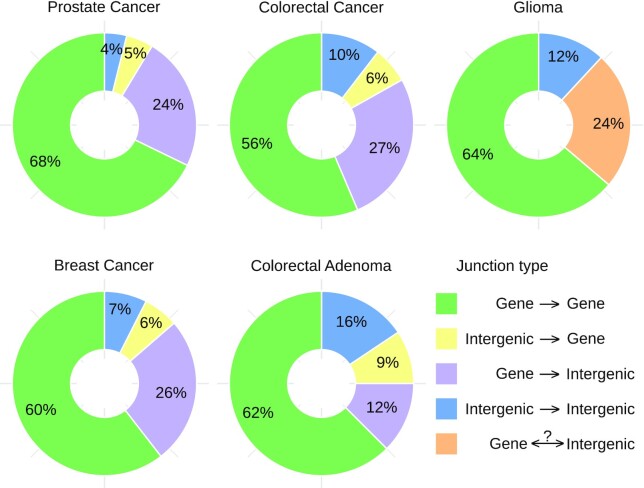
(Inter)genic junction status. Frequency of intronic and exonic junctions and their gene/intergenic status. Because the glioma dataset was sequenced unstranded, junctions with 1 intergenic side are grouped together. In all datasets, ∼3/8 of the junctions have ≥1 intergenic side. Inter- and intrachromosomal junctions were included, suspected circRNAs were discarded, and unlocalized and unplaced sequences (chrUn_...) and alternate loci (chr..._alt) spanning junctions were discarded. Intronic and exonic junctions corresponding to the same event were treated as a single entry.

In the CGGA, BASIS, and NGS-ProToCol datasets ∼65% of all intronic and exonic junctions have both sides located within an annotated gene (Fig. 6). Inversely, ∼35% of the junctions have ≥1 side located within an intergenic region, regions that are often dismissed a priori by classical fusion gene detection tools [19, 20]. We found transcripts that incorporated cryptic (unannotated) exons, both intergenic as intronic (including anti-sense). For instance, a BrCa sample harboured intergenic junctions in *SDC4* transcripts using 5 consecutive cryptic exons (Supplementary Fig. S13). In contrast, a PCa sample had an intergenic rearrangement lacking mRNA-level transcripts, thus only visible by the presence of pre-mRNA (Supplementary Fig. S14).

#### Genes associated with peaks in breakpoints

There were multiple, cancer type–specific hot spots of junctions located near known oncogenes (Fig. 4) such as *KIT*, *PDGFRA*, *EGFR*, *CDK4*, *MDM2* (glioma), *TMPRSS2*, *ERG* (PCa), *FGFR1*, and *CCDN1* (BrCa). Enrichment analysis was performed using HUGO symbols of genes recurrently hit per dataset, indicating that the pathway “Transcriptional misregulation in cancer [KEGG:05202]” was significantly more frequently hit (*P* = 1.6 × 10^−4^) within PCa due to *TMPRSS2*, *ERG*, *ETV1*, *H3FA3*, *SLC45A3*, and *ELK4*. Within BrCa, pathways ETF and E2F were significantly enriched (*P* = 6.75 × 10^−10^, *P* = 2.8 × 10^−6^) in ER^+^ BrCa and “Proteoglycans in cancer” in ER^−^ BrCa (*P* = 1.4 × 10^−5^). Genes that were recurrently hit in glioma were found more often in pathways “Rap1 signaling pathway” (*P* = 3.2 × 10^−4^), “Glioma” (*P* = 5.9 × 10^−3^), and “Ras Signaling” (*P* = 2.6 × 10^−3^) (Supplementary Table S2).

#### Large gene amplifications

Hot spot regions (20−30 Mb) enriched with RNA-seq−detected junctions were observed in the BrCa (chr11) and glioma (chr12) datasets. These hot spots differed from focal events such as *TMPRSS2-ERG* in the sense that they were larger, had no consistent fusion partners, and often contained multiple hot spot junctions per sample. If these hot spot region rearrangements are responsible for consistent changes at the transcriptional level, they may provide a selective advantage. In both the BrCa and glioma datasets, transcriptional effects of the hot spot rearrangements were investigated by performing differential gene expression analysis between samples with (BrCa: chr11, glioma: chr12) and without a hot spot rearrangement (BrCa: n = 122/283; glioma: n = 45/274, respectively).

**Figure 7:**
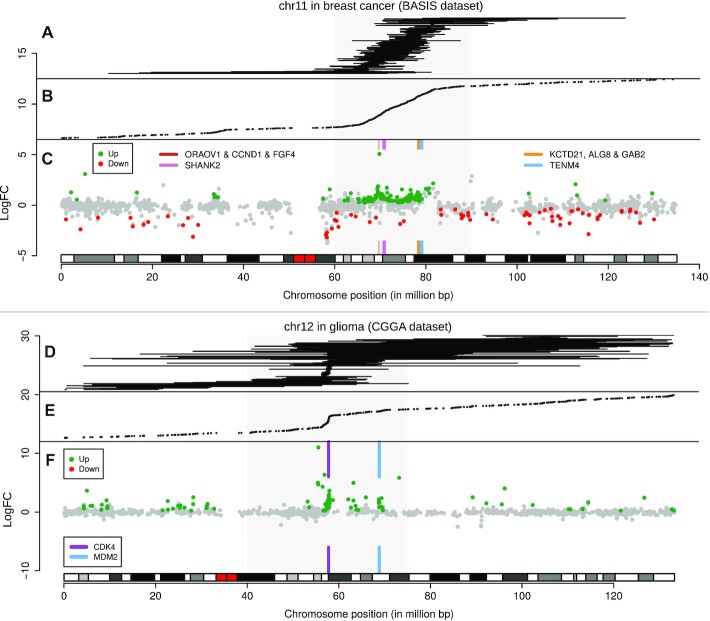
Differential gene expression in junction hot spot regions. (**A−C**) Overview of chr11 junctions, junction positions, and hot spot−associated differential gene expression in BrCa, using RNA-seq data only. (**A**) Intrachromosomal junctions not marked as putative circRNA, indicated by horizontal lines. (**B**) Junction end positions from intronic and exonic, inter- and intrachromosomal junctions not marked as putative circRNA. (**C**) Chromosomal differential expression plot for locus chr11:60,000,000**−**90,000,000 (grey square) with a q-value threshold of 0.001. Genes with the highest number of rearrangements, *SHANK2* and *TENM4*, are illustrated with coloured boxes. Peaks in logFC were observed surrounding *ORAOV1*, *CCND1* and *FGF4*, and surrounding *TENM4*. (**D−F**) Overview of chr12 junctions, junction positions, and hot spot−associated differential gene expression in glioma. (**D**) Intrachromosomal junctions not marked as putative circRNA are indicated with lines. (**E**) Junction end positions from intronic and exonic, inter- and intrachromosomal junctions not marked as putative circRNA. The junction enriched region chr12:40,000,000**−**75,000,000 is indicated with a grey square. (**F**) Chromosomal differential expression plot for locus chr12:40,000,000−75,000,000 with a q-value threshold of 0.01. Peaks in logFC from up-regulated genes are found near *CDK4* and *MDM2*.

BrCa samples having a chr11 hot spot rearrangement were characterized by a large stretch of significant up-regulated genes within the respective hot spot region (Fig 7A−C, Supplementary Fig. S15). The large genes *SHANK2* and *TENM4*, both located in the hot spot region, were the most frequently hit genes (25 and 13 samples, respectively), yet were not among the strongest up-regulated genes of the overall region. Instead, genes with a strong increase in logFC were *FGF4* and *CCND1*, the cluster *KCTD21*, *ALG8* and *GAB2*, and genes downstream of *TENM4*. Up-regulation of the overall region indicated amplifications of *CCND1* and/or the gene cluster, which is in concordance with previous reports [33]. We presume that selection of breakpoints near *SHANK2* is influenced by being adjacent to *CTTN*, a gene containing an enhancer often co-amplified with *CCND1* [34]. The high frequency of junctions in the relatively large, yet not heavily up-regulated *SHANK2* (785 kb) and *TENM4* (788 kb) suggests that they are “collateral damage” of the amplifications, a hypothesis that has been described in glioma [35]. This hypothesis is further supported by the lack of consistent fusion partners, consistency in acting as acceptor or donor, and the absence of a clear spike in cumulative breakpoints (Fig. 7A and B; Supplementary Table S3).

Glioma samples having a junction harbouring the chr12 hot spot region (Fig. 7D−F) were analysed similarly and also showed up-regulation of genes in the hot spot locus, with an increased logFC of *CDK4*, *MDM2*, and neighbouring genes. Both *CDK4* and *MDM2* are known to be hyper-amplified in glioblastoma [36], often by double minute chromosomes [37]. The junctions showed a sharp increase in close proximity of *CDK4* (Fig. 7D and E), likely indicating a common start of the amplification event. These breakpoints and up-regulated genes ceased just prior to *LRIG3*. Similarly, glioma samples harbouring rearrangements near the commonly hyper-amplified *EGFR* showed up-regulation of the surrounding locus (Supplementary Fig. S16).

Using RNA-seq data only, genomic rearrangements can be identified that can thereafter be used to reveal associated over-expression of oncogenes that have resulted from high-copy gene amplifications.

#### Chromothripsis

In VCaP, the q-arm of chr5 has been subjected to chromothripsis as revealed by 468 intrachromosomal WGS-detected breakpoints [24]. Seventeen intronic and exonic junctions were identified in rRNA-minus RNA-seq; thus evidence for chromothripsis events was identified at the (pre-)mRNA level (Supplementary Fig. S17). In 3 BrCa samples, high numbers of WGS-detected genomic breakpoints were identified on the q-arm of chr17. RNA-seq analyses revealed intronic and exonic junctions concordant with WGS data, which recurrently involved the genes *BCAS3*, *APPBP2*, *MED13*, *USP32*, and *VMP1* (Supplementary Fig. S18). Taken together, this demonstrates the possibility of observing chromothripsis-derived junctions in RNA-seq.

### TMPRSS2-ERG

**Figure 8:**
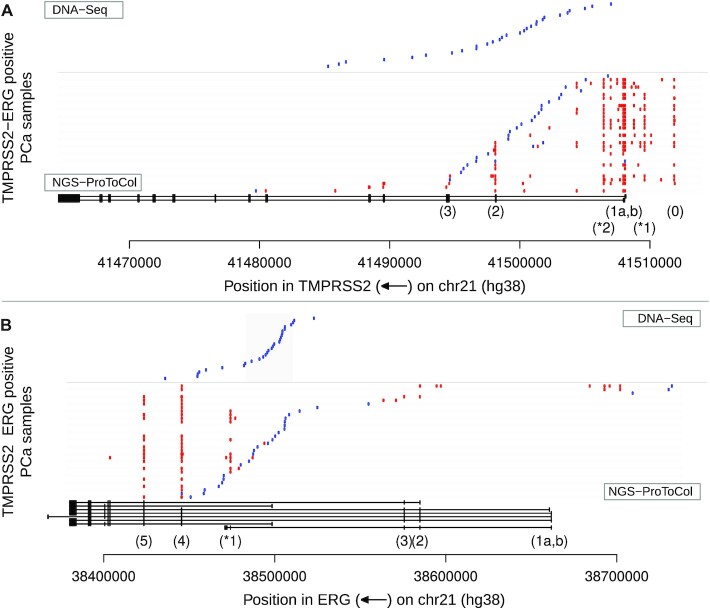
*TMPRSS2-ERG* junction map. *TMPRSS2* and *ERG* loci with endpoints of the junctions in NGS-ProToCol RNA-seq and non-matching targeted DNA-seq (Weier dataset). Gene structures are indicated at the bottom. Intronic junctions (representing genomic breakpoints) and genomic breakpoints (Weier dataset) are indicated in blue and exonic junctions in red. (**A**) For *TMPRSS2*, most breakpoints are detected after exon 1, up to exon 3. At mRNA level, apart from the first exons (1a and 1b), also exon 0 and exon 2 were commonly included in fusion transcripts. Two novel recurrent cryptic exons (*1 and *2) were common in fusion transcripts. (**B**) Three NGS-ProToCol samples (048, 05, and 075) have their genomic breakpoint before *ERG* and result in transcripts with additional, novel, intergenic cryptic exons.

From previous analyses it emerged that *TMPRSS2-ERG* was the most prominent focal event identified. Therefore, we leveraged NGS-ProToCol prostate tissue sample data to study this fusion in detail. *TMPRSS2-ERG* is a highly prevalent fusion gene in prostate cancer (∼50% of the diagnosed patients) [4], resulting in *TMPRSS2*-driven up-regulation of *ERG*. In 32 of the 51 samples Dr. Disco identified mRNA fusion transcripts of *TMPRSS2-ERG*, including genomic breakpoints in 27 of 32 samples (Fig. 8). These fusions were in concordance with high *ERG* expression in those samples exclusively. The detection rate for genomic breakpoints for this oncogenenic fusion gene is thus markedly higher than for the overall number of genomic breakpoints. The genomic breakpoint did not pass filtering in sample 072, was marked exonic in sample 027, and was merged with closely adjacent (<450 bp; insert size) exonic junctions in 3 samples (053, 050, and 065), indicating that breakpoint-spanning reads were present in all 32 *TMPRSS2-ERG*–positive RNA-seq samples.

Three samples had their *ERG*-flanking genomic breakpoint located in an intergenic region upstream to *ERG*’s first exon (Supplementary Figs S19 and S20). In 2 of these 3 samples, *TMPRSS2-ERG* fusion transcripts were identified containing cryptic intergenic exons (chr21:38,692,521–38,692,797 and chr21:38,701,593–38,701,947; hg38). In the same 2 samples, *ERG* had additional deletions, removing exon 2 (Supplementary Fig. S19).

The most abundant exonic junctions were T1-E4 and T1-E5 (Fig. 8, Supplementary Figs S20 and S21), which is in concordance with previous reports [38]. Genomic breakpoints were indeed located in hot spot regions within the first 2 introns of *TMPRSS2* and the last half of *ERG* intron 3 [39]. Subsequent analysis of shallow sequenced FFPE RNA-seq samples revealed *TMPRSS2-ERG* in 181 samples (Supplementary Figs S20 and S21) and confirmed this remarkable breakpoint preference region within *ERG* intron-3 more precisely.

Two novel exons in *TMPRSS2* were observed in both fusion and wild-type transcripts (Fig. 8). These cryptic exons were both expressed at a low level as they represented 3% of all *TMPRSS2-ERG* reads in samples having the splice variant. Furthermore, intergenic *TMPRSS2* exon-0 [40] was detected in fusion mRNA-transcripts within 18 of 32 *TMPRSS2-ERG*–positive samples.

One sample contained an exonic junction originating in *ERG* and spanning to *TMPRSS2* in which the gene order and included exons indicated that this *ERG-TMPRSS2* fusion was caused by a reciprocal translocation instead of the common 3-Mb deletion (Supplementary Fig. S22). Other *TMPRSS2*-related fusions were *TMPRSS2-RERE*, *SERINC5-TMPRSS2*, *TMPRSS2-TBX3*, *TMPRSS2-PADI4*, *MGA-TMPRSS2*, and *TMPRSS2-CATSPER2* (Supplementary Table S4).

#### VCaP

PCa cell line VCaP has been subjected to intensive research, revealing that it is *TMPRSS2-ERG* positive and contains 2 additional related genomic rearrangements (breakpoint A and breakpoint B) [24, 39]. *TMPRSS2-ERG* in VCaP was analysed using both rRNA-minus and poly(A)^+^ RNA-seq data.

Poly(A)^+^ RNA-seq shows that only the first exon of *TMPRSS2* splices to *ERG*, even though the genomic breakpoint to *ERG* is located in the fifth intron (Supplementary Fig. S23A). The rRNA-minus data not only confirm this splice junction but also reveal all *TMPRSS2*- and *ERG*-spanning genomic breakpoints, concordant with the WGS results. Moreover, read stranding indicates that a region containing the fourth and fifth exon is inverted and that its breakpoint A is an inversion. Breakpoint B is an amplification, and the junction from *TMPRSS2* to *ERG* is again inverted such that *ERG* is transcribed in its original orientation. The junction from *TMPRSS2* to *ERG* deletes the genomic region containing *ERG*'s exons 2 and 3. Thus, only *TMPRSS2* exon 1 splices to *ERG* because exons 2 and 3 are deleted and exons 4 and 5 are inverted (Supplementary Fig. S23B). The small proportion of reads still present within the deleted *TMPRSS2* exons 2 and 3 in the rRNA-minus data originate from the non-fusion allele(s). The rRNA-minus RNA-seq data not only revealed both intronic and exonic junctions but also showed the complex downstream effects on transcription and splicing.

### CircRNA detection

Head-to-tail aligned reads (Supplementary Fig. S24) are marked as chimeric (discordant) by STAR and are used as input for our method. Such reads are not only observed in transcripts from genomic tandem duplications but also from circular mitochondrial DNA and circRNAs. Using the PCa-LINES rRNA-minus samples, we found that 88.6% of the junctions with a head-to-tail orientation were located exactly on exon-junctions corresponding to annotated circRNAs from circBase v31 (Supplementary Fig. S25).

## Discussion

RNA-seq is generally performed as poly(A)+ RNA-seq, and fusion gene detection algorithms are in particular focused on annotated exons or splice junctions. It has become common practice to sequence ribosome-depleted total RNA (rRNA-minus) [12], especially for partially degraded (FFPE) RNA samples. rRNA-minus RNA-seq is interesting because it also yields non-polyadenylated transcripts, including pre-mRNA–derived intronic sequences. As a result, there is more genomic coverage in rRNA-minus RNA-seq alignments compared to poly(A)^+^ RNA-seq (Supplementary Fig. S26), which provides more opportunity to reveal fusion transcripts and a broader understanding of the transcriptome. Because genomic breakpoints are often harboured within introns [6] and intergenic regions (Supplementary Fig. S27), we interrogated to what extent rRNA-minus RNA-seq can be used to reveal genomic breakpoints because this also captures intronic (pre-mRNA) reads. Addressing this required analysing the genome without regional restrictions.

Here, we confirm, by using Dr. Disco, that RNA-seq data can be used to reveal genomic breakpoints of expressed transcripts in an automated fashion. Detection was limited to ∼7% of WGS-detected breakpoints but markedly higher for the driver *TMPRSS2*-*ERG* fusion gene (85% detected; 100% presence). Because the algorithm was conservative in detecting mRNA fusions, it is likely that genomic breakpoints were missed and the actual percentage is somewhat higher. Conversely, estimation of this percentage implies that WGS results offer the ground truth, but these are also affected by noise, coverage, and filter cut-offs, indicating that this percentage is an approximation. The results commonly included intergenic junctions. For instance, 3 *TMPRSS2*-*ERG* fusions had their breakpoint located before *ERG*, supported by cryptic intergenic splice junctions and intergenic pre-mRNA coverage (Supplementary Fig. S19). Furthermore, intronic intergenic junctions in chromothripsis regions in 3 BrCa samples were in 14 of 18 cases validated by WGS junctions (Supplementary Fig. S18A).

That both Arriba and Dr. Disco make use of STAR and that Arriba finds a higher number of true-positive results indicates that improving the filtering is an important future step, but care must be taken not to compromise Dr. Disco’s true-positive ratio. The large search space combined with graph analysis was an effective solution as shown by providing a unique view on 1,275 transcriptomes. Whereas our initial hypothesis was that only rRNA-minus RNA-seq would reveal genomic breakpoints, this also accounts for poly(A)^+^. In poly(A)^+^ RNA genomic breakpoints are observed less frequently, with lower confidence, and often in long untranslated regions. The VCaP *TMPRSS2-ERG* analysis underlined the differences: on the basis of rRNA-minus RNA-seq, each genomic rearrangement with resulting splice variants and their strand as well as the order of events could be deduced, while both the poly(A)^+^ and WGS alone were insufficient.

CircRNAs are a relatively new group of non-polyadenylated transcripts with >90,000 different human circRNAs identified so far [41, 42]. The distinctive signature of proximate exonic head-to-tail junctions sets them apart from other junctions, except for small tandem duplications. A useful addition to the algorithm could be annotation of the junctions using a circRNA database such as circBase [41]. The proposed method is not specifically designed to identify circRNAs because it has stringent cut-off levels, merges splice variants into subgraphs, and requires >1 read. The number of circRNAs identified is therefore lower as compared with dedicated detection tools such as CIRI [43, 44].

The number of RNA-seq–detected intronic junctions representing genomic breakpoints varied widely between the 4 different cancer types (PCa, BrCa, colorectal cancer, and glioma). This variation is in line with the omics-reported number of structural variants—low in colorectal cancer [45] while high in BrCa [46, 47]—but these differences are confounded by the influence of sequencing depth, length, and library preparation, which vary per dataset.

While only a fraction of all genomic rearrangements is transcribed, an even smaller fraction is causal for fusion genes, which are currently of high interest in RNA research. Transcribed genomic breakpoints more often involve driver events than non-transcribed genomic breakpoints, as seen with *TMPRSS2-ERG*. Known exceptions that can be considered driver events include promoter and enhancer rearrangements such as known for *AR* and *FOXP1* [48], but also tumour suppressor gene deletions [34, 49]. The reasoning that RNA-seq is not a replacement for WGS is valid. However, we show that by also looking at intronic and intergenic regions, more cancer-specific transcripts are identified and context is provided than being restricted to classic mRNA fusion genes only.

Although WGS depth surpasses 40× coverage, Dr. Disco showed that 26% and 48% of all RNA-seq intronic junctions in PCa and BrCa, respectively, were not identified by WGS. While these will contain false-positive results for sure, 100 of the 357 were found in 8 of the 207 samples all characterized by high number of genomic breakpoints. We suspect that this discrepancy is partially due to actual genomic breakpoints missed by WGS because of the following factors: high RNA-seq coverage of highly expressed genes (up to 1000×), clonality because this difference was in particular high for a small subset of samples, low local coverage in WGS, and selection criteria in software such as cut-offs and read mapping rulings. In the PCMM-FFPE dataset, samples with low insert sizes or short read lengths often resulted in insufficient split-reads whilst resulting in many false positive read-pairs in full transcriptome analysis. However, FFPE data could still be used effectively in identifying the targeted, highly expressed, *TMPRSS2-ERG* fusion events.

The large number of PCa samples allowed *TMPRSS2-ERG* to be analysed in-depth, revealing additional cryptic and intergenic exons including *TMPRSS2* exon 0 [40], a detailed map of the genomic breakpoints, genomic breakpoints located before *ERG* that combined with cryptic intergenic exons co-exist with exon 2 deletions, and a tumour that harbours *TMPRSS2-ERG* that originated from a reciprocal translocation rather than a deletion. In VCaP, stranded RNA-seq provided an advantage in deciphering the chronological order of complex genomic events. Moreover, this underlined the importance of automatic resolution of complex genomic rearrangements or poly-fusions. The current implementation does not offer such top-level integration for poly-fusions, while techniques exist with this purpose in mind [7, 50]. Integration of such techniques prompts future work. The current algorithm uses discordant reads exclusively. It would be interesting to investigate the added value of extending the detection with regions enriched with concordant opposite stranded reads, to strengthen detection of junctions having insufficient coverage of discordant reads.

In both BrCa and glioma, RNA-seq data alone revealed hot spot regions of junctions with subsequent up-regulation of known amplified oncogenes and neighbouring genes within these regions. The inconsistent transcriptional direction of the junctions combined with the lack of consistent acceptor/donor genes provides additional context that distinguishes these events from focal fusion genes. In BrCa, this combined analysis indicated that the events are related to *CCND1* amplifications, despite the frequent events in sizeable genes *TEMN4* and *SHANK2* of which their fusion transcripts are not driving cancer.

Chromothripsis-derived junctions matching WGS-detected genomic breakpoints were present at the RNA level, in VCaP and 3 BrCa samples. As with most genomic rearrangements, the majority of the chromothripsis rearrangements were not detected on the RNA level. Solely on the basis of RNA-seq data, it will be difficult to prove the presence of chromothripsis because not all parameters that define this specific process can readily be extracted (e.g., copy-number variations, short insertions, loss of heterozygosity) [51, 52]. However, potential indicators for oncogenic chromothripsis events can be present in RNA.

While our preliminary aim was to study to which extent genomic breakpoints are present in RNA, we were surprised by how common both intergenic events and cryptic exons are. That ∼35% of the junctions in rRNA-minus datasets were full or partial intergenic events is an under-representation because, as for example with *TMPRSS2-ERG* exon-0, intergenic splice variants are merged with gene-spanning splice variants and will be considered in-gene as-whole. Intergenic mRNA fusions are characterized by incorporation of (typically multiple) cryptic exons. But cryptic exons are not limited to intergenic events, as we reported that cryptic exons in fusion transcripts transcribed in the anti-sense direction of a gene are common.

Cryptic exons are of importance because they may encode nonsense proteins with completely novel neo-antigens that are more divergent than point mutation–based neo-antigens and could therefore be more immunogenic [53]. We want to emphasize the importance of fusions beyond those incorporating annotated exons because we show that they can be transcribed into stable mRNA, thereafter be translated into protein, and potentially be oncogenic and/or immunogenic. This, deciphering the consequence of rearrangements, annotation of cryptic exons, and their coding potential for nonsense protein sequences is relevant for therapeutic interventions using tumour-specific antigens [54].

## Conclusion

Facilitated by Dr. Disco, we set out to extract both intronic and exonic junctions from comprehensive rRNA-minus RNA-seq datasets and identified novel genomic breakpoints, circRNAs, novel gene and intergenic fusions, cryptic exons, and chromothripsis events and were able to link expressed rearrangements to transcriptional outcome. Discovering both genomic breakpoints and exonic junctions from only RNA-seq data requires an analysis strategy keeping these 2 levels of information separated. The number of breakpoints detected is limited to ∼7% because most are not within expressed regions. These results indicate that this analysis is not a replacement for WGS, but performing analysis like this will result in considerably more cancer-specific transcripts than interrogating classical fusion genes only. This holds in particular for rRNA-minus RNA-seq, which harbours more intronic reads. Furthermore, combined WGS and RNA analysis showed that RNA can function as informative supplement to WGS analysis because of stranding, expression, and resolution of the fusion gene structure(s). rRNA-minus RNA-seq provides more unique and complete information on non-polyadenylated and aberrant transcripts and, if the pre-mRNA is sequenced, the genomic breakpoints that underlie transcriptional changes.

Thus, RNA-seq data can reveal genomic breakpoints, (cryptic and/or intergenic) splicing, and gene expression information, which together can reveal consequences and their selective advantage for cancer development and progression and be a useful supplement to DNA-seq.

## Methods

### Sequencing and datasets

The sequencing datasets used are given in Table 1. For NGS-ProToCol and the rRNA-minus RNA-seq of PCa-LINES, RNA was extracted using RNA-Bee (Campro Scientific, Berlin, Germany), and the library prepared for RNA-seq used the NEBNext Ultra Directional RNA Library Prep Kit for Illumina with rRNA reduction (New England BioLabs, Ipswich, Massachusetts, United States of America). The sample preparation was performed according to the protocol “NEBNext Ultra Directional RNA Library Prep Kit for Illumina” (NEB, Cat. No. E7420S/L and E6310S/L/X). Briefly, rRNA was reduced using the RNase H-based method. Then, fragmentation of the rRNA-reduced RNA and a complementary DNA synthesis was performed. This was used for ligation with the sequencing adapters and PCR amplification of the resulting product. The quality and yield after sample preparation were measured with the Fragment Analyzer (Advanced Analytical). Clustering and DNA sequencing using the Illumina cBot and HiSeq 2500 was performed according to manufacturer’s protocols (ServiceXS, Leiden, The Netherlands). A concentration of 16.0 pM of DNA was used as input. HiSeq control software HCS (v2.2.58) was used. Image analysis, base calling, and quality check were performed with the Illumina data analysis pipeline RTA (v1.18.64) and Bcl2fastq (v2.17). The 126-bp stranded Illumina HiSeq 2500 paired-end reads have a peak in fragment size of 300–600 bp and the samples have a mean depth of 70 million paired-end reads.

Of the PCa-LINES samples, each sample was WGS DNA sequenced and processed using the Complete Genomics platform [24, 57] (CompleteGenomics, San Jose, California, United States of America). The matching poly(A)+ RNA-seq samples were taken from the TraIT-Cell Line Use Case study [55, 58]. rRNA-minus RNA-seq sample G-110 was not sequenced within PCa-LINEs but sequenced in NGS-ProToCol as sample 7046-004-052.

In the BASIS RNA-seq dataset [26], total RNA was extracted and cleaned from abundant RNAs such as rRNA and transfer RNA as described elsewhere [27]. The BASIS DNA-seq data preparation and analysis is described elsewhere [25] and coordinates were converted to hg38 using pyliftover (v0.4) where needed.

The detection of genomic breakpoints from additional *TMPRSS2-ERG* fusions determined by targeted DNA-seq was described elsewhere [39], and genomic coordinates were obtained from this study accordingly. Genomic breakpoints of *TMPRSS2-ERG* and chromothripsis on chr5 in VCaP were described elsewhere [24, 39]. Predicted CMS classes for NGS-ProToCol colon samples were described elsewhere [22]. CGGA metadata were described elsewhere [28].

### Computational analysis

RNA-seq data were aligned with STAR [59] (v2.4.2) using fusion settings and hg38 as reference genome. More details are given in the Supplementary Methods. Dr. Disco (v0.17.8, git commit 2a9ff32950b71029b124ff4d16544b2953c57dbe) was used for analysis. Arriba [31] (v2.1.0, git commit 3492d2c28917fe6c9320b1caab73afbb93f7bfbf) was used for evaluation analysis. For Supplementary Fig. S26, we designed and used our free software package to generate Lorenz and coverage plots and statistics (bam-lorenz-coverage, v2.3.0). Processed bam files used to estimate general genome coverage statistics were obtained from EGAS00000000052 [56]. Pathway enrichment was performed with g:Profiler web (https://biit.cs.ut.ee/gprofiler/gost [60]), using gene identifiers as non-ordered query. For differential expression analysis, the annotation of the results of Dr. Disco, and further integration with gene sets for determining intergenic status, Ensembl 89 was used.

Plots were made with base R (3.6.3), ggplot2 (3.3.5), plotrix (3.8.1), and circlize (0.4.13), and illustrations, with Inkscape (0.92.4). Differential gene expression analysis was performed using edgeR (3.28.1) [61]. Associations between the frequency of breakpoints per sample and clinical parameters were tested using the Mann-Whitney *U* test in R.

For the Venn diagrams describing overlap across intronic, exonic, and WGS junctions (Fig. 3A), both sides of the junctions must be within 40 genomic nucleotides in proximity to be considered a match. Head-to-tail junctions and junctions to alternate loci were excluded. For the comparison of BrCa WGS and rRNA-minus results (Fig. 4), interchromosomal entries were compared to avoid an unfair comparison due to (i) small WGS indels detected on the basis of non-split reads and (ii) transcripts unrelated to genomic rearrangements such as read-throughs or circRNAs.

Chromosomal differential expression plots (Fig. 8) were made using base R. For a given locus and q-value threshold, a cohort is separated in a mutant and wild-type group by having 1 or more intronic or exonic junctions within the given locus. Differential expression analysis is performed across these groups using edgeR. Every gene located on the chromosome on which the locus is located is plotted with its genomic center as defined by Ensembl 89 on the x-axis and with edgeR’s logFC on the y-axis. A gene that is up-regulated in the mutant group has a positive logFC change, and a gene that is down-regulated, a negative logFC. When the gene is not significantly differentially expressed across the wild-type and mutant group (q-value below predetermined threshold) the gene will be coloured grey. If the difference is significant, it will be coloured green (up) or red (down).

Snapshots of discordant alignments were made in IGV (2.8.0) using the split view and with "Color alignments by" set to "read group."

## Data Availability

Supporting data, including reference data, final results tables, and chimeric alignments, and an archival copy of the code are available in the GigaDB database [62]. The results of the evaluation on the ENCODE MCF-7 dataset are given as Supplementary Table S1 [62].

## Availability of Source Code and Requirements

- Project name: Dr. Disco
- Project home page: https://github.com/yhoogstrate/dr-disco
- bio.tools: https://bio.tools/dr_disco
- RRID:SCR_021739
- Operating system(s): GNU/Linux
- Programming language: Python
- Other requirements: STAR (aligner)
- License: GNU GPL 3.0
- Project name: bam-lorenz-coverage
- Project home page: https://github.com/yhoogstrate/bam-lorenz-coverage
- bio.tools: https://bio.tools/bam-lorenz-coverage
- RRID: SCR_021837
- Operating system(s): GNU/Linux
- Programming language: Python
- Other requirements: Python libraries: numpy, pysam, tqdm, matplotlib & click

## Additional Files

**SupplementaryDr**. **Disco Technical Specification**

**Supplementary Figure S1**. Exonic and intronic junctions

**Supplementary Figure S2**. Snapshot of fusion using cryptic exons in ENCODE MCF-7 dataset

**Supplementary Figure S3**. IGV Screenshot of in-exon genomic breakpoint

**Supplementary Figure S4**. Junctions in PCa-LINES dataset

**Supplementary Figure S5**. rRNA-minus RNA-seq and WGS data intersection BASIS dataset

**Supplementary Figure S6**. Correlation binned density WGS breakpoints and rRNA-minus junctions

**Supplementary Figure S7**. Intrachromosomal junctions in BASIS dataset

**Supplementary Figure S8**. Intrerchromosomal junctions in BASIS dataset

**Supplementary Figure S9**. Intrachromosomal junctions in NGS-ProToCol prostate dataset

**Supplementary Figure S10**. Intrachromosomal junctions in NGS-ProToCol colon dataset

**Supplementary Figure S11**. Intrachromosomal junctions in CGGA dataset

**Supplementary Figure S12**. Correlation RNA-seq depth, WGS breakpoints, and RNA-seq junctions in BASIS dataset

**Supplementary Figure S13**. Snapshot cryptic fusion in BrCa sample PR9608a

**Supplementary Figure S14**. Snapshot of intergenic breakpoints in PCa sample 7046-004-134

**Supplementary Figure S15**. Chromosomal differential expression plot of chr11 in BASIS dataset

**Supplementary Figure S16**. Chromosomal differential expression plot of *EGFR* in CGGA dataset

**Supplementary Figure S17**. Chromothripsis on chr5q in VCaP

**Supplementary Figure S18**. Catastrophic events on chr17 in BASIS dataset

**Supplementary Figure S19**. Snapshot of intergenic breakpoints in *TMPRSS2-ERG*

**Supplementary Figure S20**. Detailed overview of junctions in *ERG*

**Supplementary Figure S21**. Detailed overview of junctions in *TMPRSS2*

**Supplementary Figure S22**. Schematic representation of *TMPRSS2-ERG* as reciprocal translocation

**Supplementary Figure S23**. *TMPRSS2-ERG* in VCaP

**Supplementary Figure S24**. Schematic overview discordant read orientations

**Supplementary Figure S25**. Overlap head-to-tail junctions and circBase in PCa-LINES dataset

**Supplementary Figure S26**. Lorenz and coverage plots of rRNA-minus, poly(A)+, and WGS data

**Supplementary Figure S27**. (Inter)genic events in WGS

**Supplementary Methods**

**SupplementaryTable S1**.Results SRR534293

**Supplementary Table S2**.gProfiler

**SupplementaryTableS3**.SHANK2 and TENM4 related junctions

**Supplementary Table S4**.TMPRSS2-ERG and related junctions

## Abbreviations

bp: base pairs; BrCa: breast cancer; circRNA: circular RNA; edgeR: empirical analysis of DGE in R; ER^+^: estrogen receptor–positive; FFPE: formalin-fixed paraffin-embedded; IGV: Integrative Genomics Viewer; logFC: logarithmic fold change; Mb: megabase pairs; mRNA: messenger RNA (5′ capped, polyadenylated, and spliced); PCa: prostate cancer; poly(A)^+^: polyadenylated; pre-mRNA: RNA that is actively being transcribed by polymerase (not polyadenylated); rRNA-minus RNA-seq: RNA-seq prepared such that there is no specific positive selection for poly(A)-tails while reducing the amount of ribosomal RNA; poly(A)^+^ RNA-seq: RNA-seq prepared with a positive selection for poly(A)-tails; STAR: Spliced Transcripts Alignment to a Reference; WGS: whole-genome sequencing.

## Ethical Approval

For the CTMM NGS-ProToCol study (NGS-ProToCol, Next Generation Sequencing from Prostate to Colorectal Cancer - Center for Translational Molecular Medicine (2014-2015); https://www.lygature.org/ctmm-portfolio), 51 prostate cancers from the Erasmus MC were snap-frozen and stored in liquid nitrogen as previously described [63]. Use of the samples for research purposes was approved by the Erasmus MC Medical Ethics Committee according to the Medical Research Involving Human Subjects Act (MEC-2004-261; MEC-2010-176).

## Competing Interests

The authors declare that they have no competing interests.

## Funding

This study was performed within the framework of the CTMM (Center for Translational Molecular Medicine) research program; NGS-ProToCol (grant 03O-402); PCMM (grant 03O-203-1); Translational Research IT (TraIT); the Complete Genomics Inc. grant (EMC GL 083111); the FP7 Marie Curie Initial Training Network PRO-NEST (grant No. 238278) and Support for the Cancer Computational Biology Center was provided by the Daniel den Hoed Foundation. Funding for open access charge: NGS-ProToCol (grant 03O-402).

## Authors' Contributions

Y.H. and G.W.J. designed most of the experiments. Y.H. carried out most of the experiments and analysis and wrote the manuscript and software. M.A.K., R.B., J.v.R., H.J.G.v.d.W., R.H., A.P.S., and G.W.J. contributed to the methodology. N.D., D.S., D.v.d.M., F.P., C.H.B., G.J.L.H.v.L., M.S., J.W.M.M., W.v.W., B.J., E.C., M.d.J., G.A.M., E.v.d.B, A.S.B, R.H., R.J.A.F., and G.W.J. acquired data. H.JG.v.d.W, J.v.R, S.v.L., C.R., R.B., M.S., A.P.S., and M.A.K. analysed and prepared data. P.J.F., R.B., M.S., G.W.J., B.J., R.J.A.F., and P.J.v.d.S. made large contributions to writing the manuscript. G.J., A.P.S., P.J.F., C.H.B., R.J.A.F., and P.J.v.d.S. acquired funding.

## Supplementary Material

giab080_GIGA-D-21-00236_Original_Submission

giab080_GIGA-D-21-00236_Revision_1

giab080_GIGA-D-21-00236_Revision_2

giab080_Response_to_Reviewer_Comments_Original_Submission

giab080_Response_to_Reviewer_Comments_Revision_1

giab080_Reviewer_1_Report_Original_Submission Wencke Walter, Ph.D. -- 8/30/2021 Reviewed

giab080_Reviewer_1_Report_Revision_1 Wencke Walter, Ph.D. -- 10/11/2021 Reviewed

giab080_Reviewer_2_Report_Original_Submission Daniel Nicorici -- 9/7/2021 Reviewed

giab080_Reviewer_3_Report_Original_Submission Bo Li, Ph.D. -- 9/12/2021 Reviewed

giab080_Supplemental_Files
