# Supplementary material for: Fusion transcripts and their genomic breakpoints in polyadenylated and ribosomal RNA–minus RNA sequencing data
Source: Gigascience. 2021 Dec 9;10(12):giab080. doi: 10.1093/gigascience/giab080 (PMC8673554; doi:10.1093/gigascience/giab080)
Supplement: giab080_GIGA-D-21-00236_Revision_1 [file giab080_giga-d-21-00236_revision_1.pdf]

GigaScience

Fusion transcripts and their genomic breakpoints in poly(A)+ and rRNA-minus RNA sequencing data

--Manuscript Draft--

**Manuscript Number:**	GIGA-D-21-00236R1
**Full Title:**	Fusion transcripts and their genomic breakpoints in poly(A)+ and rRNA-minus RNA sequencing data
**Article Type:**	Technical Note
**Funding Information:**	center for translational molecular medicine ([grant 03O-402) — Prof. Guido W. Jenster<br>center for translational molecular medicine (grant 03O-203-1) — Prof. Guido W. Jenster<br>complete genomics (EMC GL 083111) — Prof. Guido W. Jenster<br>pro-nest (grant number 238278) — Prof. Guido W. Jenster
**Abstract:**	Background: Fusion genes are typically identified by RNA-seq without elucidating the causal genomic breakpoints. However, non poly(A)-enriched RNA-seq contains large proportions of intronic reads spanning also genomic breakpoints.<br>Results: We have developed an algorithm, Dr. Disco, that searches for fusion transcripts by taking an entire reference genome into account as search space. This includes exons but also introns, intergenic regions and sequences that do not meet splice junction motifs. Using 1,275 RNA-seq samples, we investigated to what extent genomic breakpoints can be extracted from RNA-seq data and their implications regarding poly(A)+ and rRNA-minus RNA-seq data. Comparison with WGS data revealed that most genomic breakpoints are not, or minimally, transcribed while, in contrast, the genomic breakpoints of all 32 TMPRSS2-ERG positive tumors were present at RNA level. We also revealed tumors in which the ERG breakpoint was located before ERG, which co-existed with additional deletions and mRNA that incorporated intergenic cryptic exons. In breast cancer we identified rearrangement hotspots near CCND1 and in glioma near CDK4 and MDM2 and could directly associate this with increased expression. Furthermore, in all datasets we find fusions to intergenic regions, often spanning multiple cryptic exons that potentially encode neo-antigens. Thus, fusion transcripts other than classical gene-to-gene fusions are prominently present and can be identified using RNA-seq.<br>Conclusion: By using the full potential of non poly(A)-enriched RNA-seq data, sophisticated analysis can reliably identify expressed genomic breakpoints and their transcriptional effects.
**Corresponding Author:**	Y Hoogstrate<br>Erasmus Medical Center: Erasmus MC<br>Rotterdam, NETHERLANDS
**Corresponding Author Secondary Information:**	
**Corresponding Author's Institution:**	Erasmus Medical Center: Erasmus MC
**Corresponding Author's Secondary Institution:**	
**First Author:**	Youri Hoogstrate
**First Author Secondary Information:**	
**Order of Authors:**	Youri Hoogstrate<br>Malgorzata A. Komor<br>René Böttcher<br>Job van Riet

Powered by Editorial Manager® and ProduXion Manager® from Aries Systems Corporation

	Harmen J.G. van de Werken
	Stef van Lieshout
	Ralf Hoffmann
	Evert van den Broek
	Anne S. Bolijn
	Natasja Dits
	Daoud Sie
	David van der Meer
	Floor Pepers
	Chris H. Bangma
	Geert J.L.H. van Leenders
	Marcel Smid
	Pim J. French
	John W.M. Martens
	Wilbert van Workum
	Peter J. van der Spek
	Bart Janssen
	Eric Caldenhoven
	Christian Rausch
	Mark de Jong
	Andrew P. Stubbs
	Gerrit A. Meijer
	Remond J.A. Fijneman
	Guido W. Jenster
**Order of Authors Secondary Information:**	
**Response to Reviewers:**	Rotterdam, October 07, 2021<br><br>Dear Hans Zauner,<br><br>Please find attached our re-revised manuscript "Fusion transcripts and their genomic breakpoints in poly(A)+ and rRNA-minus RNA sequencing data" by Youri Hoogstrate et al. which we would like to resubmit for publication in GigaScience.<br>We would like to thank the referees for critical assessment of manuscript. The issues that were raised are addressed pointwise following this letter. Furthermore, we have re-written the manuscript as Technical Note and therefore excluded the “Potential implications” section and included bio.tools and SciCrunch.org identifiers. We believe to have adequately addressed all issues raised by the referees and with these changes we hope our manuscript is suitable for publication in GigaScience.<br><br>Yours sincerely,<br><br>Youri Hoogstrate, PhD (on behalf of all co-authors)

Powered by Editorial Manager® and ProduXion Manager® from Aries Systems Corporation

Point-wise response reviewer comments
Reviewer #1:
Q: Can Dr. Disco be used for single sample analysis or is it preferentially used for bulk analysis?
A: Dr. Disco can be used for single sample analysis. We addressed this in the manuscript by adding:
“While only large datasets were analyzed in this study, the method is explicitly developed for single-sample analysis.”
Also the github landing page was updated accordingly.

Q: Please review the figure legend of Fig. S7A. Seems like there is a mix-up: "This figure is divided over 4 sub figures (6A-6D)."
A: This mix-up has been revised. Thanks.

Q: It is mentioned that the large search space requires a more stringent filtering. How does this reflect on the run time of the algorithm? Is the run time comparable to the other fusion callers?
A: Reviewer 1 asks whether the large search space and stringent filtering affect run time performance as compared to other fusion callers. The graph construction and deconvolution are the most time consuming part of the algorithm. Using the evaluation MCF-7 dataset, we observed that the performance of this module was 9 minutes and 48 and filtering 10 second(s). The integration part, in which all candidate entries are compared to share overlap and are annotated with corresponding gene names and sequence motifs from reference FASTA and GTF files, also took a considerable amount of time (5 minutes and 51 seconds). We compared this to Arriba because both post-process the output of STAR. According to the log files or Arriba, it took 5 minutes and 9 seconds to read the chimeric input and reference files. We therefore concluded that Dr. Disco took 949 seconds and Arriba 458 seconds, and Arriba was thus ~2x times faster. We have therefore added the following text to the manuscript:
“The time it took Dr. Disco to complete analysis after the STAR alignment was 949 seconds, 2.07 times slower compared with Arriba (458 seconds).”
And changed:
“In concordance with our expectations, the large search space required more conservative filtering.”
->
“This is in concordance with the expectation that analysing a larger search space requires more conservative filtering and takes more resources to complete.”

Q: It is mentioned that the number of identified true positives is lower for Dr. Disco. What was the overall accuracy of the method?
A: The results in the initial draft assessed only the cumulative number of true positives. To address this comment, we extended Figure 2 by providing the ratio-of-TP compared to the total calls. From this analysis, the conclusion did not change; Dr. Disco is stringent, but of the calls it makes, a high proportion is true positive. However, JAFFA’s TP ratio was better. Conversely, Arriba finds most fusions and thus most TP fusions, but with a considerably lower TP-ratio, often finding fusions involving rRNA genes. Nevertheless, this analysis has proven that more fusions are present within Chimeric STAR alignments than Dr. Disco finds and improvement of filters prompts future work. We have adjusted the results section and added the following discussion point:
“That both Arriba and Dr. Disco make use of STAR and that Arriba finds a higher number of true positives indicates that improving the filtering is an important future step, but care must be taken not to compromise Dr. Disco's true positive ratio.”

Q: How does the method compare to other fusion calling pipelines that rely on the chimeric junction information file of STAR (e.g. STAR-Fusion, arriba, etc)?
A: We have analyzed the results of STAR based detector Arriba and attempted the same with STAR-Fusion. STAR-Fusion crashed after 619 minutes with the following error: “died with ret 512 No such file or directory at /home/youri/.local/src/STAR-Fusion/PerlLib/Pipeliner.pm line 181.
Pipeliner::run(Pipeliner=HASH(0x562737a6dca8)) called at /home/youri/.local/src/STAR-Fusion/STAR-Fusion line 797”. STAR-Fusion was thereafter excluded from evaluation.
Although the TP-ratio of Arriba detected fusions was limited, it had an impressively

high sensitivity indicating that STAR can provide more detectable junctions than Dr. Disco (and other tools) did. We updated the results and added the following to the discussion:
“That both Arriba and Dr. Disco make use of STAR and that Arriba finds a higher number of true positives indicates that improving the filtering is an important future step, but care must be taken not to compromise Dr. Disco's true positive ratio.”

Q: The difference in the number of identified genomic breakpoints between RNA-Seq and DNA-Seq is addressed and it is reasoned that only a small fraction of genomic rearrangements is expressed. It is well known that not all genomic rearrangements generate a corresponding fusion transcript and hence the overlap should be considerably smaller. However, the high number of detected genomic breakpoints by WGS will also contain a high number of false positive calls due to the comparable low coverage of the WGS data and the high noise associated with structural variant calling. Hence, the ~7% might be an underestimation.
A: Reviewer 1 raises an interesting point by noting that there might be more than 7% of the breakpoints expressed because WGS results can be incomplete (false negatives) and noisy (false positives). We indeed suspect, as shown in figure S7A (junctions detected at chr11 in BrCa that were missed by WGS), that WGS results of the BrCa data are likely missing true genomic events. But the opposite may also be true, that this is an overestimation and WGS results were really stringent. In both directions, discussion regarding this issue is speculative as both assays are affected by ‘noise’ that we cannot proof unambiguously. We have therefore changed the discussion into:
“Here, we confirm by utilizing Dr. Disco, that RNA-seq data can be used to reveal genomic breakpoints of expressed transcripts in an automated fashion. Detection was limited to approximately 7% of WGS detected breakpoints but markedly higher for the driver TMPRSS2-ERG fusion gene (85% detected; 100% presence). As the algorithm was conservative in detecting mRNA fusions, it is likely that genomic breakpoints were missed and the actual percentage is somewhat higher. Conversely, estimation of this percentage implies that WGS results offer the ground truth but these are also affected by noise, coverage and filter cut-offs, indicating this percentage is an approximation.”

Q: "Dr. Disco detected 357 unique genomic breakpoints (45.8%)..." It is not quite clear to which result the percentage refers to. Please rephrase.
A: We have rephrased the sentence into: “Dr. Disco detected 357 unique genomic breakpoints which were only…”

Q: It is indicated that the number of identified genomic breakpoints differs significantly between the various cancer types. It is obvious from Fig.5 that the read depth differs significantly between the various data sets, as briefly addressed in the discussion. Thus, is there a correlation between the number of identified breakpoints and the read depth that might confound the result? Similar to the correlation analysis of Fig S12.
A: Reviewer 1 asks whether there is a correlation between the number of breakpoints and the read depth. This is indeed the case as presented in Figure 3B, in which systematically truncating the read depth of four samples resulted in an associated reduction of detected junctions. Therefore, reviewer 1 raises a valid point by noting that this confounds interpreting the comparison of average junctions per sample, per dataset which differ in coverage. Other factors such as read length and library preparation also contribute to this problem and therefore make it, unfortunately, unfeasible to perform a correction on this. In the initial manuscript, we have addressed this issue by stating:
“These average numbers were not normalized for sequence depth as they are also confounded by differences in read length, stranding, RNA quality and library preparation.”.
As this is not sufficiently detailed, we therefore have added the following:
“These average numbers were not normalized for sequence depth as results are also influenced by dataset specific differences in read length, stranding, RNA quality and library preparation. Therefore, comparison of these average numbers of junctions is confounded by these factors.”
We have also changed the following sentence:
“This variation is in line with the omics-reported number of structural variants; low in colorectal cancer [ref] while high in breast cancer [ref,ref], but is influenced by sequencing depth, length and library preparation which vary per dataset.”
into

	"This variation is in line with the omics-reported number of structural variants; low in colorectal cancer [ref] while high in breast cancer [ref,ref], but these differences are confounded by the influence of sequencing depth, length and library preparation which vary per dataset."<br><br>Q: Is there any support or validation for the identified junctions with at least one side located within an intergenic region (Fig. 6)?<br>A: Reviewer 1 asks whether there is any support or validation for the detected intergenic events. In the NGS-ProToCol prostate cancer dataset, three intergenic TMPRSS2-ERG variants were highlighted (Figure S19) supported by not only high covered genomic breakpoints but also additional cryptic exons fitting the junction and well covered introns. For validation, we have assessed the overlap of partial intergenic intronic junctions with WGS entries for the BrCa chromothripsis events visualized in Figure S18. This showed that 14/18 investigated entries had matching WGS results. For intergenic exonic junctions, we have no means for validation. Figure S18 was updated accordingly. The following sentences in the discussion:<br>"Here, we confirm by utilizing Dr. Disco, that RNA-seq data be used to reveal genomic breakpoints of expressed transcripts, including intergenic translocations, in an automated fashion. Detection was limited to approximately 7% of WGS detected breakpoints but markedly higher for the driver TMPRSS2-ERG fusion gene (85% detected; 100% presence)."<br>Into:<br>"Here, we confirm by utilising Dr. Disco, that RNA-seq data can be used to reveal genomic breakpoints of expressed transcripts in an automated fashion. Detection was limited to approximately 7% of WGS detected breakpoints but markedly higher for the driver TMPRSS2-ERG fusion gene (85% detected; 100% presence). As the algorithm was conservative in detecting mRNA fusions, it is likely that genomic breakpoints were missed and the actual percentage is somewhat higher. Conversely, estimation of this percentage implies that WGS results offer the ground truth but these are also affected by noise, coverage and filter cut-offs, indicating this percentage is an approximation. The results commonly included intergenic junctions. For instance, three TMPRSS2-ERG fusions had their breakpoint located before ERG, supported by cryptic intergenic splice junctions and intergenic pre-mRNA coverage (Figure S19). Furthermore, intronic intergenic junctions in chromothripsis regions in three BrCa samples were in 14/18 cases validated by WGS junctions (Figure S18A)."<br><br>Reviewer #2:<br>Q: please re-phrase the sentence "..,Dr. Disco, that searchers for fusion transcripts without being restricted to splice junctions or annotated exons or genes." such that it is stated what Dr.Disco is using when searching (please, do not use "without being restricted")<br>A: We have rephrased the sentence to "We have developed an algorithm, Dr. Disco that searches for fusion transcripts by taking an entire reference genome into account as search space. This includes exons but also introns, intergenic regions and sequences that do not meet splice junction motifs."<br><br>Q: please re-phrase the sentence "These normal adjacent tissue samples were most likely..." something like "These normal looking adjacent..." because it may very well be that they are not normal even that they look like.<br>A: We have rephrased them into the following sentences:<br>"In two normal looking adjacent prostate samples, intronic and exonic junctions were found that were exactly identical to junctions in their matching malignant sample."<br>"These normal looking adjacent tissue samples were most likely contaminated with cancer cells (Figure S9B)."<br><br>Reviewer #3:<br>-
**Additional Information:**	
**Question**	**Response**
Are you submitting this manuscript to a special series or article collection?	No

Powered by Editorial Manager® and ProduXion Manager® from Aries Systems Corporation

**Experimental design and statistics**<br><br>Full details of the experimental design and statistical methods used should be given in the Methods section, as detailed in our Minimum Standards Reporting Checklist. Information essential to interpreting the data presented should be made available in the figure legends.<br><br>Have you included all the information requested in your manuscript?	Yes
**Resources**<br><br>A description of all resources used, including antibodies, cell lines, animals and software tools, with enough information to allow them to be uniquely identified, should be included in the Methods section. Authors are strongly encouraged to cite Research Resource Identifiers (RRIDs) for antibodies, model organisms and tools, where possible.<br><br>Have you included the information requested as detailed in our Minimum Standards Reporting Checklist?	Yes
**Availability of data and materials**<br><br>All datasets and code on which the conclusions of the paper rely must be either included in your submission or deposited in publicly available repositories (where available and ethically appropriate), referencing such data using a unique identifier in the references and in the "Availability of Data and Materials" section of your manuscript.<br><br>Have you have met the above requirement as detailed in our Minimum Standards Reporting Checklist?	Yes

Click here to access/download;LaTeX - Main Document (TeX file);main.tex

```
This is pdfTeX, Version 3.14159265-2.6-1.40.21 (TeX Live 2020/W32TeX)
(preloaded format=pdflatex 2020.5.12)  8 OCT 2021 08:24
entering extended mode
 restricted \write18 enabled.
 %&-line parsing enabled.
**main.tex
(./main.tex
LaTeX2e <2020-02-02> patch level 5
L3 programming layer <2020-05-05>

! LaTeX Error: File `oup-contemporary.cls' not found.

Type X to quit or <RETURN> to proceed,
or enter new name. (Default extension: cls)

Enter file name:
! Emergency stop.
<read *>

l.11 ^^M

*** (cannot \read from terminal in nonstop modes)


Here is how much of TeX's memory you used:
 22 strings out of 480681
 490 string characters out of 5908536
 236875 words of memory out of 5000000
 15943 multiletter control sequences out of 15000+600000
 532338 words of font info for 24 fonts, out of 8000000 for 9000
 1141 hyphenation exceptions out of 8191
 14i,0n,17p,95b,10s stack positions out of
5000i,500n,10000p,200000b,80000s
!  ==> Fatal error occurred, no output PDF file produced!
```

Figure 5

Click here to access/download;LaTeX - Figure (eps, ps, etc.);figure-5.pdf

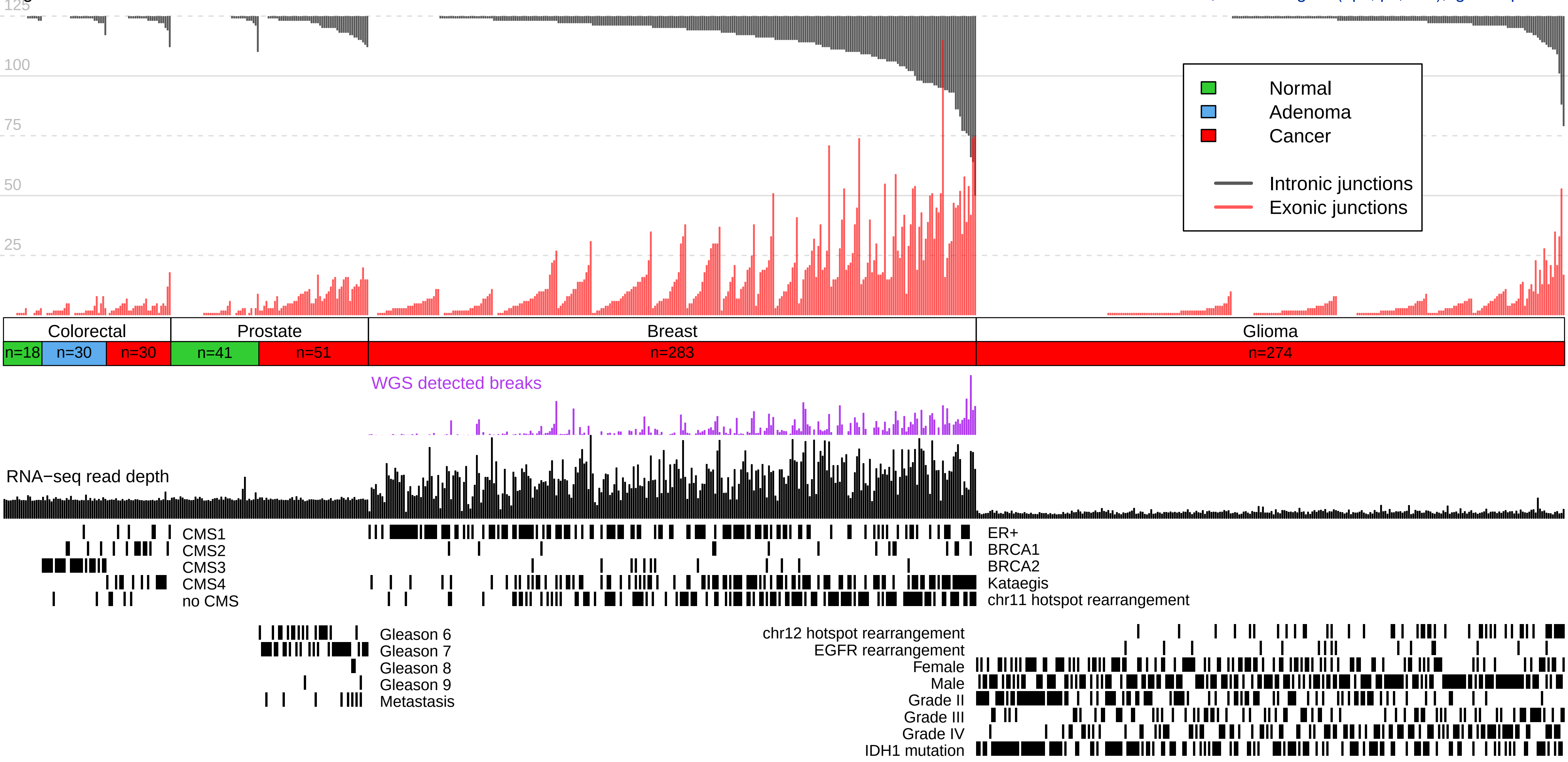

Figure 6

Click here to access/download;LaTeX - Figure (eps, ps, etc.);figure-6.pdf

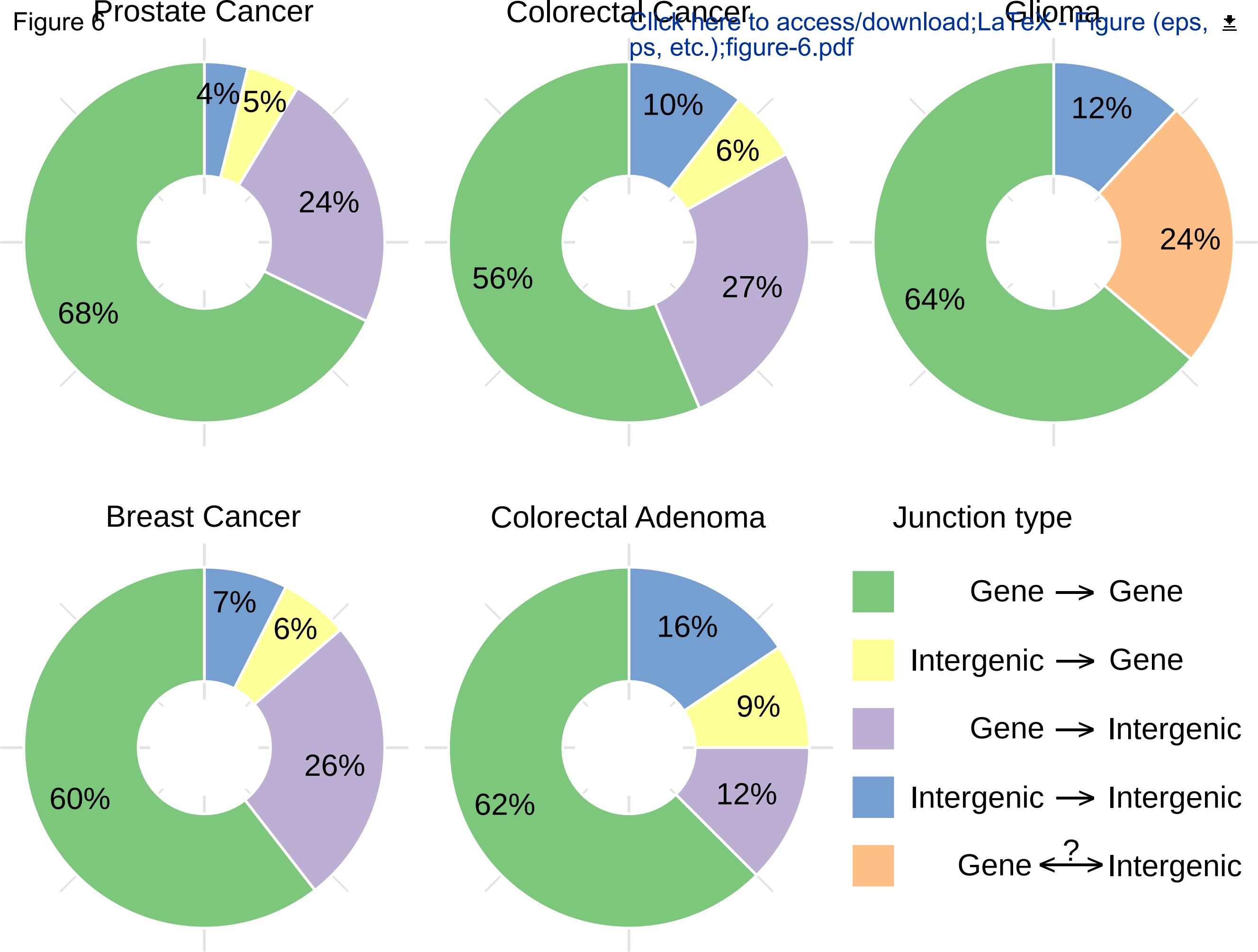

Figure 7

Click here to access/download;LaTeX - Figure (eps, ps, etc.);figure-7.pdf

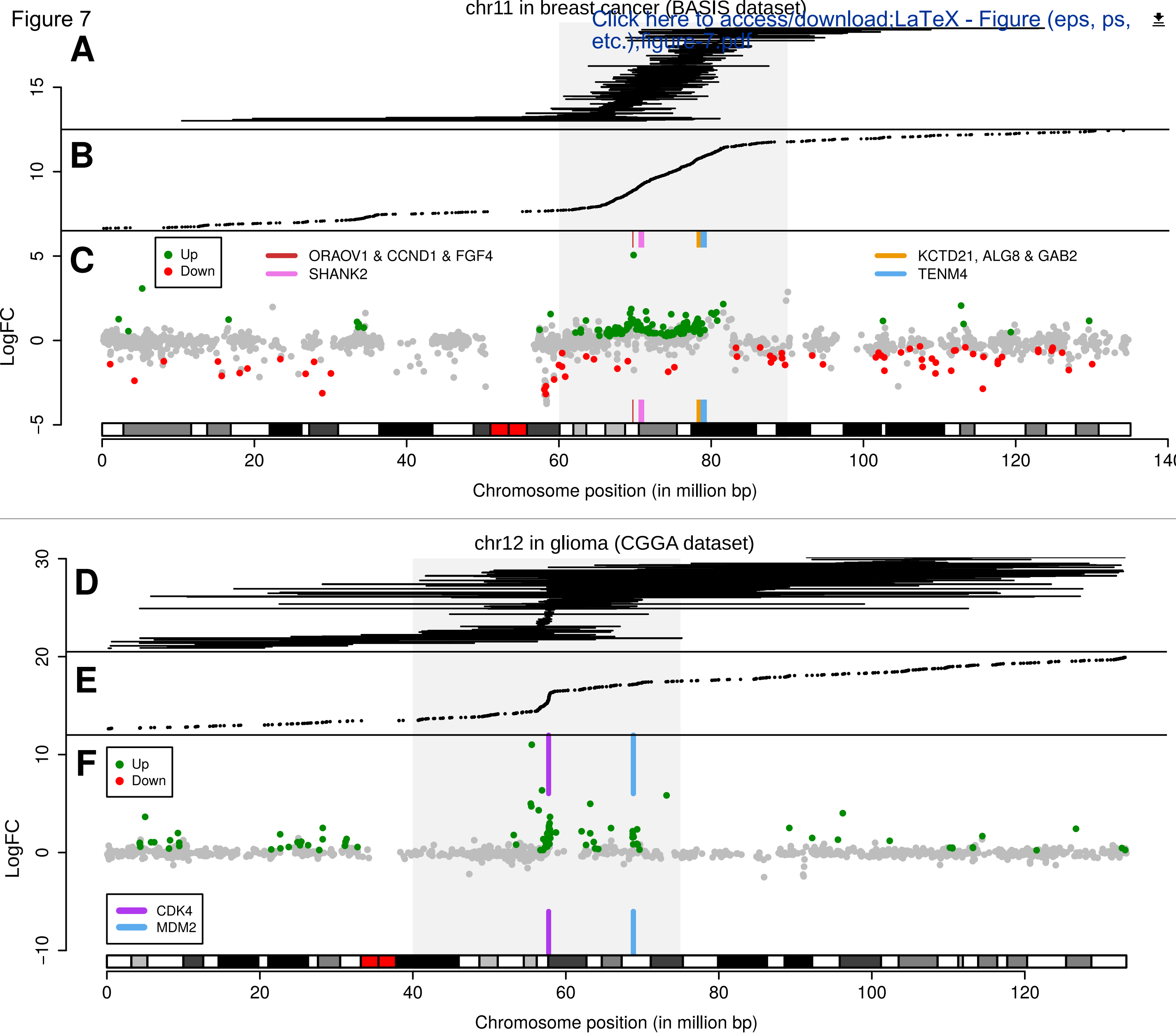

Figure 8

Click here to access/download;LaTeX - Figure (eps, ps, etc.);figure-8.pdf

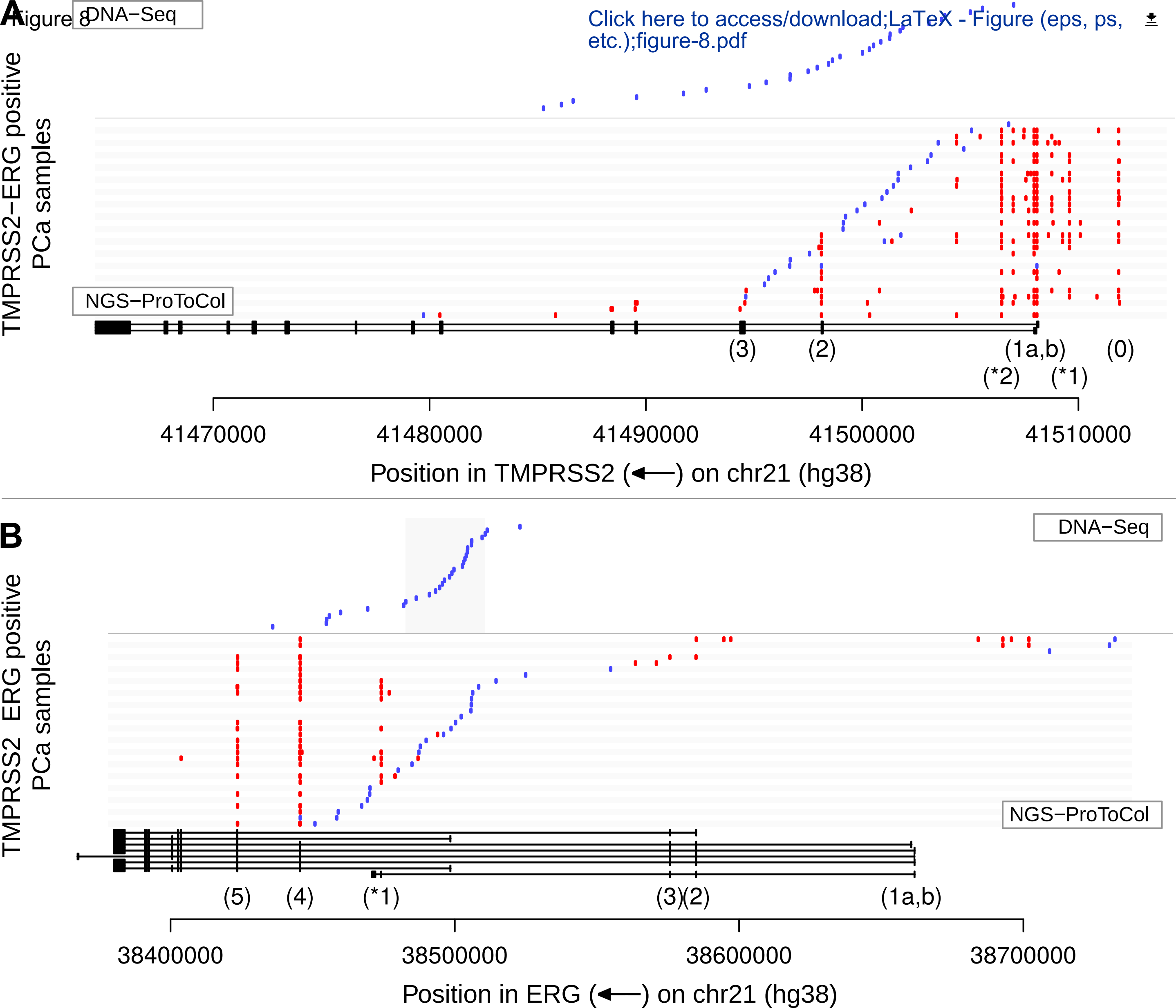

Figure 1

Click here to access/download;LaTeX - Figure (eps, ps, etc.);figure-1.pdf

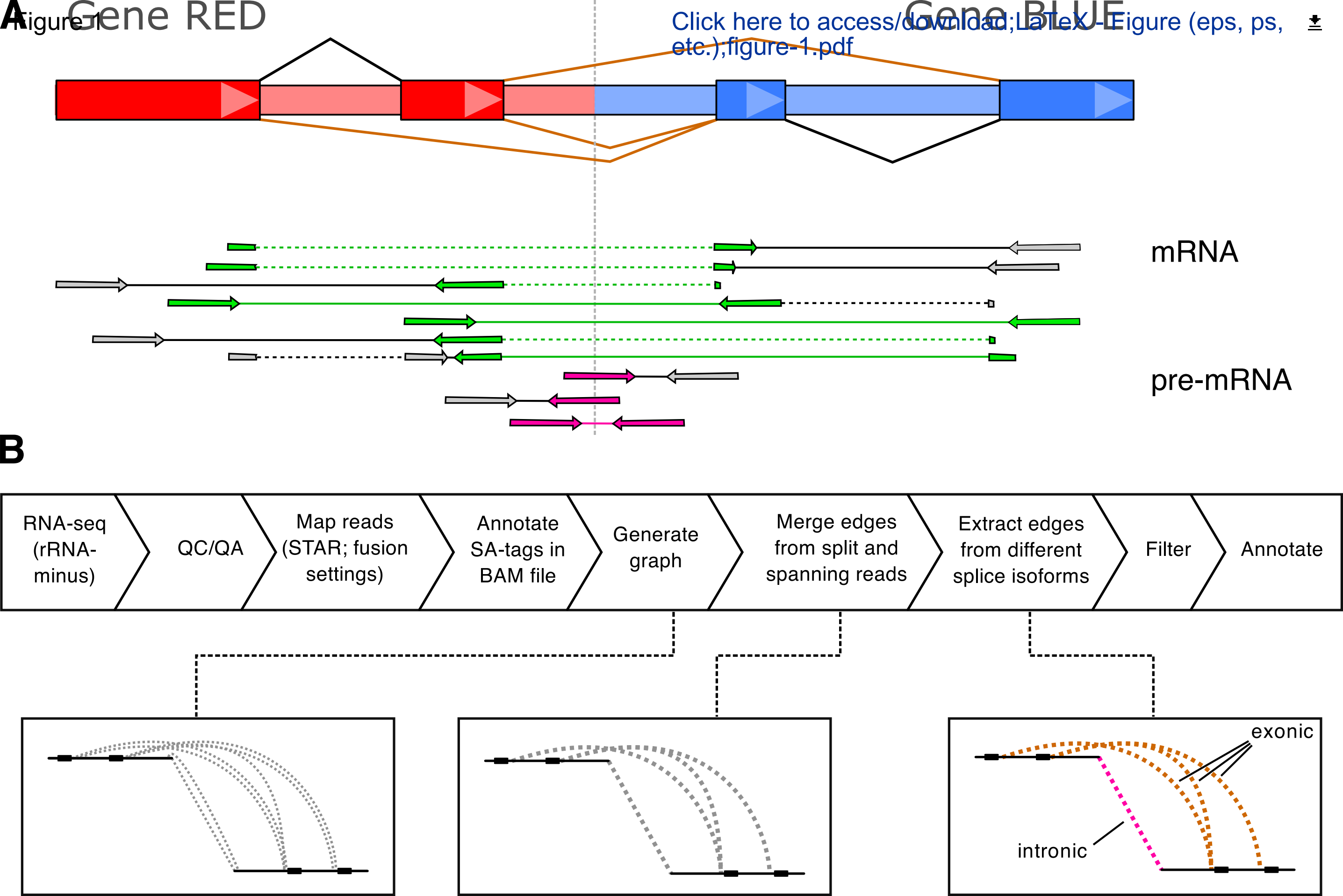

Figure 2

Click here to access/download;LaTeX - Figure (eps, ps, etc.);figure-2_revised.pdf

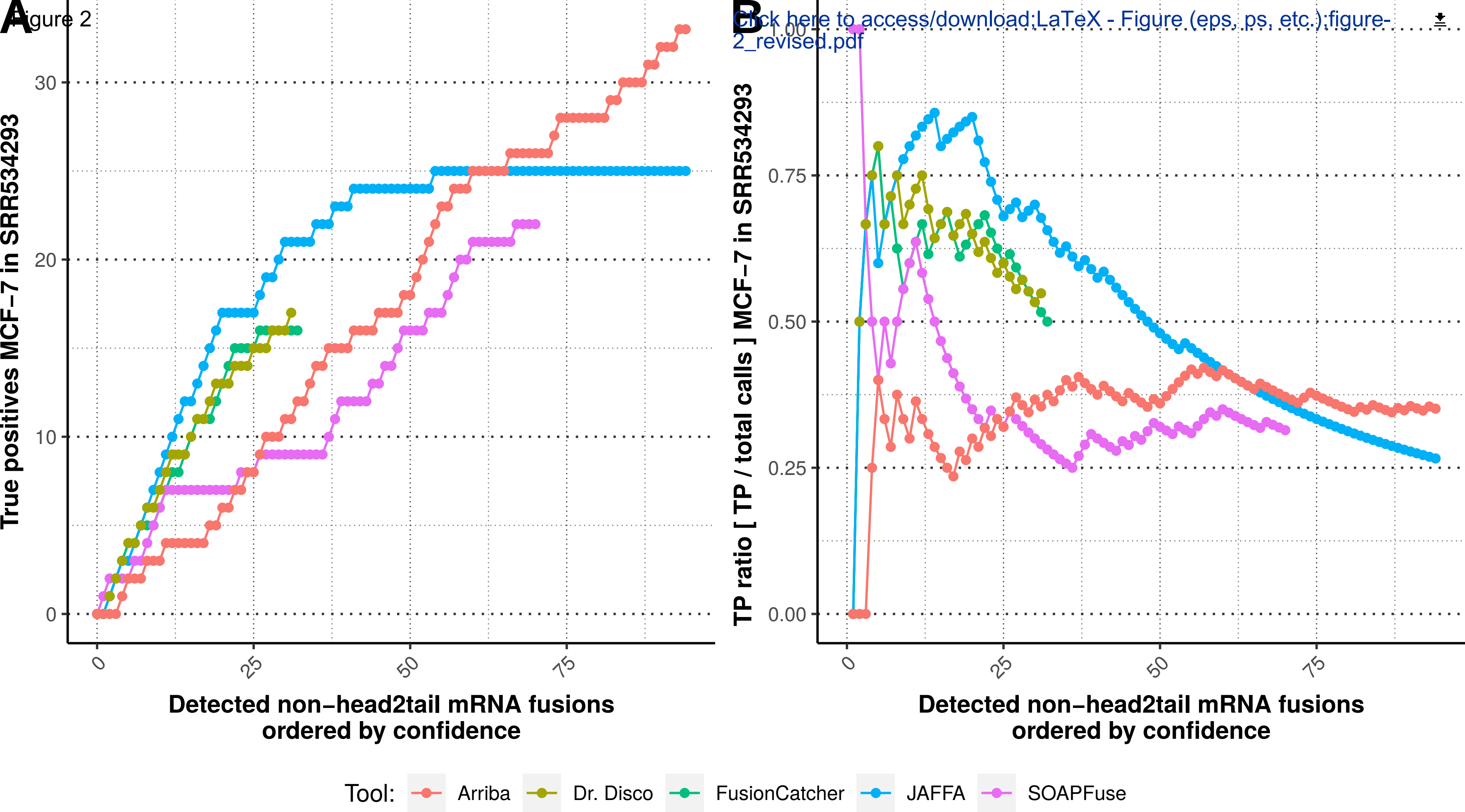

Figure 3

Click here to access/download;LaTeX - Figure (eps, ps, etc.);figure-3.pdf

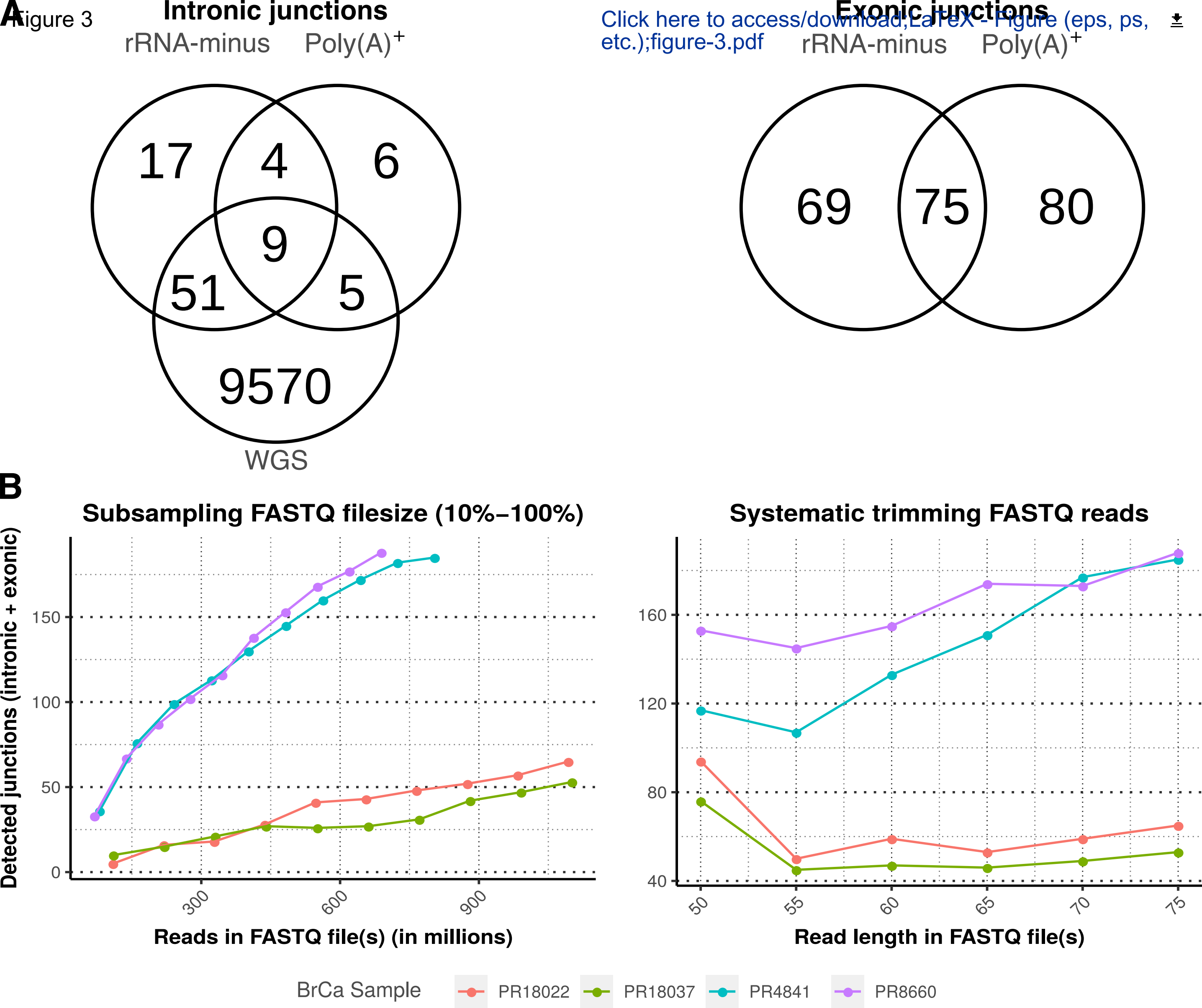

Figure 4

Click here to access/download;LaTeX - Figure (eps, ps, etc.);figure-4.pdf

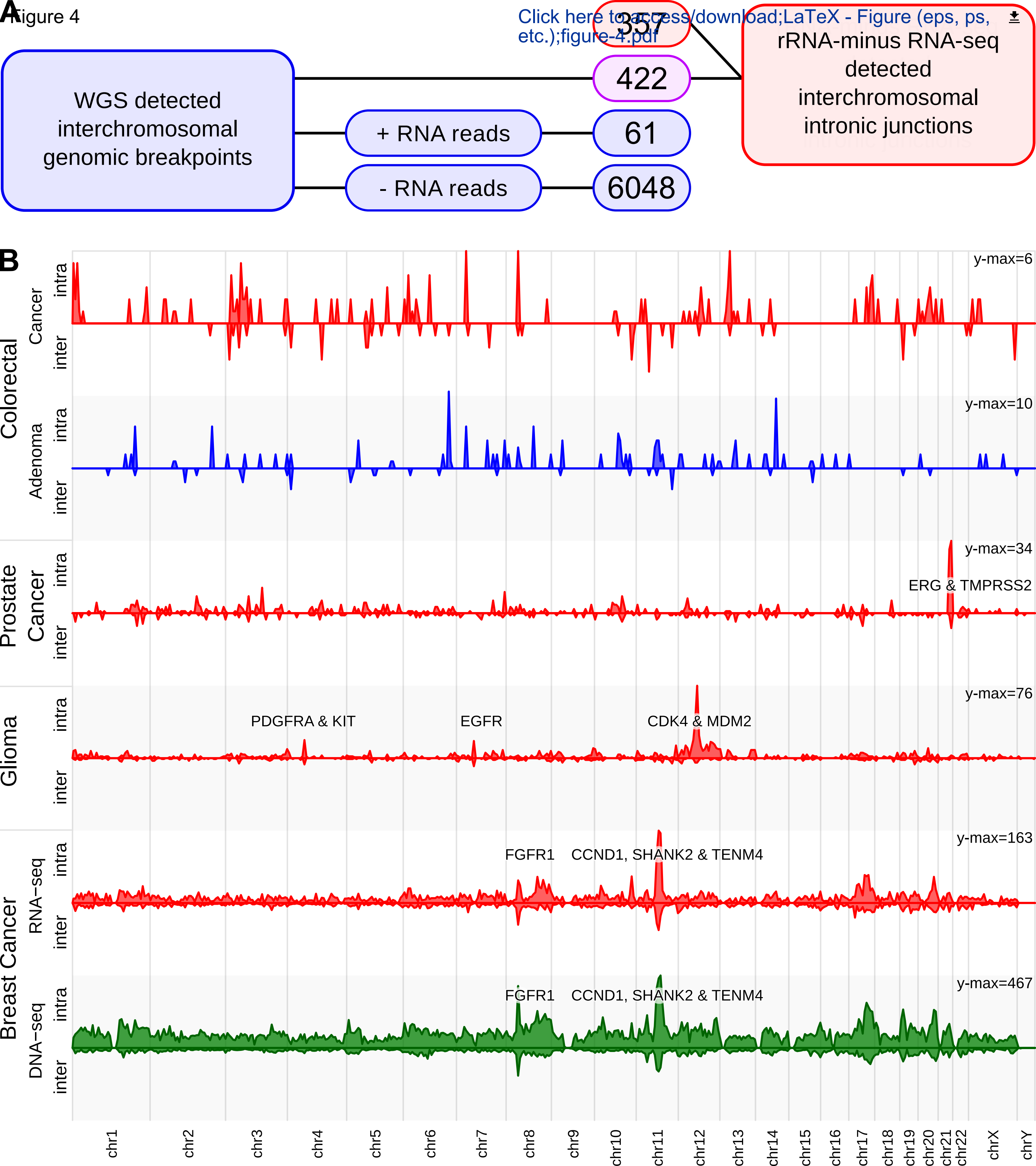

Manuscript

Click here to access/download;Manuscript;Dr__Disco_revised.pdf

OXFORD (GIGA)^n SCIENCE

GigaScience, 2021, 1–15

doi: xx.xxxx/xxxx
Manuscript in Preparation
Technical Note

TECHNICAL NOTE

Fusion transcripts and their genomic breakpoints in poly(A)+ and rRNA-minus RNA sequencing data

Youri Hoogstrate[1,2,*], Malgorzata A. Komor[3], René Böttcher[1,4], Job van Riet[5], Harmen J.G. van de Werken[1,6], Stef van Lieshout[7], Ralf Hoffmann[8], Evert van den Broek[3,9], Anne S. Bolijn[3], Natasja Dits[1], Daoud Sie[3], David van der Meer[11], Floor Pepers[11], Chris H. Bangma[1], Geert J.L.H. van Leenders[10], Marcel Smid[5], Pim J. French[2], John W.M. Martens[5], Wilbert van Workum[14], Peter J. van der Spek[10], Bart Janssen[11], Eric Caldenhoven[12], Christian Rausch[13], Mark de Jong[15], Andrew P. Stubbs[10], Gerrit A. Meijer[3], Remond J.A. Fijneman[3] and Guido W. Jenster[1]

[1]Department of Urology, Erasmus Medical Center, Rotterdam, 3015GD, The Netherlands and [2]Department of Neurology, Erasmus Medical Center, Rotterdam, 3015GD, The Netherlands and [3]Department of Pathology, Netherlands Cancer Institute, Amsterdam, 3015GD, The Netherlands and [4]Department of Life Sciences, Barcelona Supercomputing Center, Barcelona, 08034, Spain and [5]Department of Medical Oncology, Erasmus Medical Center, Rotterdam, 3015GD, The Netherlands and [6]Cancer Computational Biology Center, Erasmus Medical Center, Rotterdam, 3015GD, The Netherlands and [7]Hartwig Medical Foundation, Amsterdam, 1098XH, The Netherlands and [8]Philips Research, Eindhoven, 5656AE, The Netherlands and [9]Department of Pathology and Medical Biology, University Medical Center Groningen, Groningen, 9713GZ, The Netherlands and [10]Department of Pathology, Erasmus Medical Center, Rotterdam, 3015GD, The Netherlands and [11]GenomeScan, Leiden, 2333BZ, The Netherlands and [12]Lygature, Utrecht, 3521AL, The Netherlands and [13]BioLizard N.V., Ghent, 9000, Belgium and [14]Limes Innovations, The Netherlands and [15]VHLGenetics, Wageningen, 6708PW, The Netherlands

[*]**Correspondence:** Youri Hoogstrate, Department of Neurology, Erasmus MC, PO Box 2040, 3000CA, Rotterdam, The Netherlands, E-mail: y.hoogstrate@erasmusmc.nl.

Abstract

Background: Fusion genes are typically identified by RNA-seq without elucidating the causal genomic breakpoints. However, non poly(A)-enriched RNA-seq contains large proportions of intronic reads spanning also genomic breakpoints.
Results: We have developed an algorithm, Dr. Disco, that searches for fusion transcripts by taking an entire reference genome into account as search space. This includes exons but also introns, intergenic regions and sequences that do not meet splice junction motifs. Using 1,275 RNA-seq samples, we investigated to what extent genomic breakpoints can be extracted from RNA-seq data and their implications regarding poly(A)+ and rRNA-minus RNA-seq data. Comparison with WGS data revealed that most genomic breakpoints are not, or minimally, transcribed while, in contrast, the genomic breakpoints of all 32 *TMPRSS2-ERG* positive tumors were present at RNA level. We also revealed tumors in which the *ERG* breakpoint was located before ERG, which co-existed with additional deletions and mRNA that incorporated intergenic cryptic exons. In breast cancer we identified rearrangement hotspots near *CCND1* and in glioma near *CDK4* and *MDM2* and could directly associate this with increased expression. Furthermore, in all datasets we find fusions to intergenic regions, often spanning multiple cryptic exons that potentially encode neo-antigens. Thus, fusion transcripts other than classical gene-to-gene fusions are prominently present and can be identified using RNA-seq.
Conclusion: By using the full potential of non poly(A)-enriched RNA-seq data, sophisticated analysis can reliably identify expressed genomic breakpoints and their transcriptional effects.

Key words: Gene Fusion; RNA Precursors; RNA-Seq; Chromosome Breakage; Genomic Structural Variation; cryptic exons; TMPRSS2-ERG

Key Points

- Using sophisticated analysis, expressed genomic breakpoints can be revealed in RNA-seq data.
- rRNA-minus RNA-seq data harbours more genomic breakpoints than poly(A)+.
- Inclusion of cryptic exons as result of genomic rearrangements (often intergenic but also as anti-sense in-gene) are common in cancer.

Compiled on: October 8, 2021.
Draft manuscript prepared by the author.

Findings

Background

Genomic rearrangements are frequently observed in cancer and can drive disease initiation and progression through disruption of tumour suppressor genes and activation of oncogenes [1, 2, 3]. Marked examples include *TMPRSS2-ERG* fusions in prostate adenocarcinoma (PCa) [4] and *BCR-ABL* in chronic myelogenous leukaemia [5]. DNA rearrangements and their aberrant ligations are identified as genomic breakpoints by whole genome sequencing (WGS) but their potential role as driver mutation is mostly unresolved as-of-yet. The majority of genomic breakpoints involve intergenic regions and are thus typically not located in messenger RNA (mRNA) and protein coding sequences [6]. Moreover, genomic breakpoints of fusion genes are mostly located intronic [7]. To reveal their downstream effects, RNA-sequencing (RNA-seq) is crucial to investigate changes at the transcriptional level and identify actual (in-frame) fusion transcripts. Conversely, for fusion-transcripts, identification of the exact genomic breakpoint(s) can be essential to explain changes in gene expression and to define the origins of alternative promoter usage and altered splicing or polyadenylation events. Combined genomic and expression data allows to further study functional consequences of genomic rearrangements and signifies whether an event is merely a passenger or a putative driver mutation [7, 8]. However, for many transcriptome studies, the exact genomic breakpoints of expressed rearrangements have not been resolved as matched whole-genome sequencing (WGS), Sanger sequencing, or similar analyses were not performed. Therefore, we set out to determine whether genomic breakpoints could be identified from RNA-seq data.

Next to targeted gene approaches, there are two main approaches in preparing RNA-seq libraries [9]. First, the more traditional method includes the positive selection of polyadenylated messenger RNA (mRNA; *poly(A)*$^+$) to specifically target mRNA and eliminate abundant ribosomal RNA (rRNA). Because splicing takes place mostly co-transcriptionally, pre-mRNA is typically not polyadenylated and thus not included in this approach. Alternatively, one may extract total RNA and use random hexamer primers to initiate cDNA synthesis while removing abundant unwanted RNAs by various additional methods. This approach is referred to as *rRNA-minus* and is commonly applied when (partially) degraded RNA from formalin-fixed paraffin-embedded (FFPE) samples is sequenced.

rRNA-minus RNA-seq is thus capable of identifying non-poly(A) transcripts such as circRNAs, specific types of small and long non-coding RNAs and, importantly, actively-transcribed precursor mRNAs (pre-mRNAs) [10]. Although the exact numbers depend on the used protocol, tissue type, lariats [11] and intron lengths, typically 30-40% of rRNA-minus RNA-seq reads map to intronic features, compared with 5-10% in poly(A)$^+$ RNA-seq [12]. Therefore, rRNA-minus RNA-seq datasets require at least a 50% higher sequencing depth to achieve an exon coverage comparable to poly(A)$^+$ RNA-seq, while being capable of identifying additional RNA classes [9].

Fusion genes such as *TMPRSS2-ERG* and *BCR-ABL* are frequently observed as drivers within their respective malignant tissue [13]. Yet, many observed fusion genes are still of unknown consequence and seen in small frequencies in various cancer types. RNA-seq is highly suitable for fusion gene detection [14, 15, 16].

Methods to integrate RNA fusions with genomic breakpoints allow to further assess functional consequences [7, 8, 17]. They are even capable of integrating complex higher order rearrangements, but remain dependent on the availability of matching DNA data. State of the art fusion-detection tools such as FusionMap, FusionCatcher and JAFFA focus on exon regions or splice junctions specifically [18, 19, 20], which are the main target of poly(A)$^+$ RNA-seq. Indeed, these tools also work well on rRNA-minus RNA-seq as these also include exonic reads. Their efficient search space reduction in turn reduces the overall complexity and processing time. However, using rRNA-minus RNA-seq, typically 30-40% of the aligned reads are intronic and a further 20-25% of all reads are found to be intergenic [12], which are often *a priori* neglected. This large proportion of intronic and intergenic reads provides an opportunity to identify additional cancer-specific transcripts and exact genomic breakpoints of fusion genes. We have shown in a proof-of-concept that rRNA-minus RNA-seq can indeed identify genomic breakpoints [10].

Here, we leverage a novel algorithm named Dr. Disco to report on the presence of genomic breakpoints in RNA-seq data, its implications regarding poly(A)$^+$ and rRNA-minus RNA-seq and on fused cryptic exons. The algorithm computationally identifies such genomic breakpoints and exon-to-exon junctions in a genome-wide fashion, taking into account the potential of rRNA-minus RNA-seq. It was applied on six RNA-seq datasets spanning multiple malignant tissue types (n=1,275) (**Table 1**). Indeed, we reveal exact causal genomic breakpoints as derived from RNA-sequencing alone but limited to regions sufficiently expressed such as fusion gene *TMPRSS2-ERG*. Furthermore, rRNA-minus RNA-seq data can reveal more transcriptionally active rearrangements than poly(A)$^+$ RNA-seq and results can be useful to supplement WGS. While only large datasets were analyzed in this study, the method is developed for single-sample analysis. In summary, rRNA-minus RNA-seq in combination with a suited analysis pipeline gives a more complete view on both the origin and effects of genomic rearrangements and their direct influence on the expression of associated genes.

Data Description

RNA-seq data from several types of malignant tissue were used. For the NGS-ProToCol datasets (normal adjacent prostate; n=41, prostate cancer; n=51; normal adjacent colon; n=18, colorectal adenoma; n=30 and colorectal carcinoma; n=30), carcinoma, adenoma (only colon) and adjacent normal tissue were rRNA-minus sequenced to study condition-specific molecular differences and further stratify tumour types [21, 22]. The PCa-LINES dataset consists of PCa cell lines PC346C and VCaP and additional PCa patient samples G-089, G-110, G-295, G-316 and G-346, which were WGS, rRNA-minus RNA and poly(A)$^+$ RNA sequenced. The included VCaP cell-line is commonly used as model system for prostate cancer and is known to contain the *TMPRSS2-ERG* fusion [23, 24]. The BASIS dataset consists of 560 WGS sequenced breast cancer samples [25] and 289 rRNA-minus RNA samples [26, 27], of which 207 are matching. The

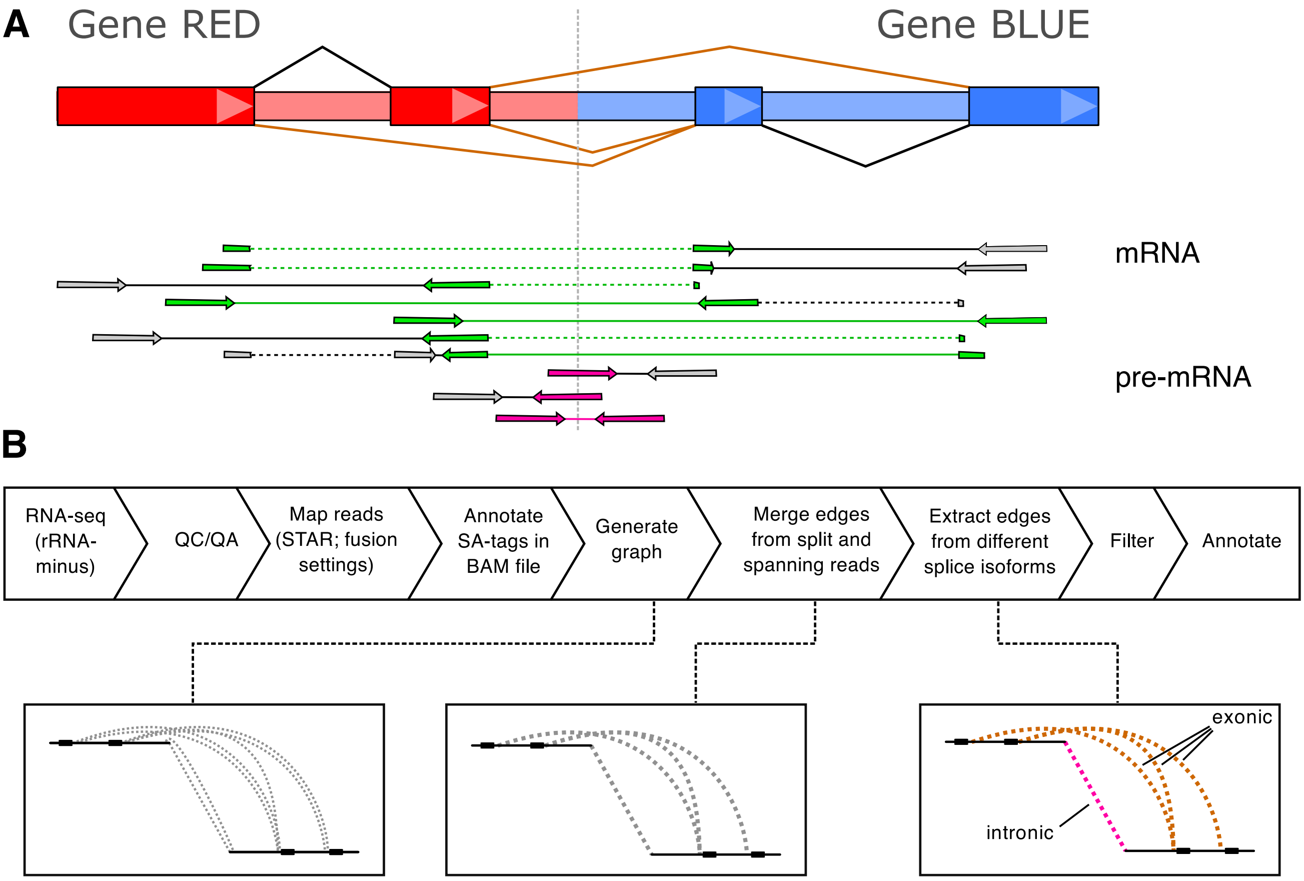

Figure 1. Overview Dr. Disco pipeline and principle. (**A**) Schematic representation of fusion-gene RED-BLUE. Due to relatively large intron sizes, in-gene genomic breakpoints occur most often intronic. The fusion results in different fusion splice isoforms (brown). Fusion splice junction spanning reads form the classical evidence for mature mRNA fusion-events. In rRNA-minus data, intronic pre-mRNA reads (pink) may cover causal genomic breakpoints. (**B**) Pipeline flowchart: RNA-seq data is aligned. Discordant reads are transformed into edges and inserted into a breakpoint graph. In the graph, intronic or exonic derived edges are kept separate. Detection of junctions is performed by analysing the graph for clusters. An additional splice variant correction is applied. Identified junctions are filtered, annotated and marked intronic or exonic.

Chinese Glioma Atlas (CGGA) is composed of 274 rRNA-minus RNA-seq samples of various types of gliomas [28]. MCF-7 cell line data from ENCODE [29] was used for validation as it is a commonly used golden standard dataset [20]. We made the NGS-ProToCol and PCa-LINES publicly available, other data was taken from the public domain (**Table 1**).

To identify exact genomic breakpoints from rRNA-minus RNA-seq, we developed and implemented a novel algorithm, termed Dr. Disco. Briefly, it uses reads with a split alignment or read pairs with an inverted orientation or with a large insert size: discordant reads [30]. It uses reads not only from exons but also intronic and intergenic regions (**Figure 1** and **Supplementary Dr. Disco Technical Specification**). Discordant reads are transformed and inserted into a breakpoint graph [7]. The breakpoint graph, which contains junctions derived from RNA data only, is then extensively analysed to find clusters, resolve splicing and keep junctions from distinct events separated.

For terminology, we define exon-to-exon splice fusion junctions (*exonic junctions*) as junctions that result from splicing and of which it may be expected that they could be detected by classical fusion detection algorithms. These also include fusions to not annotated (cryptic) exons.

Fusion transcripts which are not a result of (cryptic) exon-to-exon splicing are typically intron-to-intron junctions, spanning genomic breakpoints. Note, it is possible that genomic breakpoints are located within exons and do not result in fused spliced junctions (**Figure S1**). Because intron-to-intron junctions are not the product of splicing and are not the primary target of classical fusion gene detection, we consider these *intronic*. After the graph is analysed, corresponding detected junctions are marked *exonic* or *intronic* accordingly. The detailed computational methodology is described in **Supplementary Methods** and **Supplementary Dr. Disco technical specification**. The method was used to perform analyses in particular to study junctions in RNA data beyond classical fusion genes.

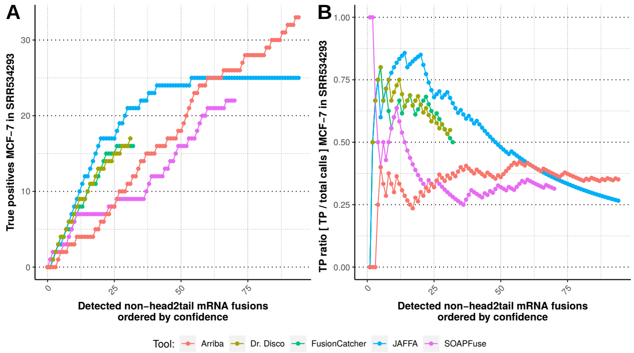

Figure 2. Evaluation on ENCODE MCF-7 dataset [20, 29]. For our algorithm, only non head-to-tail junctions located on both sides at annotated exons were included.

Evaluation Poly(A)+ detectors

The performance of the algorithm identifying mRNA fusions was assessed by analysing the ENCODE MCF-7 dataset, which was subsequently compared to poly(A)$^+$ detector results published earlier [20] (**Figure 2**) and with Arriba [31], which also makes use of STAR as aligner. Our method was not superior to JAFFA, and performed rather similar to FusionCatcher. It was mostly limited in the total number of true positives identified, indicating it is more conservative than JAFFA, SOAPFuse and Arriba. Although the true positives ratio for Arriba was considerably lower, the total amount of true positives was the highest.

Interestingly, unreported fusions spanning cryptic exons and intergenic regions were observed, including: *ATXN7*-chr1:106,216,304 resulting in a fusion to cryptic intergenic exons supported by a matching genomic breakpoint (chr3:6,394,8014-chr1:106,192,959) and *PRPF18*-*BEND7*, resulting in anti-sense transcription of *BEND7* spanning cryptic ingene exons (**Figure S2**).

Dr. Disco's performance of detecting only mRNA exon junctions was comparable but not superior to existing tools while it revealed 27 additional high confidence junctions using cryptic exons (**Table S1**). The time it took Dr. Disco to complete analysis after the STAR alignment was 949 seconds, 2.07 times slower compared with Arriba (458 seconds). This is in concordance with the expectation that analysing a larger search space requires more conservative filtering and takes more resources to complete.

Comparison poly(A)+ and rRNA-minus RNA-seq

Results from seven PCa samples with matching rRNA-minus and poly(A)$^+$ RNA-seq (PCa-LINES dataset) were compared (**Figure 3A**). Contrary to our initial hypothesis, the poly(A)$^+$ results also revealed intronic junctions, representing genomic breakpoints. Still, rRNA-minus data identified (3.4×) more intronic junctions as compared to poly(A)$^+$ RNA-seq. The intronic junctions identified in poly(A)$^+$ often have lower read counts or were located in UTR terminal exons as in-exon located genomic breakpoints (**Figure S3**). Terminal exons are known for their relative large size as they are approximately 6-7 times larger than internal exons [32]. The number of exonic junctions, thus predicted mRNA fusions, was nearly identical for rRNA-minus and poly(A)$^+$ RNA-seq (144 vs. 155).

Comparison of RNA- with DNA-seq data

Within the PCa-LINES dataset, the number of WGS identified genomic breakpoints vastly outnumbered those extracted from the rRNA-minus RNA-seq (6.8%), indicating that only a fraction of the genomic rearrangements is expressed at a level to be detected by rRNA-minus RNA-seq. Both intronic and exonic junctions from both rRNA-minus and poly(A)$^+$ data co-located near WGS detected genomic breakpoints (**Figure S4**), confirming their validity.

Four breast cancer (BrCa) RNA-seq samples from the BASIS dataset [25, 26] were used to assess the influence of sequencing coverage and read length. Systematically truncating the reads showed that the number of detected junctions dropped as sequencing reads became shorter (**Figure 3B**). From a read length below 55 nt, the number of detected junctions increased. This was due to an overall increase in mis-alignments that do not resemble actual evidence of genomic rearrangements, indicating that a minimum length of 55 bp is needed for accurate detection. Irrespective of the number of genomic breakpoints present within a sample as determined by WGS, an increase

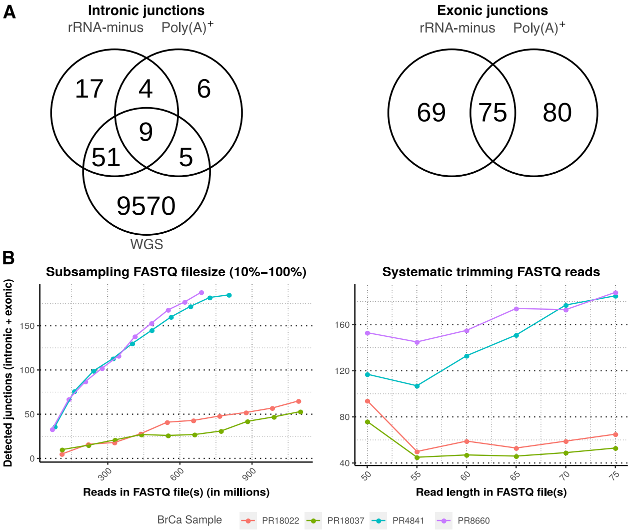

Figure 3. Overlap across sequencing types and library size influence. (**A**) Overlap of cumulative interchromosomal junctions of 7 WGS PCa samples rRNA-minus and poly(A)$^+$ RNA-seq (PCa-LINES dataset). Overlap in only intronic junctions representing genomic breakpoints (left) and only exonic splice junctions (right). Of the 69 exonic junctions only found in rRNA-minus RNA-seq, 40 were detected in the matching poly(A)$^+$ but did not pass filtering. Of the 80 poly(A)$^+$-only exonic junctions, 58 were found in rRNA-minus but did not pass filtering. Not passing filtering mostly occurred because of insufficient discordant reads. (**B**) The number of predicted junctions as function of sequencing depth (left) and read-length (right) reduction. BrCa samples were selected for high sequencing depth (PR18022 and PR18037) or a high number of junctions (PR4841 and PR8660). Left: The number of predicted junctions per sequencing-depth (10-100%) with the full read-length (2x75 bp). Reducing the sequencing depth, also for samples with a high sequencing depth, reduces the number of detected junctions. Sample PR4841 reaches a plateau. Right: Each data point represents the number of predicted junctions per given read-length, at full sequencing-depth. Truncating sequencing-reads results in a lower number of predicted junctions. However, below 55 nucleotides the number of detected junctions increases.

in overall sequencing depth is positively correlated with an increase in detected junctions (**Figure 3B**).

All 207 WGS and rRNA-minus RNA-seq matching samples from the BASIS cohort [25, 26] were used to compare interchromosomal junctions. WGS identified a total of 6,531 interchromosomal genomic breakpoints, of which 422 (6.5%) were found in both assays (**Figure 4A**), a similar percentage as in PCa-LINES. Dr. Disco detected 357 unique genomic breakpoints which were only present within the RNA-seq data, of which 100 were identified within only eight BrCa samples which also had an overall high number of WGS-detected genomic breakpoints (**Figure S5**). The density of WGS and rRNA-minus detected junctions within chromosomal bins was highly similar (Pearson correlation: $r = 0.72$, **Figure 4B, S6-S8**), with prominent focal peaks near the genomic loci of *CCND1*, *SHANK2* and *FGFR1*.

Pan-cancer analysis

We analysed the results of the algorithm on rRNA-minus RNA-seq data (n=651) from different malignant tissue types (**Figures 4B,5**): the BASIS, NGS-ProToCol colon and prostate and CGGA datasets. (**Table 1**).

Intronic and exonic junctions were identified in each dataset. The different malignant tissue types showed distinct regions enriched with intronic and exonic junctions (**Figure 4B**). Known prominent events include *TMPRSS2*-*ERG* in PCa, *EGFR*, *CDK4* and *MDM2* in glioma and *CCND1* in BrCa. The breakpoints per sample and associated clinical parameters are

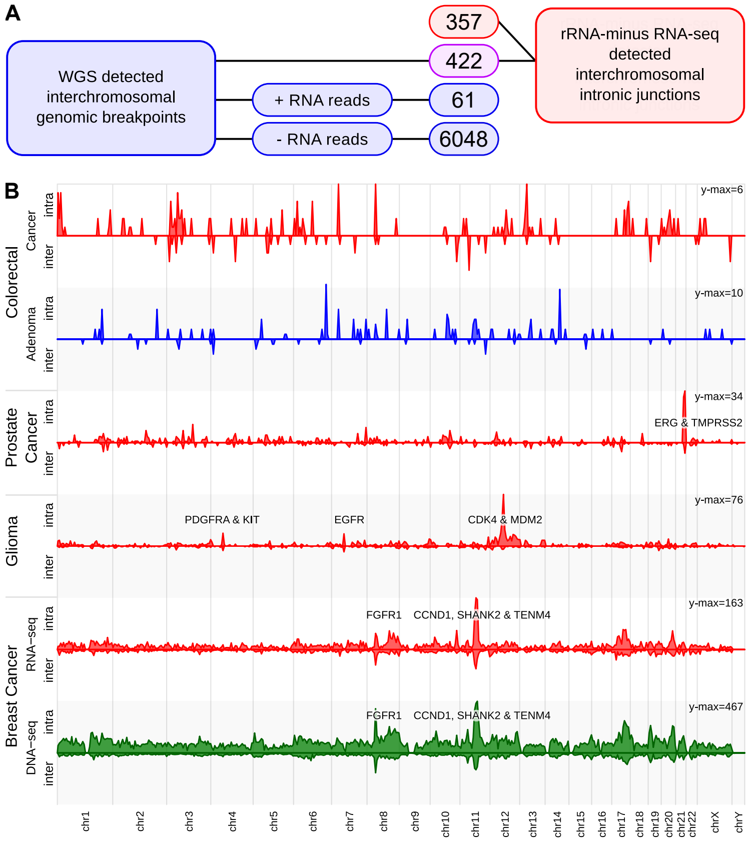

Figure 4. Integration RNA-seq analysis and WGS in BrCa. (**A**) Number of detected genomic breakpoints per subgroup in WGS and rRNA-minus RNA-seq data of 207 matching BrCa samples. Rectangles in blue indicate presence only in WGS data, in red only in RNA-seq data and in pink in both. To avoid artifacts from RNA post-processing such as circRNAs and read-throughs, only interchromosomal entries were interrogated. Of the interchromosomal WGS breakpoints, 6044 did not have sufficient discordant reads in the RNA-seq data. Of 62 genomic breakpoints, the threshold of sufficient discordant RNA-seq reads was exceeded, but it was not detected by Dr. Disco or did not pass filtering. 425 breakpoints were detected in both the assays and 361 RNA-seq detected breakpoints did not match a WGS entry. (**B**) Chromosome plot representing the binned density of inter and intrachromosomal intronic junctions. For the BrCa samples, Dr. Disco RNA-seq analysis (red) and WGS breakpoints (green) are depicted. The number of RNA-seq genomic breakpoints in the colorectal cancer and adenomas is low and no recurrent breakpoints were identified yet. The number of genomic breakpoints in colorectal adenomas was lower than in colorectal cancer. The observed peaks in colorectal cancer originated from multiple, sample specific, junctions (**Figure S10**).

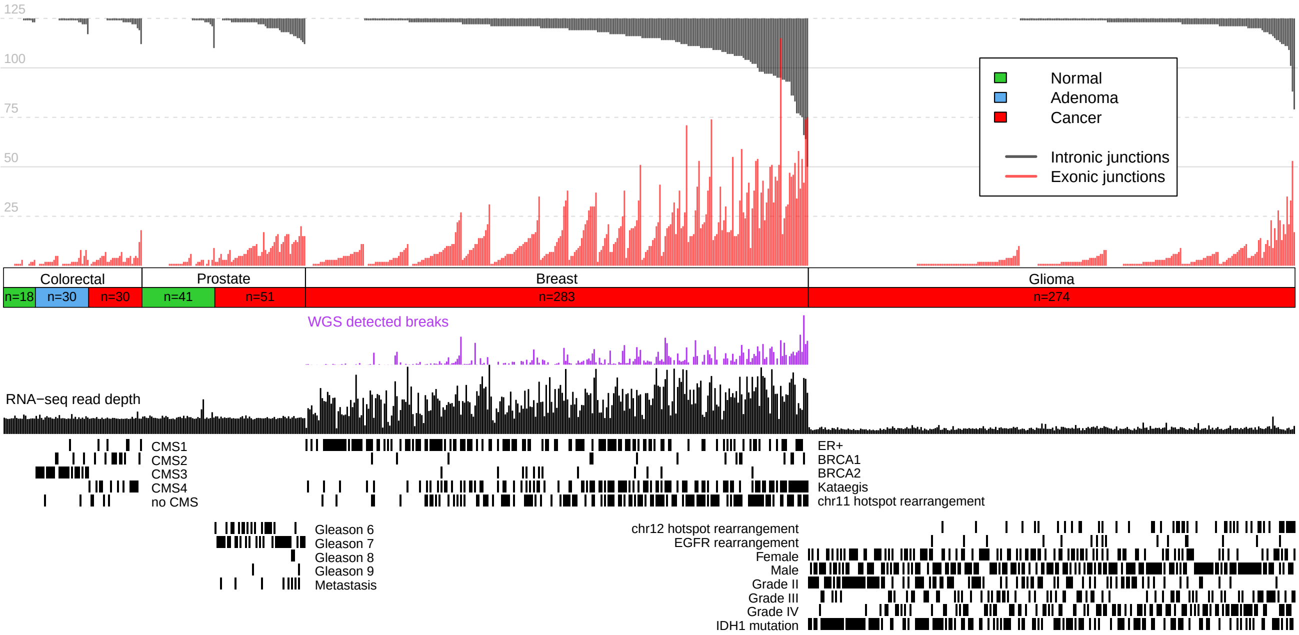

Figure 5. Pan-cancer results summary. Intronic and exonic junctions are given per sample for the NGS-ProToCol, BASIS and CGGA datasets with their associated clinical parameters. For the colon samples, the predicted CMS classes are provided, for the prostate cancer samples the Gleason grade and metastatic progression are provided, for the breast cancer samples the ER, BRCA1, BRCA2, kataegis and Dr. Disco detected chr11-hotspot status are provided and for the glioma samples the grading, recurrence, IDH1 mutation status, gender and the Dr. Disco detected EGFR and chr12 hotspot status are provided.

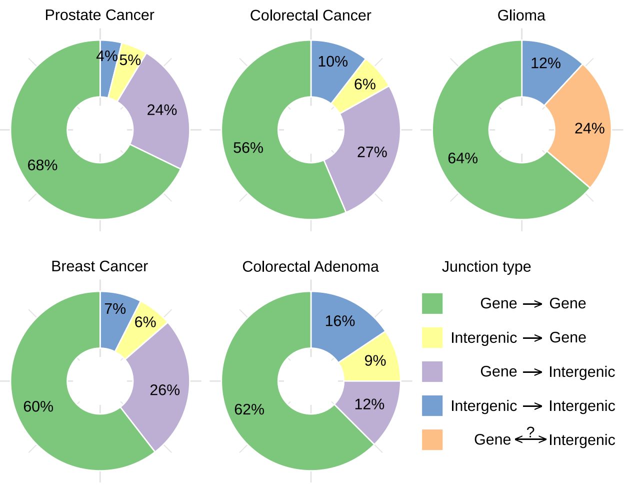

Figure 6. (Inter)genic junction status. Frequency of intronic and exonic junctions and their gene / intergenic status. Because the glioma dataset was sequenced unstranded, junctions with one intergenic side are grouped together. In all datasets, approximately 3/8 of the junctions have at least one intergenic side. Inter- and intrachromosomal junctions were included, suspected circRNAs were discarded, unlocalized and unplaced sequences (chrUn_...) and alternate loci (chr..._alt) spanning junctions were discarded. Intronic and exonic junctions corresponding to the same event were treated as single entry.

provided in **Figure 5**. The lowest average number of genomic breakpoints per tissue type was found in normal adjacent samples (colon=0.5; prostate=0.9) followed by colorectal adenoma (1.1) (**Figures S9-S10**). In two adjacent normal looking prostate samples, intronic and exonic junctions were found that were exactly identical to junctions in their matching malignant sample. These adjacent normal looking tissue samples were most likely contaminated with cancer cells (**Figure S9B**). Of the different malignant tissue types, colorectal cancer samples were characterized by the lowest average number of junctions (1.1) followed by combined low- and high-grade glioma (2.1) (**Figure S11**). Conversely, PCa (4.3) and BrCa (9.3) were characterized by relatively high numbers of genomic breakpoints per sample. These average numbers were not normalized for sequence depth as results are also influenced by dataset specific differences in read length, stranding, RNA quality and library preparation. Therefore, comparison of these average numbers of junctions is confounded by these factors.

Associations between the number of detected intronic junctions per sample and clinical parameters were investigated within datasets (**Figure 5**). In BrCa, presence of kataegis ($P = 1.9 \times 10^{-9}$) was positively associated with the number of observed intronic junctions whereas ER+ tumours were negatively associated ($P = 0.9 \times 10^{-3}$) with the number of intronic junctions. In glioma, tumour grade IV is positively associated with the number of intronic junctions per sample ($P = 1.1 \times 10^{-5}$), whereas tumour grade II ($P = 2.9 \times 10^{-8}$) and presence of IDH1 mutation ($P = 0.8 \times 10^{-3}$) is negatively associated. Although trends within PCa were observed for an association between the number of intronic junctions with the incidence of high Gleason grade (≥ 8; $P = 0.08$; n=4/50) and metastasis ($P = 0.16$; n=8/51), it did not reach statistical significance. Within BrCa, the number of intronic junctions correlated positively with the number of WGS-detected genomic breakpoints (Spearman correlation: $\rho=0.71$, $P = 2.2 \times 10^{-16}$, **Figure S12**). Because of the relative low number of junctions per sample combined with low number of colorectal cancer samples, further in-depth analysis on its recurrent events was not performed.

In the CGGA, BASIS and NGS-ProToCol datasets approximately 65% of all intronic and exonic junctions have both sides located within an annotated gene (**Figure 6**). Inversely, approximately 35% of the junctions have at least one side located within an intergenic region, regions that are often dismissed *a priori* by classical fusion gene detection tools [19, 20]. We found transcripts that incorporated cryptic (unannotated) exons, both intergenic as intronic (including anti-sense). For instance, a BrCa sample harboured intergenic junctions in *SDC4* transcripts using 5 consecutive cryptic exons (**Figure S13**). In contrast, a PCa sample had an intergenic rearrangement lacking mRNA level transcripts, thus only visible by the presence of pre-mRNA (**Figure S14**).

Genes associated with peaks in breakpoints

There were multiple, cancer type-specific, hotspots of junctions located near known oncogenes (**Figure 4**) such as *KIT*, *PDGFRA*, *EGFR*, *CDK4*, *MDM2* (glioma), *TMPRSS2*, *ERG* (PCa), *FGFR1* and *CCDN1* (BrCa). Enrichment analysis was performed using HUGO symbols of genes recurrently hit per dataset, indicating the pathway "Transcriptional misregulation in cancer [KEGG:05202]" was significantly more frequently hit ($P = 1.6 \times 10^{-4}$) within PCa due to *TMPRSS2*, *ERG*, *ETV1*, *H3FA3*, *SLC45A3* and *ELK4*. Within BrCa, pathways ETF and E2F were significantly enriched ($P = 6.75 \times 10^{-10}$, $P = 2.8 \times 10^{-6}$) in ER+ BrCa and "Proteoglycans in cancer" in ER- BrCa ($P = 1.4 \times 10^{-5}$). Genes that were recurrently hit in glioma were found more often in pathways "Rap1 signaling pathway" ($P = 3.2 \times 10^{-4}$), "Glioma" ($P = 5.9 \times 10^{-3}$) and "Ras Signaling" ($P = 2.6 \times 10^{-3}$) (**Table S2**).

Large gene amplifications

Hotspot regions (20-30 Mb) enriched with RNA-seq detected junctions were observed in the BrCa (chr11) and glioma (chr12) datasets. These hotspots differed from focal events such as *TMPRSS2-ERG* in the sense that they were larger, had no consistent fusion-partners and often contained multiple hotspot junctions per sample. If these hotspot region rearrangements are responsible for consistent changes at transcriptional level, they may provide a selective advantage. In both the BrCa and glioma datasets, transcriptional effects of the hotspot rearrangements were investigated by performing differential gene expression analysis between samples with (BrCa: chr11, glioma: chr12) and without a hotspot rearrangement (BrCa: n=122/283; glioma: n=45/274, respectively).

BrCa samples having a chr11 hotspot rearrangement were characterized by a large stretch of significant up-regulated genes within the respective hotspot region (**Figures 7A-C,S15**). The large genes *SHANK2* and *TENM4*, both located in the hotspot region, were the most frequently hit genes (25 and 13 samples, respectively), yet were not among the strongest up-regulated genes of the overall region. Instead, genes with a strong increase in LogFC were *FGF4* and *CCND1*, the cluster *KCTD21*, *ALG8* & *GAB2* and genes downstream of *TENM4*. Up-regulation of the overall region indicated amplifications of *CCND1* and/or the gene cluster, which is in concordance with previous reports [33]. We presume that selection of breakpoints near *SHANK2* is influenced by being adjacent to *CTTN*, a gene containing an enhancer often co-amplified with *CCND1* [34]. The high frequency of junctions in the relatively large, yet not heavily up-regulated *SHANK2* (785 kb) and *TENM4* (788 kb), suggests they are 'collateral damage' of the amplifications; a hypothesis that has been described in glioma [35]. This hypothesis is further supported by the lack of consistent fusion partners, consistency in acting as acceptor or donor and the absence of a clear spike in cumulative breakpoints (**Figure 7A-B**; **Table S3**).

Glioma samples having a junction harbouring the chr12 hotspot region (**Figure 7D-F**) were analysed similarly and also showed up-regulation of genes in the hotspot locus, with an increased LogFC of *CDK4*, *MDM2* and neighbouring genes. Both

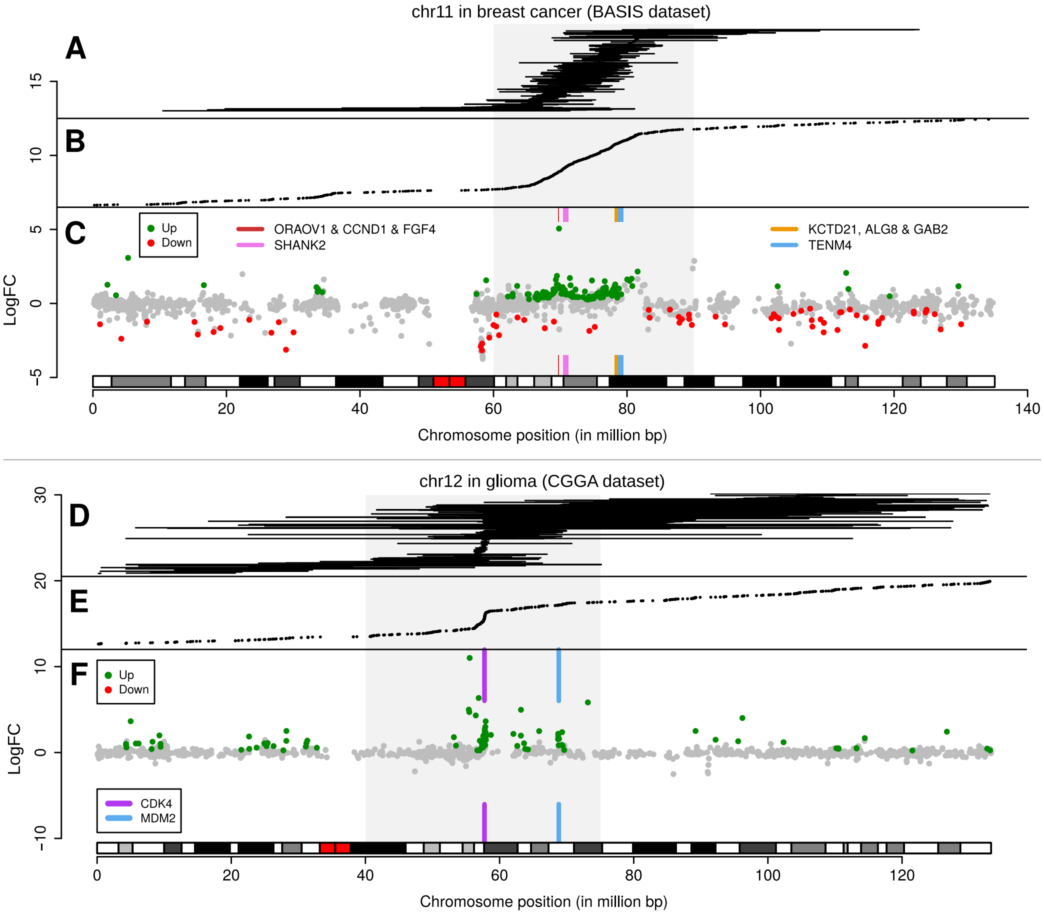

Figure 7. Differential gene expression in junction hotspot regions. (**A-C**) Overview of chr11 junctions, junction positions and hotspot associated differential gene expression in BrCa, using RNA-seq data only. (**A**) Intrachromosomal junctions not marked as putative circRNA, indicated by horizontal lines. (**B**) Junction end positions from intronic and exonic, inter- and intrachromosomal junctions not marked as putative circRNA. (**C**) Chromosomal differential expression plot for locus chr11:60,000,000-90,000,000 (grey square) with a q-value threshold of 0.001. Genes with the highest number of rearrangements, *SHANK2* and *TENM4*, are illustrated with coloured boxes. Peaks in LogFC were observed surrounding *ORAOV1*, *CCND1* & *FGF4* and surrounding *TENM4*. (**D-F**) Overview of chr12 junctions, junction positions and hotspot associated differential gene expression in glioma. (**D**) Intrachromosomal junctions not marked as putative circRNA are indicated with lines. (**E**) Junction end positions from intronic and exonic, inter- and intrachromosomal junctions not marked as putative circRNA. The junction enriched region chr12:40,000,000-75,000,000 is indicated with a grey square. (**F**) Chromosomal differential expression plot for locus chr12:40,000,000-75,000,000 with a q-value threshold of 0.01. Peaks in LogFC from up-regulated genes are found near *CDK4* and *MDM2*.

CDK4 and *MDM2* are known to be hyper-amplified in glioblastoma [36], often by double minute chromosomes [37]. The junctions showed a sharp increase in close proximity of *CDK4* (**Figure 7D-E**), likely indicating a common start of the amplification event. These breakpoints and up-regulated genes ceased just prior to *LRIG3*. Similarly, glioma samples harbouring rearrangements near the commonly hyper-amplified *EGFR* showed up-regulation of the surrounding locus (**Figure S16**).

Using RNA-seq data only, genomic rearrangements can be identified which can thereafter be used to reveal associated over-expression of oncogenes which have resulted from high copy gene amplifications.

Chromothripsis

In VCaP, the q-arm of chr5 has been subjected to chromothripsis as revealed by 468 intrachromosomal WGS-detected breakpoints [24]. Seventeen intronic and exonic junctions were identified in rRNA-minus RNA-seq, thus evidence for chromothripsis events was identified at (pre-)mRNA level (**Figure S17**). In three BrCa samples, high numbers of WGS-detected genomic breakpoints were identified on the q-arm of chr17. RNA-seq analyses revealed intronic and exonic junctions concordant with WGS data, which recurrently involved the genes *BCAS3*, *APPBP2*, *MED13*, *USP32* and *VMP1* (**Figure S18**). Taken together, this demonstrates the possibility to observe chromothripsis derived junctions in RNA-seq.

TMPRSS2-ERG

From previous analyses it emerged that *TMPRSS2-ERG* was the most prominent focal event identified. Therefore, we leveraged NGS-ProToCol prostate to study this fusion in detail. *TMPRSS2-ERG* is a highly prevalent fusion gene in prostate cancer (~50% of the diagnosed patients) [4], resulting in *TMPRSS2* driven up-regulation of *ERG*. In 32 of the 51 samples Dr. Disco identified mRNA fusion-transcripts of *TMPRSS2-ERG*, including genomic breakpoints in 27/32 samples (**Figure 8**). These fusions were in concordance with high *ERG* expression in those samples exclusively. The detection rate for genomic breakpoints for this oncogenenic fusion gene is thus markedly higher than for the overall number of genomic breakpoints. The genomic breakpoint did not pass filtering in sample 072, was marked exonic in sample 027 and was merged with closely adjacent (<450 bp; insert size) exonic junctions in three samples (053, 050 and 065); indicating that breakpoint-spanning reads were present in all 32 *TMPRSS2-ERG* positive RNA-seq samples.

Three samples had their *ERG*-flanking genomic breakpoint located in an intergenic region upstream to *ERG*'s first exon (**Figures S19-S20**). In two of these three sam-

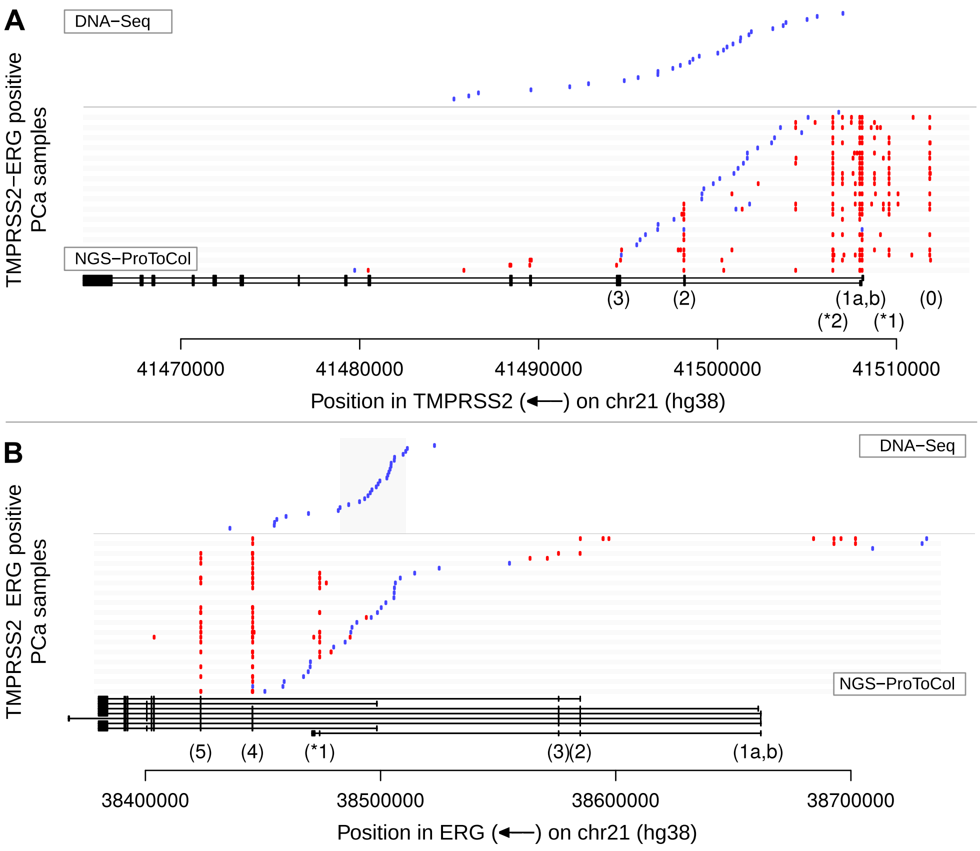

Figure 8. *TMPRSS2-ERG* junction map. *TMPRSS2* and *ERG* loci with endpoints of the junctions in NGS-ProToCol RNA-seq and non-matching targeted DNA-seq (Weier dataset). Gene structures are indicated at the bottom. Intronic junctions (representing genomic breakpoints) and genomic breakpoints (Weier dataset) are indicated in blue and exonic junctions in red. (**A**) For *TMPRSS2*, most breakpoints are detected after exon 1, up to exon 3. At mRNA level, apart from the first exons (1a and 1b), also exon 0 and exon 2 were commonly included in fusion transcripts. Two novel recurrent cryptic exons (*1 and *2) were common in fusion transcripts. (**B**) Three NGS-ProToCol samples (048, 054 & 075) have their genomic breakpoint before *ERG* and result in transcripts with additional, novel, intergenic cryptic exons.

ples, *TMPRSS2-ERG* fusion transcripts were identified containing cryptic intergenic exons (chr21:38,692,521-38,692,797 and chr21:38,701,593-38,701,947; hg38). In the same two samples, *ERG* had additional deletions, removing exon 2 (**Figure S19**).

The most abundant exonic junctions were T1-E4 and T1-E5 (**Figures 8,S20-S21**) which is in concordance with previous reports [38]. Genomic breakpoints were indeed located in hotspot-regions within the first two introns of *TMPRSS2* and the last half of *ERG* intron 3 [39]. Subsequent analysis of shallow sequenced FFPE RNA-seq samples revealed *TMPRSS2-ERG* in 181 samples (**Figures S20-S21**) and confirmed this remarkable breakpoint preference region within *ERG* intron-3 more precisely.

Two novel exons in *TMPRSS2* were observed in both fusion and wild-type transcripts (**Figure 8**). These cryptic exons were both lowly expressed as they represented 3% of all *TMPRSS2-ERG* reads in samples having the splice variant. Furthermore, intergenic *TMPRSS2* exon-0 [40] was detected in fusion mRNA-transcripts within 18/32 *TMPRSS2-ERG* positive samples.

One sample contained an exonic junction originating in *ERG* and spanning to *TMPRSS2* in which the gene order and included exons indicated that this *ERG-TMPRSS2* fusion was caused by a reciprocal translocation instead of the common 3 Mb deletion (**Figure S22**). Other *TMPRSS2* related fusions were *TMPRSS2-RERE*, *SERINC5-TMPRSS2*, *TMPRSS2-TBX3*, *TMPRSS2-PADI4*, *MGA-TMPRSS2* and *TMPRSS2-CATSPER2* (**Table S4**).

VCaP

PCa cell line VCaP has been subjected to intensive research, revealing it is *TMPRSS2-ERG* positive and contains two additional related genomic rearrangements (breakpoint-A and breakpoint-B) [39, 24]. *TMPRSS2-ERG* in VCaP was analysed using both rRNA-minus and poly(A)$^+$ RNA-seq data.

Poly(A)$^+$ RNA-seq shows that only the first exon of *TMPRSS2* splices to *ERG*, even though the genomic breakpoint to *ERG* is located in the 5th intron (**Figure S23A**). The rRNA-minus data not only confirms this splice junction but also reveals all *TMPRSS2* and *ERG* spanning genomic breakpoints, concordant with the WGS results. Moreover, read stranding indicates that a region containing the 4th and 5th exon is inverted, and that its breakpoint-A is an inversion. Breakpoint-B is an amplification and the junction from *TMPRSS2* to *ERG* is again inverted such that *ERG* is transcribed in its original orientation. The junction from *TMPRSS2* to *ERG* deletes the genomic region containing *ERG*s exons 2 and 3. Thus, only *TMPRSS2* exon 1 splices to *ERG* since exon 2 and 3 are deleted and exon 4 and 5 are inverted (**Figure S23B**). The small proportion of reads still present within the deleted *TMPRSS2* exons 2 and 3 in the rRNA-minus data originate from the non-fusion allele(s). The rRNA-minus RNA-seq data not only revealed both intronic and exonic junctions but also showed the complex downstream effects on transcription and splicing.

CircRNA detection

Head-to-tail aligned reads (**Figure S24**) are marked as chimeric (discordant) by STAR and are used as input for our method. Such reads are not only observed in transcripts from genomic tandem duplications, but also from circular mi-

tochondrial DNA and circular RNAs. Using the PCa-LINES rRNA-minus samples, we found that 88.6% of the junctions with a head-to-tail orientation were located exactly on exon-junctions corresponding to annotated circRNAs from circBase v31 (**Figure S25**).

Discussion

RNA-seq is generally performed on poly(A)$^+$ RNA-seq and fusion gene detection algorithms are in particular focused on annotated exons or splice junctions. It has become common practice to sequence ribosome-depleted total RNA (rRNA-minus) [12], especially for partially degraded (FFPE) RNA samples. rRNA-minus RNA-seq is interesting as it yields also non-polyadenylated transcripts, including pre-mRNA-derived intronic sequences. As a result, there is more genomic coverage in rRNA-minus RNA-seq alignments compared to poly(A)$^+$ RNA-seq (**Figure S26**), which provides more opportunity to reveal fusion transcripts and a broader understanding of the transcriptome. Because genomic breakpoints are often harboured within introns [6] and intergenic regions (**Figure S27**), we interrogated to what extend rRNA-minus RNA-seq can be used to reveal genomic breakpoints as this also captures intronic (pre-mRNA) reads. Addressing this required to analyse the genome without regional restrictions.

Here, we confirm by utilising Dr. Disco, that RNA-seq data can be used to reveal genomic breakpoints of expressed transcripts in an automated fashion. Detection was limited to approximately 7% of WGS detected breakpoints but markedly higher for the driver *TMPRSS2-ERG* fusion gene (85% detected; 100% presence). As the algorithm was conservative in detecting mRNA fusions, it is likely that genomic breakpoints were missed and the actual percentage is somewhat higher. Conversely, estimation of this percentage implies that WGS results offer the ground truth but these are also affected by noise, coverage and filter cut-offs, indicating this percentage is an approximation. The results commonly included intergenic junctions. For instance, three *TMPRSS2-ERG* fusions had their breakpoint located before *ERG*, supported by cryptic intergenic splice junctions and intergenic pre-mRNA coverage (**Figure S19**). Furthermore, intronic intergenic junctions in chromothripsis regions in three BrCa samples were in 14/18 cases validated by WGS junctions (**Figure S18A**).

That both Arriba and Dr. Disco make use of STAR and that Arriba finds a higher number of true positives indicates that improving the filtering is an important future step, but care must be taken not to compromise Dr. Disco's true positive ratio. The large search space combined with graph analysis was an effective solution as shown by providing an unique view on 1,275 transcriptomes. Whereas our initial hypothesis was that only rRNA-minus RNA-seq would reveal genomic breakpoints, this also accounts for poly(A)$^+$. In poly(A)$^+$ RNA genomic breakpoints are observed less frequently, with lower confidence and often in long UTRs. The VCaP *TMPRSS2-ERG* analysis underlined the differences: on the basis of rRNA-minus RNA-seq, each genomic rearrangement with resulting splice variants and their strand as well as the order of events could be deduced, while both the poly(A)$^+$ and WGS alone were insufficient.

CircRNAs are a relatively new group of non-polyadenylated transcripts with more than 90,000 different human circRNAs identified so far [41, 42]. The distinctive signature of proximate exonic head-to-tail junctions sets them apart from other junctions, except for small tandem duplications. A useful addition to the algorithm could be annotation of the junctions using a circRNA database such as circBase [41]. The proposed method is not specifically designed to identify circRNAs as it has stringent cut-off levels, merges splice variants into subgraphs and requires more than 1 read. The number of circRNAs identified is therefore lower as compared with dedicated detection tools such as CIRI [43, 44].

The number of RNA-seq detected intronic junctions representing genomic breakpoints varied largely between the four different cancer types (PCa, BrCa, CRC and glioma). This variation is in line with the omics-reported number of structural variants; low in colorectal cancer [45] while high in breast cancer [46, 47], but these differences are confounded by the influence of sequencing depth, length and library preparation which vary per dataset.

While only a fraction of all genomic rearrangements is transcribed, an even smaller fraction is causal for fusion genes which are currently of high interest in RNA research. Transcribed genomic breakpoints more often involve driver events than non-transcribed genomic breakpoints, as seen with *TMPRSS2-ERG*. Known exceptions that can be considered driver events include promoter and enhancer rearrangements such as known for *AR* and *FOXP1* [48], but also tumour suppressor gene deletions [34, 49]. The reasoning that RNA-seq is not a replacement for WGS is valid. However, we show that by also looking at intronic and intergenic regions, more cancer-specific transcripts are identified and context is provided than being restricted to classical mRNA fusion genes only.

Although WGS depth surpasses 40x coverage, Dr. Disco showed that 26% and 48% of all RNA-seq intronic junctions in PCa and BrCa, respectively, were not identified by WGS. While these will contain false positives for sure, 100 of the 357 were found in 8 of the 207 samples all characterized by high number of genomic breakpoints. We suspect this discrepancy is partially due to actual genomic breakpoints missed by WGS because of: high RNA-seq coverage of highly expressed genes (up-to 1000x), clonality as this difference was in particular high for a small subset of samples, low local coverage in WGS, and selection criteria in software such as cut-offs and read mapping rulings. In the PCMM-FFPE dataset, samples with low insert sizes or short read lengths often resulted in insufficient split-reads whilst resulting in many false positive read-pairs in full transcriptome analysis. However, it could still be used effectively in identifying the targeted, highly expressed, *TMPRSS2-ERG* fusion events.

The large number of PCa samples allowed to analyse *TMPRSS2-ERG* in-depth, revealing: additional cryptic and intergenic exons including *TMPRSS2* exon-0 [40], a detailed map of the genomic breakpoints, genomic breakpoints located before *ERG* that combined with cryptic intergenic exons co-exist with exon 2 deletions and a tumour that harbours *TMPRSS2-ERG* which originated from a reciprocal translocation rather than a deletion. In VCaP, stranded RNA-seq provided an advantage in deciphering the chronological order of complex genomic events. Moreover, this underlined the importance of automatic resolution of complex genomic rearrangements or poly-fusions. The current implementation does not offer such top-level integration for poly-fusions while techniques exist with this purpose in mind [7, 50]. Integration of such techniques prompts future work. The current algorithm uses discordant reads exclusively. It would be interesting to investigate the added value of extending the detection with regions enriched with concordant opposite stranded reads, to strengthen detection of junctions having insufficient coverage of discordant reads.

In both BrCa and glioma, RNA-seq data alone revealed hotspot regions of junctions with subsequent up-regulation of known amplified oncogenes and neighbouring genes within these regions. The inconsistent transcriptional direction of the junctions combined with the lack of consistent acceptor/donor genes provides additional context that distinguishes these events from focal fusion genes. In BrCa, this combined analysis indicated that the events are related to *CCND1* ampli-

fications, despite the frequent events in sizeable genes *TEMN4* and *SHANK2* of which their fusion transcripts are not driving cancer.

Chromothripsis derived junctions matching WGS detected genomic breakpoints were present at RNA level, in VCaP and three BrCa samples. As with most genomic rearrangements, the majority of the chromothripsis rearrangements were not detected on RNA level. Solely based on RNA-seq data, it will be difficult to prove presence of chromothripsis as not all parameters that define this specific process can readily be extracted (e.g. copy-number variations, short insertions, loss of heterozygosity) [51, 52]. However, potential indicators for oncogenic chromothripsis events can be present in RNA.

While our preliminary aim was to study to which extent genomic breakpoints are present in RNA, we were surprised by how common both intergenic events and cryptic exons are. That approximately 35% of the junctions in rRNA-minus datasets were full or partial intergenic events is an under-representation because, as for example with *TMPRSS2-ERG* exon-0, intergenic splice variants are merged with gene-spanning splice variants and will be considered in-gene as-whole. Intergenic mRNA fusions are characterized by incorporation of (typically multiple) cryptic exons. But cryptic exons are not limited to intergenic events, as we reported that cryptic exons in fusion transcripts transcribed in the anti-sense direction of a gene are common.

Cryptic exons are of importance as they may encode nonsense proteins with completely novel neo-antigens that are more divergent than point mutation-based neo-antigens and could therefore be more immunogenic [53]. We want to emphasize the importance of fusions beyond those incorporating annotated exons, because we show they can be transcribed into stable mRNA, thereafter be translated into protein and potentially be oncogenic and/or immunogenic. This, deciphering the consequence of rearrangements, annotation of cryptic exons and their coding potential for nonsense protein sequences is relevant for therapeutic interventions using tumour-specific antigens [54].

Conclusion

Facilitated by Dr. Disco, we set out to extract both intronic and exonic junctions from comprehensive rRNA-minus RNA-seq datasets and identified novel genomic breakpoints, circRNAs, novel gene and intergenic fusions, cryptic exons, chromothripsis events and were able to link expressed rearrangements to transcriptional outcome. Discovering both genomic breakpoints and exonic junctions from only RNA-seq data requires an analysis strategy keeping these two levels of information separated. The number of breakpoints detected is limited to ~7%, as most are not within expressed regions. These results indicate that this analysis is not a replacement for WGS, but performing analysis like this will result in considerably more cancer specific transcripts than by interrogating classical fusion-genes only. This holds in particular for rRNA-minus RNA-seq, which harbours more intronic reads. Furthermore, combined WGS and RNA analysis showed that RNA can function as informative supplement to WGS analysis because of stranding, expression and resolution of the fusion gene structure(s). rRNA-minus RNA-seq provides more unique and complete information on non-polyadenylated and aberrant transcripts and, if the pre-mRNA is sequenced, the genomic breakpoints that underlie transcriptional changes.

Thus, RNA-seq data can reveal genomic breakpoints, (cryptic and/or intergenic) splicing and gene expression information, which together can reveal consequences and their selective advantage for cancer development and progression and be a useful supplement to DNA-seq.

Methods

Sequencing and datasets

The used sequencing datasets are given in **Table 1**. For NGS-ProToCol and the rRNA-minus RNA-seq of PCa-LINES, RNA was extracted using RNA-Bee (Campro Scientific, Berlin, Germany) and the library prepared for RNA-seq used the NEBNext Ultra Directional RNA Library Prep Kit for Illumina with rRNA reduction. The sample preparation was performed according to the protocol 'NEBNext Ultra Directional RNA Library Prep Kit for Illumina' (NEB, Cat. #E7420S/L and E6310S/L/X). Briefly, rRNA was reduced using RNase H-based method. Then, fragmentation of the rRNA reduced RNA and a cDNA synthesis was performed. This was used for ligation with the sequencing adapters and PCR amplification of the resulting product. The quality and yield after sample preparation were measured with the Fragment Analyzer (Advanced Analytical). Clustering and DNA sequencing using the Illumina cBot and HiSeq 2500 was performed according to manufacturer's protocols. A concentration of 16.0 pM of DNA was used as input. HiSeq control software HCS (v2.2.58) was used. Image analysis, base calling, and quality check was performed with the Illumina data analysis pipeline RTA (v1.18.64) and Bcl2fastq (v2.17). The 126 bp stranded Illumina HiSeq 2500 paired-end reads have a peak in fragment size of 300-600 bp and the samples have an average depth of 70 million paired-end reads.

Of the PCa-LINES samples, each samples was WGS DNA sequenced and processed using the Complete Genomics platform [24, 57]. The matching poly(A)+ RNA-seq samples were taken from the TraIT-Cell Line Use Case study [58, 55]. rRNA-minus RNA-seq sample G-110 was not sequenced within PCa-LINEs but sequenced in NGS-ProToCol as sample 7046-004-052.

In the BASIS RNA-seq dataset [26], total RNA was extracted and cleaned from abundant RNAs such as rRNA and tRNA as described elsewhere [27]. The BASIS DNA-seq data preparation and analysis is described elsewhere [25] and coordinates were converted to hg38 using pyliftover (v0.4) where needed.

The detection of genomic breakpoints from additional *TMPRSS2-ERG* fusions determined by targeted DNA-seq was described elsewhere [39] and genomic coordinates were obtained from this study accordingly. Genomic breakpoints of *TMPRSS2-ERG* and chromothripsis on chr5 in VCaP were described elsewhere [39, 24]. Predicted CMS classes for NGS-ProToCol colon samples were described elsewhere [22]. CGGA metadata was described elsewhere [28].

Computational analysis

RNA-seq data was aligned with STAR [59] (v2.4.2) using fusion settings and hg38 as reference genome. More details are given in **Supplementary Methods**. Dr. Disco (v0.17.8[1]) was used for analysis. Arriba [31] (v2.1.0[2]) was used for evaluation analysis. For **Figure S26**, we designed and used our free software package to generate Lorenz and coverage plots and statistics: https://github.com/yhoogstrate/bam-lorenz-coverage (v2.3.0). Processed bam files used to estimate general genome coverage statistics were obtained from EGAS00000000052 [56]. Pathway enrichment was performed with g:Profiler web: https://biit.cs.ut.ee/gprofiler/gost [60], using gene identifiers as non-ordered query. For differential expression analysis, the

1 git commit 2a9ff32950b71029b124ff4d16544b2953c57dbe
2 git commit 3492d2c28917fe6c9320b1caab73afbb93f7bfbf

Table 1. Datasets overview.

Dataset	Tissue	Sequencing	n	Depth	Ref	Ref(s)	Comments
NGS-ProToCol	Prostate Cancer	rRNA– RNA S	41	~70M	EGAS00001002816	[42]	
NGS-ProToCol	Normal Prostate	rRNA– RNA S	51	~70M	EGAS00001002816	[42]	
NGS-ProToCol	Colon Cancer	rRNA– RNA S	30	~70M	EGAS00001002854	[21, 42, 22]	
NGS-ProToCol	Colon Adenoma	rRNA– RNA S	30	~70M	EGAS00001002854	[21, 42, 22]	
NGS-ProToCol	Normal Colon	rRNA– RNA S	18	~70M	EGAS00001002854	[21, 42, 22]	
BASIS	Breast Cancer	rRNA– RNA S	289	~150M	EGAS00001001178	[26, 27]	
PCa-LINES	Prostate Cancer	rRNA– RNA S	6 (+1)*	~37M	EGAS00001001476	-	NGS-ProToCol 7046-004-052 matches G-110
PCa-LINES	Prostate Cancer	poly(A)$^+$ U	7	~50M	EGAS00001001476	[55]	
PCMM-FFPE	Prostate Cancer	rRNA– RNA S	529	~40M			shallow FFPE
CGGA	Glioma	rRNA– RNA U	274	~30M	GSE48865	[28]	
ENCODE MCF-7	Breast Cancer	poly(A)$^+$ S	1	~138M	SRR534293	[29, 20]	
BASIS	Breast Cancer	WGS DNA	560	~40X	EGAS00001001178	[25]	
PCa-LINES	Prostate Cancer	WGS DNA	7	~100X	EGAS00001001476	[24]	
Weier	Prostate Cancer	TMPRSS2-ERG targeted DNA	29			[39]	no raw data was used
Pleasance	Melanoma	WGS DNA	9	~40X	EGAS00000000052	[56]	for determining WGS coverage

RNA sequencing datasets are given a suffix indicating whether they were performed Stranded or Unstranded, rRNA– is a substitute for rRNA-minus.

annotation of the results of Dr. Disco and further integration with gene sets for determining intergenic status, Ensembl 89 was used.

Plots were made with: base R (3.6.3), ggplot2 (3.3.5), plotrix (3.8.1) and circlize (0.4.13) and illustrations with Inkscape (0.92.4). Differential gene expression analysis was performed using edgeR (3.28.1) [61]. Associations between the frequency of breakpoints per sample and clinical parameters were tested using the Mann Whitney U test in R.

For the Venn diagrams describing overlap across intronic, exonic and WGS junctions (**Figure 3A**), both sides of the junctions must be within 40 genomic nucleotides in proximity to be considered a match. Head-to-tail junctions and junctions to alternate loci were excluded. For the comparison of BrCa WGS and rRNA-minus results (**Figure 4**), interchromosomal entries were compared to avoid an unfair comparison due to: (i) small WGS indels detected on the basis of non split reads and (ii) transcripts unrelated to genomic rearrangements such as read-throughs or circRNAs.

Chromosomal differential expression plots (**Figure 8**) were made using base R. For a given locus and q-value threshold, a cohort is separated in a mutant and wild-type group by having one or more intronic or exonic junctions within the given locus. Differential expression analysis is performed across these groups using edgeR. Every gene located on the chromosome on which the locus is located, is plotted with its genomic center as defined by Ensembl 89 on the x-axis and with edgeR's LogFC on the y-axis. A gene that is up-regulated in the mutant group has a positive logFC change and a gene that is down regulated a negative logFC. When the gene is not significantly differentially expressed across the wildtype and mutant group (q-value below predetermined threshold) the gene will be coloured grey. If the difference is significant, it will be coloured green (up) or red (down).

Snapshots of discordant alignments were made in IGV's (2.8.0) using the split view and with *Color alignments by* set to *read group*.

Data Availability

Availability of source code and requirements (optional, if code is present)

Lists the following:

- Project name: Dr. Disco
- Project home page: https://github.com/yhoogstrate/dr-disco
- bio.tools: https://bio.tools/dr_disco
- SciCrunch: SCR_021739
- Operating system(s): GNU/Linux
- Programming language: Python
- Other requirements: STAR (aligner)
- License: GNU GPL 3.0

Availability of supporting data and materials

Concatenated Dr. Disco v.0.17.8 results are available at: https://doi.org/10.5281/zenodo.4159414. The results of the evaluation on the ENCODE MCF-7 dataset are given as **Supplementary Table S1**.

Additional Files

Additional file 1 - Dr. Disco Technical Specification - Supplementary Material.docx
Additional file 2 - Supplementary Figures - Supplementary Material.pptx

- **Supplementary Figure S1**: Exonic and intronic junctions
- **Supplementary Figure S2**: Snapshot of fusion using cryptic exons in ENCODE MCF-7 dataset
- **Supplementary Figure S3**: IGV Screenshot of in-exon genomic breakpoint
- **Supplementary Figure S4**: Junctions in PCa-LINES dataset
- **Supplementary Figure S5**: rRNA-minus RNA-seq and WGS data intersection BASIS dataset

- **Supplementary Figure S6**: Correlation binned density WGS breakpoints and rRNA-minus junctions
- **Supplementary Figure S7**: Intrachromosomal junctions in BASIS dataset
- **Supplementary Figure S8**: Intrerchromosomal junctions in BASIS dataset
- **Supplementary Figure S9**: Intrachromosomal junctions in NGS-ProToCol prostate dataset
- **Supplementary Figure S10**: Intrachromosomal junctions in NGS-ProToCol colon dataset
- **Supplementary Figure S11**: Intrachromosomal junctions in CGGA dataset
- **Supplementary Figure S12**: Correlation RNA-seq depth, WGS breakpoints and RNA-seq junctions in BASIS dataset
- **Supplementary Figure S13**: Snapshot cryptic fusion in BrCa sample PR9608a
- **Supplementary Figure S14**: Snapshot of intergenic breakpoints in PCa sample 7046-004-134
- **Supplementary Figure S15**: Chromosomal differential expression plot of chr11 in BASIS dataset
- **Supplementary Figure S16**: Chromosomal differential expression plot of *EGFR* in CGGA dataset
- **Supplementary Figure S17**: Chromothripsis on chr5q in VCaP
- **Supplementary Figure S18**: Catastrophic events on chr17 in BASIS dataset
- **Supplementary Figure S19**: Snapshot of intergenic breakpoints in *TMPRSS2-ERG*
- **Supplementary Figure S20**: Detailed overview of junctions in *ERG*
- **Supplementary Figure S21**: Detailed overview of junctions in *TMPRSS2*
- **Supplementary Figure S22**: Schematic representation of *TMPRSS2-ERG* as reciprocal translocation
- **Supplementary Figure S23**: *TMPRSS2-ERG* in VCaP
- **Supplementary Figure S24**: Schematic overview discordant read orientations
- **Supplementary Figure S25**: Overlap head-to-tail junctions and circBase in PCa-LINES dataset
- **Supplementary Figure S26**: Lorenz and coverage plots of rRNA-minus, poly(A)+ and WGS data
- **Supplementary Figure S27**: (Inter)genic events in WGS

Additional file 3 – Supplementary Methods – Supplementary Material.docx
Additional file 4 – Table S1 – Results SRR534293 – Supplementary Material.xlsx Additional file 5 – Table S2 – gProfiler – Supplementary Material.xlsx
Additional file 6 – Table S3 – SHANK2 and TENM4 related junctions – Supplementary Material.xlsx
Additional file 7 – Table S4 – TMPRSS2-ERG and related junctions – Supplementary Material.xlsx

Declarations

List of abbreviations

BrCa: breast cancer; **FFPE**: formalin-fixed paraffin-embedded; **logFC**: logarithmic fold chance; **mRNA**: messenger RNA (5′ capped, polyadenylated and spliced); **PCa**: prostate cancer; **poly(A)$^+$**: polyadenylated; **pre-mRNA**: RNA that is actively being transcribed by polymerase (not polyadenylated); **rRNA-minus RNA-seq**: RNA-seq prepared such that there is no specific positive selection for poly(A)-tails while reducing the amount of ribosomal RNA ; **poly(A)$^+$ RNA-seq**: RNA-seq prepared with a positive selection for poly(A)-tails ; **WGS**: whole genome sequencing

Ethical Approval (optional)

For the CTMM NGS-ProToCol study (NGS-ProToCol, Next Generation Sequencing from Prostate to Colorectal Cancer – Center for Translational Molecular Medicine (2014-2015); https://www.lygature.org/ctmm-portfolio), 51 prostate cancers from the Erasmus MC were snap-frozen and stored in liquid nitrogen as previously described [62]. Use of the samples for research purposes was approved by the Erasmus MC Medical Ethics Committee according to the Medical Research Involving Human Subjects Act (MEC-2004-261; MEC-2010-176).

Consent for publication

Not applicable

Competing Interests

The authors declare that they have no competing interests.

Funding

This study was performed within the framework of the CTMM (Center for Translational Molecular Medicine) research program; NGS-ProToCol [grant 03O-402]; PCMM [grant 03O-203-1]; Translational Research IT (TraIT); the Complete Genomics Inc. grant [EMC GL 083111]; the FP7 Marie Curie Initial Training Network PRO-NEST [grant number 238278] and Support for the Cancer Computational Biology Center was provided by the Daniel den Hoed Foundation. Funding for open access charge: NGS-ProToCol [grant 03O-402].

Author's Contributions

Y.H. and G.J. designed most of the experiments. Y.H. carried out most of the experiments, analysis and wrote the manuscript and software. M.K., R.B., J.v.R., H.v.d.W., A.S. and G.J. contributed to the methodology. N.D., D.S., D.v.d.M., F.P., C.B, G.v.L., M.S., J.M., W.v.W., B.J., E.C., M.d.J., G.M., R.F and G.J. acquired data. H.v.d.W, J.v.R, S.v.L, C.R., R.B, M.S., A.S., M.K. analysed and prepared data. P.F., R.B., M.S., G.J., B.J., R.F. and P.v.d.S. made large contributions to writing the manuscript. G.J., A.S., P.F., C.B., R.F. and P.v.d.S. acquired funding.

Acknowledgements

Not applicable

References

1. Weinhold N, Jacobsen A, Schultz N, Sander C, Lee W. Genome-wide analysis of noncoding regulatory mutations in cancer. Nature Genetics 2014;46(11):1160–1165.
2. Calabrese C, Davidson NR, Demircioğlu D, Fonseca NA, He Y, Kahles A, et al. Genomic basis for RNA alterations in cancer. Nature 2020;578(7793):129–136.
3. Li Y, Roberts ND, Wala JA, Shapira O, Schumacher SE, Kumar K, et al. Patterns of somatic structural variation in human cancer genomes. Nature 2020;578(7793):112–121.
4. Tomlins SA, Rhodes DR, Perner S, Dhanasekaran SM, Mehra R, Sun XW, et al. Recurrent fusion of TMPRSS2 and

ETS transcription factor genes in prostate cancer. Science (New York, NY) 2005 oct;310(5748):644–8.
5. Burmeister T, Schwartz S, Bartram CR, Gökbuget N, Hoelzer D, Thiel E. Patients' age and BCR-ABL frequency in adult B-precursor ALL: A retrospective analysis from the GMALL study group. Blood 2008;112(3):918–919.
6. Annala MJ, Parker BC, Zhang W, Nykter M. Fusion genes and their discovery using high throughput sequencing. Cancer Letters 2013;340(2):192–200.
7. McPherson A, Wu C, Wyatt AW, Shah S, Collins C, Sahinalp SC. NFuse: Discovery of complex genomic rearrangements in cancer using high-throughput sequencing. Genome Research 2012;22(11):2250–2261.
8. Zhang J, White NM, Schmidt HK, Fulton RS, Tomlinson C, Warren WC, et al. INTEGRATE: Gene fusion discovery using whole genome and transcriptome data. Genome Research 2016;26(1):108–118.
9. Zhao S, Zhang Y, Gamini R, Zhang B, Von Schack D. Evaluation of two main RNA-seq approaches for gene quantification in clinical RNA sequencing: PolyA+ selection versus rRNA depletion. Scientific Reports 2018;8(1):4781.
10. Erdem-Eraslan L, Van Den Bent MJ, Hoogstrate Y, Naz-Khan H, Stubbs A, Van Der Spek P, et al. Identification of patients with recurrent glioblastoma who may benefit from combined bevacizumab and CCNU Therapy: A Report from the BELOB Trial. Cancer Research 2016;76(3):525–534.
11. Taggart AJ, Fairbrother WG. ShapeShifter: a novel approach for identifying and quantifying stable lariat intronic species in RNAseq data. Quantitative Biology 2018;.
12. Zhao W, He X, Hoadley KA, Parker JS, Hayes DN, Perou CM. Comparison of RNA-Seq by poly (A) capture, ribosomal RNA depletion, and DNA microarray for expression profiling. BMC Genomics 2014;15(1):419.
13. Heyer EE, Deveson IW, Wooi D, Selinger CI, Lyons RJ, Hayes VM, et al. Diagnosis of fusion genes using targeted RNA sequencing. Nature Communications 2019;10(1).
14. Edgren H, Murumagi A, Kangaspeska S, Nicorici D, Hongisto V, Kleivi K, et al. Identification of fusion genes in breast cancer by paired-end RNA-sequencing. Genome Biology 2011 jan;12(1):R6.
15. Liu S, Tsai WH, Ding Y, Chen R, Fang Z, Huo Z, et al. Comprehensive evaluation of fusion transcript detection algorithms and a meta-caller to combine top performing methods in paired-end RNA-seq data. Nucleic Acids Research 2015;44(5):e47.
16. Kim D, Salzberg SL. TopHat-Fusion: an algorithm for discovery of novel fusion transcripts. Genome biology 2011 jan;12(8):R72.
17. McPherson A, Wu C, Hajirasouliha I, Hormozdiari F, Hach F, Lapuk A, et al. Comrad: Detection of expressed rearrangements by integrated analysis of RNA-Seq and low coverage genome sequence data. Bioinformatics 2011;27(11):1481–1488.
18. Ge H, Liu K, Juan T, Fang F, Newman M, Hoeck W. FusionMap: Detecting fusion genes from next-generation sequencing data at base-pair resolution. Bioinformatics 2011;27(14):1922–1928.
19. Nicorici D, Satalan M, Edgren H, Kangaspeska S, Murumagi A, Kallioniemi O, et al. FusionCatcher - a tool for finding somatic fusion genes in paired-end RNA-sequencing data; 2014.
20. Davidson NM, Majewski IJ, Oshlack A. JAFFA: High sensitivity transcriptome-focused fusion gene detection. Genome Medicine 2015;7(1):43.
21. Komor MA, Bosch LJW, Bounova G, Bolijn AS, Delis-van Diemen PM, Rausch C, et al. Consensus molecular subtype classification of colorectal adenomas. Journal of Pathology 2018 nov;246(3):266–276. https://onlinelibrary.wiley.com/doi/10.1002/path.5129.
22. Komor MA, de Wit M, van den Berg J, Martens de Kemp SR, Delis-van Diemen PM, Bolijn AS, et al. Molecular characterization of colorectal adenomas reveals POFUT1 as a candidate driver of tumor progression. International Journal of Cancer 2020;146(7):1979–1992.
23. Korenchuk S, Lehr JE, McLean L, Lee YG, Whitney S, Vessella R, et al. VCaP, a cell-based model system of human prostate cancer. In Vivo 2001;15(2):163–168. http://www.ncbi.nlm.nih.gov/pubmed/11317522.
24. Teles Alves I, Hiltemann S, Hartjes T, Van Der Spek P, Stubbs A, Trapman J, et al. Gene fusions by chromothripsis of chromosome 5q in the VCaP prostate cancer cell line. Human Genetics 2013 jun;132(6):709–713.
25. Nik-Zainal S, Davies H, Staaf J, Ramakrishna M, Glodzik D, Zou X, et al. Landscape of somatic mutations in 560 breast cancer whole-genome sequences. Nature 2016;534(7605):47–54.
26. Smid M, Rodríguez-González FG, Sieuwerts AM, Salgado R, Prager-Van Der Smissen WJC, Vlugt-Daane MVD, et al. Breast cancer genome and transcriptome integration implicates specific mutational signatures with immune cell infiltration. Nature Communications 2016;7(1):1–9.
27. Smid M, Wilting SM, Uhr K, Rodríguez-González FG, De Weerd V, Prager-Van Der Smissen WJC, et al. The circular RNome of primary breast cancer. Genome Research 2019;29(3):356–366.
28. Bao ZS, Chen HM, Yang MY, Zhang CB, Yu K, Ye WL, et al. RNA-seq of 272 gliomas revealed a novel, recurrent PTPRZ1-MET fusion transcript in secondary glioblastomas. Genome Research 2014;24(11):1765–1773.
29. Dunham I, Kundaje A, Aldred SF, Collins PJ, Davis CA, Doyle F, et al. An integrated encyclopedia of DNA elements in the human genome. Nature 2012 sep;489(7414):57–74. http://www.nature.com/articles/nature11247.
30. McPherson A, Hormozdiari F, Zayed A, Giuliany R, Ha G, Sun MGF, et al. Defuse: An algorithm for gene fusion discovery in tumor rna-seq data. PLoS Computational Biology 2011;7(5).
31. Uhrig S, Ellermann J, Walther T, Burkhardt P, FrÃ¶hlich M, Hutter B, et al. Accurate and efficient detection of gene fusions from RNA sequencing data. Genome Research 2021 mar;31(3):448–460. http://genome.cshlp.org/lookup/doi/10.1101/gr.257246.119.
32. Bolisetty MT, Beemon KL. Splicing of internal large exons is defined by novel cis-acting sequence elements. Nucleic Acids Research 2012;40(18):9244–9254.
33. Elsheikh S, Green AR, Aleskandarany MA, Grainge M, Paish CE, Lambros MBK, et al. CCND1 amplification and cyclin D1 expression in breast cancer and their relation with proteomic subgroups and patient outcome. Breast Cancer Research and Treatment 2008;109(2):325–335.
34. Morton AR, Dogan-Artun N, Faber ZJ, MacLeod G, Bartels CF, Piazza MS, et al. Functional Enhancers Shape Extrachromosomal Oncogene Amplifications. Cell 2019;179(6):1330–1341.e13.
35. Nikolaev S, Santoni F, Garieri M, Makrythanasis P, Falconnet E, Guipponi M, et al. Extrachromosomal driver mutations in glioblastoma and low-grade glioma. Nature Communications 2014;5(1):5690.
36. Rollbrocker B, Waha A, Louis DN, Wiestler OD, Von Deimling A. Amplification of the cyclin-dependent kinase 4 (CDK4) gene is associated with high cdk4 protein levels in glioblastoma multiforme. Acta Neuropathologica 1996;92(1):70–74.
37. Decarvalho AC, Kim H, Poisson LM, Winn ME, Mueller C, Cherba D, et al. Discordant inheritance of chromosomal

and extrachromosomal DNA elements contributes to dynamic disease evolution in glioblastoma. Nature Genetics 2018;50(5):708–717.

38. Clark J, Merson S, Jhavar S, Flohr P, Edwards S, Foster CS, et al. Diversity of TMPRSS2-ERG fusion transcripts in the human prostate. Oncogene 2007;26(18):2667–2673.
39. Weier C, Haffner MC, Mosbruger T, Esopi DM, Hicks J, Zheng Q, et al. Nucleotide resolution analysis of TMPRSS2 and ERG rearrangements in prostate cancer. Journal of Pathology 2013;230(2):174–183.
40. Hermans KG, Boormans JL, Gasi D, Van Leenders GJHL, Jenster G, Verhagen PCMS, et al. Overexpression of prostate-specific TMPRSS2(exon 0)-ERG fusion transcripts corresponds with favorable prognosis of prostate cancer. Clinical Cancer Research 2009;15(20):6398–6403.
41. Glažar P, Papavasileiou P, Rajewsky N. CircBase: A database for circular RNAs. Rna 2014;20(11):1666–1670.
42. Chen S, Huang V, Xu X, Livingstone J, Soares F, Jeon J, et al. Widespread and Functional RNA Circularization in Localized Prostate Cancer. Cell 2019;176(4):831–843.e22.
43. Zeng X, Lin W, Guo M, Zou Q. A comprehensive overview and evaluation of circular RNA detection tools. PLoS Computational Biology 2017;13(6).
44. Gao Y, Zhang J, Zhao F. Circular RNA identification based on multiple seed matching. Briefings in bioinformatics 2018;19(5):803–810.
45. Priestley P, Baber J, Lolkema MP, Steeghs N, de Bruijn E, Shale C, et al. Pan-cancer whole-genome analyses of metastatic solid tumours. Nature 2019;575(7781):210–216.
46. Alaei-Mahabadi B, Bhadury J, Karlsson JW, Nilsson JA, Larsson E. Global analysis of somatic structural genomic alterations and their impact on gene expression in diverse human cancers. Proceedings of the National Academy of Sciences of the United States of America 2016;113(48):13768–13773.
47. Yoshihara K, Wang Q, Torres-Garcia W, Zheng S, Vegesna R, Kim H, et al. The landscape and therapeutic relevance of cancer-associated transcript fusions. Oncogene 2015 sep;34(37):4845–4854. http://dx.doi.org/10.1038/onc.2014.406.
48. van Dessel LF, van Riet J, Smits M, Zhu Y, Hamberg P, van der Heijden MS, et al. The genomic landscape of metastatic castration-resistant prostate cancers reveals multiple distinct genotypes with potential clinical impact. Nature Communications 2019;10(1):5251. http://dx.doi.org/10.1038/s41467-019-13084-7.
49. Hamid Beniamin Petreaca A, Petreaca R. Frequent homozygous deletions of the CDKN2A locus in somatic cancer tissues. Mutation Research - Fundamental and Molecular Mechanisms of Mutagenesis 2019;815(January):30–40.
50. Tian L, Li Y, Edmonson MN, Zhou X, Newman S, McLeod C, et al. CICERO: A versatile method for detecting complex and diverse driver fusions using cancer RNA sequencing data. Genome Biology 2020;21(1):126.
51. Korbel JO, Campbell PJ. Criteria for inference of chromothripsis in cancer genomes. Cell 2013;152(6):1226–1236.
52. Govind SK, Zia A, Hennings-Yeomans PH, Watson JD, Fraser M, Anghel C, et al. ShatterProof: Operational detection and quantification of chromothripsis. BMC Bioinformatics 2014;15(1):78.
53. Menez-Jamet J, Gallou C, Rougeot A, Kosmatopoulos K. Optimized tumor cryptic peptides: The basis for universal neoantigen-like tumor vaccines. Annals of Translational Medicine 2016;4(14):266.
54. Gubin MM, Artyomov MN, Mardis ER, Schreiber RD. Tumor neoantigens: Building a framework for personalized cancer immunotherapy. Journal of Clinical Investigation 2015;125(9):3413–3421.
55. Hoogstrate Y, Zhang C, Senf A, Bijlard J, Hiltemann S, van Enckevort D, et al. Integration of EGA secure data access into Galaxy. F1000Research 2016;5(0):3–9.
56. Pleasance ED, Cheetham RK, Stephens PJ, McBride DJ, Humphray SJ, Greenman CD, et al. A comprehensive catalogue of somatic mutations from a human cancer genome. Nature 2010 jan;463(7278):191–196. http://www.nature.com/articles/nature08658.
57. Hiltemann S, Jenster G, Trapman J, Van Der Spek P, Stubbs A. Discriminating somatic and germline mutations in tumor DNA samples without matching normals. Genome Research 2015;25(9):1382–1390.
58. Zhang C, Bijlard J, Staiger C, Scollen S, van Enckevort D, Hoogstrate Y, et al. Systematically linking tranSMART, Galaxy and EGA for reusing human translational research data. F1000Research 2017;6.
59. Dobin A, Davis CA, Schlesinger F, Drenkow J, Zaleski C, Jha S, et al. STAR: Ultrafast universal RNA-seq aligner. Bioinformatics 2013;29(1):15–21.
60. Reimand J, Arak T, Adler P, Kolberg L, Reisberg S, Peterson H, et al. g:Profiler-a web server for functional interpretation of gene lists (2016 update). Nucleic acids research 2016;44(W1):W83–W89.
61. Robinson MD, McCarthy DJ, Smyth GK. edgeR: A Bioconductor package for differential expression analysis of digital gene expression data. Bioinformatics 2009;26(1):139–140.
62. Hendriksen PJM, Dits NFJ, Kokame K, Veldhoven A, Van Weerden WM, Bangma CH, et al. Evolution of the androgen receptor pathway during progression of prostate cancer. Cancer Research 2006;66(10):5012–5020.

Click here to access/download
Supplementary Material
Additional file 1 - Dr. Disco Technical Specification.docx

Click here to access/download
Supplementary Material
Additional file 3 - Supplementary Methods.docx

Click here to access/download
Supplementary Material
Table S1 - Results SRR534293.xlsx

Additional file 5 - Table S2 - gProfiler - Supplementary Material.xlsx

Click here to access/download
Supplementary Material
Table S2 - gProfiler.xlsx

Click here to access/download
Supplementary Material
Table S3 - SHANK2 and TENM4 related junctions.xlsx

Click here to access/download
Supplementary Material
Table S4 - TMPRSS2-ERG and related junctions.xlsx

Click here to access/download
Supplementary Material
Additional file 2 - Supplementary Figures_revised.pptx

Rebuttal letter

Click here to access/download;Personal Cover;rebuttal_letter.docx

Erasmus MC
Universitair Medisch Centrum Rotterdam

Ref.: GIGA-D-21-00236
Fusion transcripts and their genomic breakpoints in poly(A)+ and rRNA-minus RNA sequencing data
GigaScience

Rotterdam, October 07, 2021

Dear Hans Zauner,

Please find attached our re-revised manuscript "**Fusion transcripts and their genomic breakpoints in poly(A)+ and rRNA-minus RNA sequencing data**" by Youri Hoogstrate *et al*. which we would like to resubmit for publication in *GigaScience*.

We would like to thank the referees for critical assessment of manuscript. The issues that were raised are addressed pointwise following this letter. Furthermore, we have re-written the manuscript as *Technical Note* and therefore excluded the “Potential implications” section and included *bio.tools* and *SciCrunch.org* identifiers. We believe to have adequately addressed all issues raised by the referees and with these changes we hope our manuscript is suitable for publication in GigaScience.

Yours sincerely,

Youri Hoogstrate, PhD (on behalf of all co-authors)

Point-wise response reviewer comments

Reviewer #1:

Q: Can Dr. Disco be used for single sample analysis or is it preferentially used for bulk analysis?

A: Dr. Disco can be used for single sample analysis. We addressed this in the manuscript by adding:

“While only large datasets were analyzed in this study, the method is explicitly developed for single-sample analysis.”

Also the github landing page was updated accordingly.

Q: Please review the figure legend of Fig. S7A. Seems like there is a mix-up: "This figure is divided over 4 sub figures (6A-6D)."

A: This mix-up has been revised. Thanks.

Q: It is mentioned that the large search space requires a more stringent filtering. How does this reflect on the run time of the algorithm? Is the run time comparable to the other fusion callers?

A: Reviewer 1 asks whether the large search space and stringent filtering affect run time performance as compared to other fusion callers. The graph construction and deconvolution are the most time consuming part of the algorithm. Using the evaluation MCF-7 dataset, we observed that the performance of this module was 9 minutes and 48 and filtering 10 second(s). The integration part, in which all candidate entries are compared to share overlap and are annotated with corresponding gene names and sequence motifs from reference FASTA and GTF files, also took a considerable amount of time (5 minutes and 51 seconds). We compared this to Arriba because both post-process the output of STAR. According to the log files or Arriba, it took 5 minutes and 9 seconds to read the chimeric input and reference files. We therefore concluded that Dr. Disco took 949 seconds and Arriba 458 seconds, and Arriba was thus ~2x times faster. We have therefore added the following text to the manuscript:

“The time it took Dr. Disco to complete analysis after the STAR alignment was 949 seconds, 2.07 times slower compared with Arriba (458 seconds).”

And changed:

“In concordance with our expectations, the large search space required more conservative filtering.”

->

"This is in concordance with the expectation that analysing a larger search space requires more conservative filtering and takes more resources to complete."

Q: It is mentioned that the number of identified true positives is lower for Dr. Disco. What was the overall accuracy of the method?

A: The results in the initial draft assessed only the cumulative number of true positives. To address this comment, we extended Figure 2 by providing the ratio-of-TP compared to the total calls. From this analysis, the conclusion did not change; Dr. Disco is stringent, but of the calls it makes, a high proportion is true positive. However, JAFFA's TP ratio was better. Conversely, Arriba finds most fusions and thus most TP fusions, but with a considerably lower TP-ratio, often finding fusions involving rRNA genes. Nevertheless, this analysis has proven that more fusions are present within Chimeric STAR alignments than Dr. Disco finds and improvement of filters prompts future work. We have adjusted the results section and added the following discussion point:

"That both Arriba and Dr. Disco make use of STAR and that Arriba finds a higher number of true positives indicates that improving the filtering is an important future step, but care must be taken not to compromise Dr. Disco's true positive ratio."

Q: How does the method compare to other fusion calling pipelines that rely on the chimeric junction information file of STAR (e.g. STAR-Fusion, arriba, etc)?

A: We have analyzed the results of STAR based detector Arriba and attempted the same with STAR-Fusion. STAR-Fusion crashed after 619 minutes with the following error: "`died with ret 512 No such file or directory at /home/youri/.local/src/STAR-Fusion/PerlLib/Pipeliner.pm line 181. Pipeliner::run(Pipeliner=HASH(0x562737a6dca8)) called at /home/youri/.local/src/STAR-Fusion/STAR-Fusion line 797`". STAR-Fusion was thereafter excluded from evaluation.

Although the TP-ratio of Arriba detected fusions was limited, it had an impressively high sensitivity indicating that STAR can provide more detectable junctions than Dr. Disco (and other tools) did. We updated the results and added the following to the discussion:

"That both Arriba and Dr. Disco make use of STAR and that Arriba finds a higher number of true positives indicates that improving the filtering is an important future step, but care must be taken not to compromise Dr. Disco's true positive ratio."

Q: The difference in the number of identified genomic breakpoints between RNA-Seq and DNA-Seq is addressed and it is reasoned that only a small fraction of genomic rearrangements is expressed. It is well known that not all genomic rearrangements generate a corresponding fusion transcript and hence the overlap should be considerably smaller. However, the high number of detected genomic breakpoints by

WGS will also contain a high number of false positive calls due to the comparable low coverage of the WGS data and the high noise associated with structural variant calling. Hence, the ~7% might be an underestimation.

A: Reviewer 1 raises an interesting point by noting that there might be more than 7% of the breakpoints expressed because WGS results can be incomplete (false negatives) and noisy (false positives). We indeed suspect, as shown in figure S7A (junctions detected at chr11 in BrCa that were missed by WGS), that WGS results of the BrCa data are likely missing true genomic events. But the opposite may also be true, that this is an overestimation and WGS results were really stringent. In both directions, discussion regarding this issue is speculative as both assays are affected by 'noise' that we cannot proof unambiguously. We have therefore changed the discussion into:

"*Here, we confirm by utilizing Dr. Disco, that RNA-seq data can be used to reveal genomic breakpoints of expressed transcripts in an automated fashion. Detection was limited to approximately 7% of WGS detected breakpoints but markedly higher for the driver TMPRSS2-ERG fusion gene (85% detected; 100% presence). As the algorithm was conservative in detecting mRNA fusions, it is likely that genomic breakpoints were missed and the actual percentage is somewhat higher. Conversely, estimation of this percentage implies that WGS results offer the ground truth but these are also affected by noise, coverage and filter cut-offs, indicating this percentage is an approximation.*"

Q: "Dr. Disco detected 357 unique genomic breakpoints (45.8%)..." It is not quite clear to which result the percentage refers to. Please rephrase.

A: We have rephrased the sentence into: "Dr. Disco detected 357 unique genomic breakpoints which were only…"

Q: It is indicated that the number of identified genomic breakpoints differs significantly between the various cancer types. It is obvious from Fig.5 that the read depth differs significantly between the various data sets, as briefly addressed in the discussion. Thus, is there a correlation between the number of identified breakpoints and the read depth that might confound the result? Similar to the correlation analysis of Fig S12.

A: Reviewer 1 asks whether there is a correlation between the number of breakpoints and the read depth. This is indeed the case as presented in Figure 3B, in which systematically truncating the read depth of four samples resulted in an associated reduction of detected junctions. Therefore, reviewer 1 raises a valid point by noting that this confounds interpreting the comparison of average junctions per sample, per dataset which differ in coverage. Other factors such as read length and library preparation also contribute to this problem and therefore make it, unfortunately, unfeasible to perform a correction on this. In the initial manuscript, we have addressed this issue by stating:

“*These average numbers were not normalized for sequence depth as they are also confounded by differences in read length, stranding, RNA quality and library preparation.*”.

As this is not sufficiently detailed, we therefore have added the following:

“*These average numbers were not normalized for sequence depth as results are also influenced by dataset specific differences in read length, stranding, RNA quality and library preparation. Therefore, comparison of these average numbers of junctions is confounded by these factors.*”

We have also changed the following sentence:

“*This variation is in line with the omics-reported number of structural variants; low in colorectal cancer [ref] while high in breast cancer [ref,ref], but is influenced by sequencing depth, length and library preparation which vary per dataset.*”

into

“*This variation is in line with the omics-reported number of structural variants; low in colorectal cancer [ref] while high in breast cancer [ref,ref], but these differences are confounded by the influence of sequencing depth, length and library preparation which vary per dataset.*”

Q: Is there any support or validation for the identified junctions with at least one side located within an intergenic region (Fig. 6)?

A: Reviewer 1 asks whether there is any support or validation for the detected intergenic events. In the NGS-ProToCol prostate cancer dataset, three intergenic TMPRSS2-ERG variants were highlighted (Figure S19) supported by not only high covered genomic breakpoints but also additional cryptic exons fitting the junction and well covered introns. For validation, we have assessed the overlap of partial intergenic intronic junctions with WGS entries for the BrCa chromothripsis events visualized in Figure S18. This showed that 14/18 investigated entries had matching WGS results. For intergenic exonic junctions, we have no means for validation. Figure S18 was updated accordingly. The following sentences in the discussion:

“Here, we confirm by utilizing Dr. Disco, that RNA-seq data be used to reveal genomic breakpoints of expressed transcripts, including intergenic translocations, in an automated fashion. Detection was limited to approximately 7% of WGS detected breakpoints but markedly higher for the driver TMPRSS2-ERG fusion gene (85% detected; 100% presence).”

Into:

“Here, we confirm by utilising Dr. Disco, that RNA-seq data can be used to reveal genomic breakpoints of expressed transcripts in an automated fashion. Detection was limited to approximately 7% of WGS detected breakpoints but markedly higher for the driver TMPRSS2-ERG fusion gene (85% detected; 100% presence). As the algorithm was conservative in detecting mRNA fusions, it is likely that genomic

breakpoints were missed and the actual percentage is somewhat higher. Conversely, estimation of this percentage implies that WGS results offer the ground truth but these are also affected by noise, coverage and filter cut-offs, indicating this percentage is an approximation. The results commonly included intergenic junctions. For instance, three TMPRSS2-ERG fusions had their breakpoint located before ERG, supported by cryptic intergenic splice junctions and intergenic pre-mRNA coverage (Figure S19). Furthermore, intronic intergenic junctions in chromothripsis regions in three BrCa samples were in 14/18 cases validated by WGS junctions (Figure S18A)."

Reviewer #2:

Q: please re-phrase the sentence "..,Dr. Disco, that searchers for fusion transcripts without being restricted to splice junctions or annotated exons or genes." such that it is stated what Dr.Disco is using when searching (please, do not use "without being restricted")

A: We have rephrased the sentence to "We have developed an algorithm, Dr. Disco that searches for fusion transcripts by taking an entire reference genome into account as search space. This includes exons but also introns, intergenic regions and sequences that do not meet splice junction motifs."

Q: please re-phrase the sentence "These normal adjacent tissue samples were most likely..." something like "These normal looking adjacent..." because it may very well be that they are not normal even that they look like.

A: We have rephrased them into the following sentences:

"In two normal looking adjacent prostate samples, intronic and exonic junctions were found that were exactly identical to junctions in their matching malignant sample."

"These normal looking adjacent tissue samples were most likely contaminated with cancer cells (Figure S9B)."

Reviewer #3:

-
