# Supplementary material for: Fusion transcripts and their genomic breakpoints in polyadenylated and ribosomal RNA–minus RNA sequencing data
Source: Gigascience. 2021 Dec 9;10(12):giab080. doi: 10.1093/gigascience/giab080 (PMC8673554; doi:10.1093/gigascience/giab080)
Supplement: giab080_Response_to_Reviewer_Comments_Original_Submission [file giab080_response_to_reviewer_comments_original_submission.pdf]

Author's Response To Reviewer Comments

Close

Dear editor, Dear Chris,

Thank you so much for all your effort. I have re-submitted the latest version including the citation to the GigaDB and added the appropriate data reference to the bib file. I removed the Zenodo link because of redundancy with the GigaDB data.

I am not sure why the bibfile gives errors on your side, it works for me in both overleaf and texmaker. If you need support from my side converting the manuscript to your in-house latex style, please let me know how I can help or send me the error and maybe I can resolve the bib file issue.

Before final acceptance, could you cross-check one more time whether the author names also on the main manuscript meet the following list ():

Hoogstrate, Youri
Komor, Malgorzata A
Böttcher, René
Van Riet, Job
Van De Werken, Harmen JG
Van Lieshout, Stef
Hoffmann, Ralf
Van Den Broek, Evert
Bolijn, Anne S
Dits, Natasja
Sie, Daoud
Van Der Meer, David
Pepers, Floor
Bangma, Chris H
Van Leenders, Geert JLH
Smid, Marcel
French, Pim J
Martens, John WM
Van Workum, Wilbert
Van Der Spek, Peter J
Janssen, Bart
Caldenhoven, Eric
Rausch, Christian
De Jong, Mark
Stubbs, Andrew P
Meijer, Gerrit A
Fijneman, Remond JA
Jenster, Guido W

---

kind regards,

Youri

Close
