# Supplementary material for: Fusion transcripts and their genomic breakpoints in polyadenylated and ribosomal RNA–minus RNA sequencing data
Source: Gigascience. 2021 Dec 9;10(12):giab080. doi: 10.1093/gigascience/giab080 (PMC8673554; doi:10.1093/gigascience/giab080)
Supplement: giab080_Response_to_Reviewer_Comments_Revision_1 [file giab080_response_to_reviewer_comments_revision_1.pdf]

Author's Response To Reviewer Comments

Close

Rotterdam, October 07, 2021

Dear Hans Zauner,

Please find attached our re-revised manuscript "Fusion transcripts and their genomic breakpoints in poly(A)+ and rRNA-minus RNA sequencing data" by Youri Hoogstrate et al. which we would like to resubmit for publication in GigaScience.
We would like to thank the referees for critical assessment of manuscript. The issues that were raised are addressed pointwise following this letter. Furthermore, we have re-written the manuscript as Technical Note and therefore excluded the "Potential implications" section and included bio.tools and SciCrunch.org identifiers. We believe to have adequately addressed all issues raised by the referees and with these changes we hope our manuscript is suitable for publication in GigaScience.

Yours sincerely,

Youri Hoogstrate, PhD (on behalf of all co-authors)

Point-wise response reviewer comments
Reviewer #1:
Q: Can Dr. Disco be used for single sample analysis or is it preferentially used for bulk analysis?
A: Dr. Disco can be used for single sample analysis. We addressed this in the manuscript by adding:
"While only large datasets were analyzed in this study, the method is explicitly developed for single-sample analysis."
Also the github landing page was updated accordingly.

Q: Please review the figure legend of Fig. S7A. Seems like there is a mix-up: "This figure is divided over 4 sub figures (6A-6D)."
A: This mix-up has been revised. Thanks.

Q: It is mentioned that the large search space requires a more stringent filtering. How does this reflect on the run time of the algorithm? Is the run time comparable to the other fusion callers?
A: Reviewer 1 asks whether the large search space and stringent filtering affect run time performance as compared to other fusion callers. The graph construction and deconvolution are the most time consuming part of the algorithm. Using the evaluation MCF-7 dataset, we observed that the performance of this module was 9 minutes and 48 and filtering 10 second(s). The integration part, in which all candidate entries are compared to share overlap and are annotated with corresponding gene names and sequence motifs from reference FASTA and GTF files, also took a considerable amount of time (5 minutes and 51 seconds). We compared this to Arriba because both post-process the output of STAR. According to the log files or Arriba, it took 5 minutes and 9 seconds to read the chimeric input and reference files. We therefore concluded that Dr. Disco took 949 seconds and Arriba 458 seconds, and Arriba was thus ~2x times faster. We have therefore added the following text to the manuscript:
"The time it took Dr. Disco to complete analysis after the STAR alignment was 949 seconds, 2.07 times slower compared with Arriba (458 seconds)."
And changed:
"In concordance with our expectations, the large search space required more conservative filtering."
->
"This is in concordance with the expectation that analysing a larger search space requires more

conservative filtering and takes more resources to complete.”

Q: It is mentioned that the number of identified true positives is lower for Dr. Disco. What was the overall accuracy of the method?
A: The results in the initial draft assessed only the cumulative number of true positives. To address this comment, we extended Figure 2 by providing the ratio-of-TP compared to the total calls. From this analysis, the conclusion did not change; Dr. Disco is stringent, but of the calls it makes, a high proportion is true positive. However, JAFFA’s TP ratio was better. Conversely, Arriba finds most fusions and thus most TP fusions, but with a considerably lower TP-ratio, often finding fusions involving rRNA genes. Nevertheless, this analysis has proven that more fusions are present within Chimeric STAR alignments than Dr. Disco finds and improvement of filters prompts future work. We have adjusted the results section and added the following discussion point:
“That both Arriba and Dr. Disco make use of STAR and that Arriba finds a higher number of true positives indicates that improving the filtering is an important future step, but care must be taken not to compromise Dr. Disco's true positive ratio.”

Q: How does the method compare to other fusion calling pipelines that rely on the chimeric junction information file of STAR (e.g. STAR-Fusion, arriba, etc)?
A: We have analyzed the results of STAR based detector Arriba and attempted the same with STAR-Fusion. STAR-Fusion crashed after 619 minutes with the following error: “died with ret 512 No such file or directory at /home/youri/.local/src/STAR-Fusion/PerlLib/Pipeliner.pm line 181.
Pipeliner::run(Pipeliner=HASH(0x562737a6dca8)) called at /home/youri/.local/src/STAR-Fusion/STAR-Fusion line 797”. STAR-Fusion was thereafter excluded from evaluation.
Although the TP-ratio of Arriba detected fusions was limited, it had an impressively high sensitivity indicating that STAR can provide more detectable junctions than Dr. Disco (and other tools) did. We updated the results and added the following to the discussion:
“That both Arriba and Dr. Disco make use of STAR and that Arriba finds a higher number of true positives indicates that improving the filtering is an important future step, but care must be taken not to compromise Dr. Disco's true positive ratio.”

Q: The difference in the number of identified genomic breakpoints between RNA-Seq and DNA-Seq is addressed and it is reasoned that only a small fraction of genomic rearrangements is expressed. It is well known that not all genomic rearrangements generate a corresponding fusion transcript and hence the overlap should be considerably smaller. However, the high number of detected genomic breakpoints by WGS will also contain a high number of false positive calls due to the comparable low coverage of the WGS data and the high noise associated with structural variant calling. Hence, the ~7% might be an underestimation.
A: Reviewer 1 raises an interesting point by noting that there might be more than 7% of the breakpoints expressed because WGS results can be incomplete (false negatives) and noisy (false positives). We indeed suspect, as shown in figure S7A (junctions detected at chr11 in BrCa that were missed by WGS), that WGS results of the BrCa data are likely missing true genomic events. But the opposite may also be true, that this is an overestimation and WGS results were really stringent. In both directions, discussion regarding this issue is speculative as both assays are affected by ‘noise’ that we cannot proof unambiguously. We have therefore changed the discussion into:
“Here, we confirm by utilizing Dr. Disco, that RNA-seq data can be used to reveal genomic breakpoints of expressed transcripts in an automated fashion. Detection was limited to approximately 7% of WGS detected breakpoints but markedly higher for the driver TMPRSS2-ERG fusion gene (85% detected; 100% presence). As the algorithm was conservative in detecting mRNA fusions, it is likely that genomic breakpoints were missed and the actual percentage is somewhat higher. Conversely, estimation of this percentage implies that WGS results offer the ground truth but these are also affected by noise, coverage and filter cut-offs, indicating this percentage is an approximation.”

Q: "Dr. Disco detected 357 unique genomic breakpoints (45.8%)..." It is not quite clear to which result the percentage refers to. Please rephrase.
A: We have rephrased the sentence into: “Dr. Disco detected 357 unique genomic breakpoints which were only…”

Q: It is indicated that the number of identified genomic breakpoints differs significantly between the various cancer types. It is obvious from Fig.5 that the read depth differs significantly between the various data sets, as briefly addressed in the discussion. Thus, is there a correlation between the number of identified breakpoints and the read depth that might confound the result? Similar to the correlation analysis of Fig S12.

A: Reviewer 1 asks whether there is a correlation between the number of breakpoints and the read depth. This is indeed the case as presented in Figure 3B, in which systematically truncating the read depth of four samples resulted in an associated reduction of detected junctions. Therefore, reviewer 1 raises a valid point by noting that this confounds interpreting the comparison of average junctions per sample, per dataset which differ in coverage. Other factors such as read length and library preparation also contribute to this problem and therefore make it, unfortunately, unfeasible to perform a correction on this. In the initial manuscript, we have addressed this issue by stating:
"These average numbers were not normalized for sequence depth as they are also confounded by differences in read length, stranding, RNA quality and library preparation.".
As this is not sufficiently detailed, we therefore have added the following:
"These average numbers were not normalized for sequence depth as results are also influenced by dataset specific differences in read length, stranding, RNA quality and library preparation. Therefore, comparison of these average numbers of junctions is confounded by these factors."
We have also changed the following sentence:
"This variation is in line with the omics-reported number of structural variants; low in colorectal cancer [ref] while high in breast cancer [ref,ref], but is influenced by sequencing depth, length and library preparation which vary per dataset."
into
"This variation is in line with the omics-reported number of structural variants; low in colorectal cancer [ref] while high in breast cancer [ref,ref], but these differences are confounded by the influence of sequencing depth, length and library preparation which vary per dataset."

Q: Is there any support or validation for the identified junctions with at least one side located within an intergenic region (Fig. 6)?
A: Reviewer 1 asks whether there is any support or validation for the detected intergenic events. In the NGS-ProToCol prostate cancer dataset, three intergenic TMPRSS2-ERG variants were highlighted (Figure S19) supported by not only high covered genomic breakpoints but also additional cryptic exons fitting the junction and well covered introns. For validation, we have assessed the overlap of partial intergenic intronic junctions with WGS entries for the BrCa chromothripsis events visualized in Figure S18. This showed that 14/18 investigated entries had matching WGS results. For intergenic exonic junctions, we have no means for validation. Figure S18 was updated accordingly. The following sentences in the discussion:
"Here, we confirm by utilizing Dr. Disco, that RNA-seq data be used to reveal genomic breakpoints of expressed transcripts, including intergenic translocations, in an automated fashion. Detection was limited to approximately 7% of WGS detected breakpoints but markedly higher for the driver TMPRSS2-ERG fusion gene (85% detected; 100% presence)."
Into:
"Here, we confirm by utilising Dr. Disco, that RNA-seq data can be used to reveal genomic breakpoints of expressed transcripts in an automated fashion. Detection was limited to approximately 7% of WGS detected breakpoints but markedly higher for the driver TMPRSS2-ERG fusion gene (85% detected; 100% presence). As the algorithm was conservative in detecting mRNA fusions, it is likely that genomic breakpoints were missed and the actual percentage is somewhat higher. Conversely, estimation of this percentage implies that WGS results offer the ground truth but these are also affected by noise, coverage and filter cut-offs, indicating this percentage is an approximation. The results commonly included intergenic junctions. For instance, three TMPRSS2-ERG fusions had their breakpoint located before ERG, supported by cryptic intergenic splice junctions and intergenic pre-mRNA coverage (Figure S19). Furthermore, intronic intergenic junctions in chromothripsis regions in three BrCa samples were in 14/18 cases validated by WGS junctions (Figure S18A)."

Reviewer #2:
Q: please re-phrase the sentence "..,Dr. Disco, that searchers for fusion transcripts without being restricted to splice junctions or annotated exons or genes." such that it is stated what Dr.Disco is using when searching (please, do not use "without being restricted")
A: We have rephrased the sentence to "We have developed an algorithm, Dr. Disco that searches for fusion transcripts by taking an entire reference genome into account as search space. This includes exons but also introns, intergenic regions and sequences that do not meet splice junction motifs."

Q: please re-phrase the sentence "These normal adjacent tissue samples were most likely..." something like "These normal looking adjacent..." because it may very well be that they are not normal even that they look like.
A: We have rephrased them into the following sentences:
"In two normal looking adjacent prostate samples, intronic and exonic junctions were found that were

exactly identical to junctions in their matching malignant sample."
"These normal looking adjacent tissue samples were most likely contaminated with cancer cells (Figure S9B)."

Reviewer #3:
-

Close
