# Supplementary material for: Fusion transcripts and their genomic breakpoints in polyadenylated and ribosomal RNA–minus RNA sequencing data
Source: Gigascience. 2021 Dec 9;10(12):giab080. doi: 10.1093/gigascience/giab080 (PMC8673554; doi:10.1093/gigascience/giab080)
Supplement: giab080_Reviewer_1_Report_Original_Submission — Wencke Walter, Ph.D. -- 8/30/2021 Reviewed [file giab080_reviewer_1_report_original_submission.pdf]

Reviewer Report

Title: Fusion transcripts and their genomic breakpoints in poly(A)+ and rRNA-minus RNA sequencing data

Version: Original Submission **Date:** 8/30/2021

Reviewer name: Wencke Walter, Ph.D.

Reviewer Comments to Author:

The study by Hoogstrate et al. comprehensively assesses the detection of genomic breakpoints by RNA-Seq (polyA Lib prep and rRNA depletion approach) and its correlation with WGS results. Comparing the two main approaches for RNA-Seq library preparation they identify a high concordance for exonic junctions but an advantage of rRNA depletion data for the detection of intronic junctions, as expected. Detected genomic breakpoints based on WGS data significantly outnumbered detected breakpoints by RNA-Seq. Various datasets were used to assess the performance of the developed method (Dr.Disco) to report genomic breakpoints and to identify fused cryptic exons, chromothripsis, and circular RNAs. The manuscript is generally well written, clear and adequately structured. The installation and application of the developed method is also well documented (github). My comments are the following:
- Can Dr. Disco be used for single sample analysis or is it preferentially used for bulk analysis?
- Please review the figure legend of Fig. S7A. Seems like there is a mix-up: "This figure is divided over 4 sub figures (6A-6D)."

Page 4, "Evaluation Poly(A)+ detectors":
- It is mentioned that the large search space requires a more stringent filtering. How does this reflect on the run time of the algorithm? Is the run time comparable to the other fusion callers?
- It is mentioned that the number of identified true positives is lower for Dr. Disco. What was the overall accuracy of the method?
- How does the method compare to other fusion calling pipelines that rely on the chimeric junction information file of STAR (e.g. STAR-Fusion, arriba, etc)?

Page 5, "Comparison of RNA- with DNA-seq data":
- The difference in the number of identified genomic breakpoints between RNA-Seq and DNA-Seq is addressed and it is reasoned that only a small fraction of genomic rearrangements is expressed. It is well known that not all genomic rearrangements generate a corresponding fusion transcript and hence the overlap should be considerably smaller. However, the high number of detected genomic breakpoints by WGS will also contain a high number of false positive calls due to the comparable low coverage of the WGS data and the high noise associated with structural variant calling. Hence, the ~7% might be an underestimation.
- "Dr. Disco detected 357 unique genomic breakpoints (45.8%)..." It is not quite clear to which result the percentage refers to. Please rephrase.

Page 7, "Pan-cancer analysis":
- It is indicated that the number of identified genomic breakpoints differs significantly between the various cancer types. It is obvious from Fig.5 that the read depth differs significantly between the

various data sets, as briefly addressed in the discussion. Thus, is there a correlation between the number of identified breakpoints and the read depth that might confound the result? Similar to the correlation analysis of Fig S12.
- Is there any support or validation for the identified junctions with at least one side located within an intergenic region (Fig. 6)?

Level of Interest

Please indicate how interesting you found the manuscript: Choose an item.

Quality of Written English

Please indicate the quality of language in the manuscript: Choose an item.

Declaration of Competing Interests

Please complete a declaration of competing interests, considering the following questions:

- Have you in the past five years received reimbursements, fees, funding, or salary from an organisation that may in any way gain or lose financially from the publication of this manuscript, either now or in the future?
- Do you hold any stocks or shares in an organisation that may in any way gain or lose financially from the publication of this manuscript, either now or in the future?
- Do you hold or are you currently applying for any patents relating to the content of the manuscript?
- Have you received reimbursements, fees, funding, or salary from an organization that holds or has applied for patents relating to the content of the manuscript?
- Do you have any other financial competing interests?
- Do you have any non-financial competing interests in relation to this paper?

If you can answer no to all of the above, write 'I declare that I have no competing interests' below. If your reply is yes to any, please give details below.

I declare that I have no competing interests.

I agree to the open peer review policy of the journal. I understand that my name will be included on my report to the authors and, if the manuscript is accepted for publication, my named report including any attachments I upload will be posted on the website along with the authors' responses. I agree for my report to be made available under an Open Access Creative Commons CC-BY license (http://creativecommons.org/licenses/by/4.0/). I understand that any comments which I do not wish to be included in my named report can be included as confidential comments to the editors, which will not be published.

Choose an item.

To further support our reviewers, we have joined with Publons, where you can gain additional credit to further highlight your hard work (see: https://publons.com/journal/530/gigascience). On publication of this paper, your review will be automatically added to Publons, you can then choose whether or not to claim your Publons credit. I understand this statement.

Yes Choose an item.
